# Technical Insights into Highly Sensitive Isolation and Molecular Characterization of Fixed and Live Circulating Tumor Cells for Early Detection of Tumor Invasion

**DOI:** 10.1371/journal.pone.0169427

**Published:** 2017-01-06

**Authors:** Sophie Laget, Lucile Broncy, Katia Hormigos, Dalia M. Dhingra, Fatima BenMohamed, Thierry Capiod, Magne Osteras, Laurent Farinelli, Stephen Jackson, Patrizia Paterlini-Bréchot

**Affiliations:** 1 Rarecells Diagnostics, Paris, France; 2 Unité INSERM U1151 (Eq 13), Faculté de Médecine Paris Descartes, Paris, France; 3 Thermo Fisher Scientific, South San Francisco, California, United States of America; 4 Fasteris SA, Geneva, Switzerland; National Cancer Center, JAPAN

## Abstract

Circulating Tumor Cells (CTC) and Circulating Tumor Microemboli (CTM) are Circulating Rare Cells (CRC) which herald tumor invasion and are expected to provide an opportunity to improve the management of cancer patients. An unsolved technical issue in the CTC field is how to obtain highly sensitive and unbiased collection of these fragile and heterogeneous cells, in both live and fixed form, for their molecular study when they are extremely rare, particularly at the beginning of the invasion process. We report on a new protocol to enrich from blood live CTC using ISET^®^ (Isolation by SizE of Tumor/Trophoblastic Cells), an open system originally developed for marker-independent isolation of fixed tumor cells. We have assessed the impact of our new enrichment method on live tumor cells antigen expression, cytoskeleton structure, cell viability and ability to expand in culture. We have also explored the ISET^®^ *in vitro* performance to collect intact fixed and live cancer cells by using spiking analyses with extremely low number of fluorescent cultured cells. We describe results consistently showing the feasibility of isolating fixed and live tumor cells with a Lower Limit of Detection (LLOD) of one cancer cell per 10 mL of blood and a sensitivity at LLOD ranging from 83 to 100%. This very high sensitivity threshold can be maintained when plasma is collected before tumor cells isolation. Finally, we have performed a comparative next generation sequencing (NGS) analysis of tumor cells before and after isolation from blood and culture. We established the feasibility of NGS analysis of single live and fixed tumor cells enriched from blood by our system. This study provides new protocols for detection and characterization of CTC collected from blood at the very early steps of tumor invasion.

## Introduction

The most challenging goal in the Circulating Tumor Cells (CTC) field is their unbiased and reliable detection when they are extremely rare, namely at the beginning of the invasion process. At clinical level, this goal implies the possibility to detect invasive cancers when they are still curable, raising the hope of tremendously reducing cancer mortality [1–4]. At biological level, the initial spread of CTC may provide an outstanding source of material to understand the biology of early tumor invasion. Furthermore, high sensitivity is needed to obtain a sufficient number of tumor cells for theranostic analyses.

In this setting, technical challenges remain to be addressed and rigorous *in vitro* performance validations are required targeting unbiased isolation and detection of CTC when they are very rare, due to their low abundance, fragility, heterogeneity and lack of specific markers [2].

Approximately, forty different CTC isolation/detection methods have been published [5–9]. To our knowledge, however, no report specifically addresses the analytical issues of the use of these technologies for the purpose of early detection of invasive cancers. This implies the isolation without bias of selection and the identification without mistake of the very rare CTC that are spread at the beginning of the tumor invasion process. Early detection of aggressive cancers also implies studying the immune-molecular profile of the rare CTC as well as their growth potential.

CTC populations consist of cancer cells with very different phenotypes, including epithelial tumor cells, mesenchymal tumor cells, epithelial to mesenchymal hybrid tumor cells, stem tumor cells and clusters of tumor cells called Circulating Tumor Microemboli (CTM) [2, 4, 10–13]. Furthermore, identification of cancer cells in blood is challenging because of their similarities to non-tumor Circulating Rare Cells (CRC) such as circulating epithelial-normal cells, epithelial-atypical cells, endothelial cells, normal stem cells and physiological-state dependent cells (such as giant monocytes, micromegakaryocytes and fetal cells in pregnant and ex-pregnant women) [2]. Taking into account the vast heterogeneity of circulating rare cells and the lack of circulating tumor cells-specific markers, the use of epithelial and/or organ specific antibodies at the isolation/enrichment step or for the identification of CTC may lead to selection/detection biases [2, 4, 13–15].

In 2000, we reported on ISET^®^ (Isolation by SizE of Tumor/Trophoblastic Cells), the first antibody-independent whole blood filtration-based approach for CTC isolation. This method relies on the larger size of all types of CRC as compared to the majority of leukocytes [10]. ISET^®^ is performed within 5 hours after blood collection and carefully preserves the cell morphology. When combined with cytopathology, the filtration method has been shown to allow distinguishing circulating malignant cells derived from practically all types of solid tumors from circulating benign cells including those derived from organs [2, 4, 16].

Several studies have shown the feasibility of characterizing CTC isolated by ISET^®^ using simple or multiple immuno-fluorescence labeling [11, 17, 18], simple or multiple immuno-cytochemistry labeling [10, 12, 19, 20], FISH analyses [10, 21–23] and targeted molecular analyses [10, 17, 24–26].

ISET^®^’s sensitivity threshold (lower limit of detection (LLOD)) was initially determined at one tumor cell per mL of blood using a prototype [10], a result which has been subsequently confirmed by an independent team [25]. In 2006, our team has developed a device and consumables (ISET^®^ System) specifically designed to make our approach reproducible in other laboratories. Independent teams have since then confirmed its *in vitro* LLOD of one tumor cell per mL of blood [12, 27–29] and shown its *in vivo* superior sensitivity [3], including in comparative tests [12, 17, 20, 22, 23, 30–32].

We report here the careful assessment of the ISET^®^ System's *in vitro* analytical performance. We have studied its LLOD as well as its sensitivity at the sensitivity threshold (LLOD). Until now, studies have only demonstrated the ISET^®^ System’s ability to isolate fixed CTC capturing them attached to filters. However, their sensitive enrichment as living cells is required for a deeper investigation of their molecular code and to explore their possible use for culture and drug sensitivity assays. In the present report a new variant protocol of the ISET^®^ System to enrich live CTC from blood and its analytical assessment is demonstrated. Our results show that our system and protocols allow collecting fixed and live CTC from blood with a LLOD of one CTC per 10 mL of blood. We also show that live tumor cells isolated from blood using the new protocol maintain their initial antigen expression levels, their viability and ability to grow. Finally, we compared by next generation sequencing (NGS) molecular profiles of tumor cells before and after isolation from blood and culture. We address the feasibility of analyzing by NGS the genetic code of individual cells enriched from blood by our system.

These results and developments should foster clinical and fundamental studies targeting the very early steps of tumor cells invasion.

## Material and Methods

### 1- Blood samples collection

Blood from healthy volunteers was obtained from the French Blood Bank (Etablissement Français du Sang) according to the local ethics rules (agreement number 2014000051/U1151).

Blood samples were drawn preferentially in liquid K_3_EDTA tubes (Becton Dickinson, USA) with immediate gentle agitation after blood collection. If samples were not processed immediately after blood withdrawal, the tubes were left on a blood agitator until processing within 5 hours after blood collection.

### 2- Cell lines, cell size analysis of cells from cell lines

A549 (human lung adenocarcinoma), HeLa (human cervical epithelioid carcinoma), MCF-7 (human breast adenocarcinoma) and LNCaP (human prostate adenocarcinoma) cells were obtained from American Type Culture Collection (ATCC, USA). Mouse MMTV-PyMT cells, obtained by mammary gland-specific expression of the polyomavirus middle T antigen [33], were a kind gift from Dr. Takemi Tanaka (Oklahoma University, USA). H_2_/H_3_-GFP-HCT116 cells (human colon cancer) were a kind gift from Dr. Guido Kroemer (Institut Gustave Roussy, France).

A549, LNCaP and MMTV-PyMT cells were cultured in RPMI media supplemented with 10% Fetal Calf Serum (FCS) and 1% penicillin-streptomycin (P/S). MCF-7 cells were grown in DMEM media supplemented with 10% FCS and 1% P/S. HCT116 were grown in MacCoy media supplemented with 10% FCS and 1% P/S. All cells were cultured at 37°C with 5% CO_2_, and passaged regularly as previously described [10, 34].

For cell size analysis on ISET^®^ filters, trypsin-treated cultured cells were fixed with the Rarecells^®^ Buffer (ref. 54 0203) for 10 minutes, when mixed with blood, or for 3 minutes without blood and filtered through ISET^®^ filters with a nominal pore size of 8 or 5 μm. Cells on filters were stained with the May-Grünwald Giemsa (MGG) cytopathological staining, as described previously [35]. Analysis of cell size on ISET^®^ filters was performed with an Eclipse microscope (Nikon, Japan) equipped with the Cell Cut System using the pre-calibrated software (Molecular Machines and Industries, Germany). The size of at least 100 individual MGG-stained cells was analyzed for each cell line.

### 3- Isolation from blood of fixed tumor cells using the ISET^®^ system

**Fig 1:**
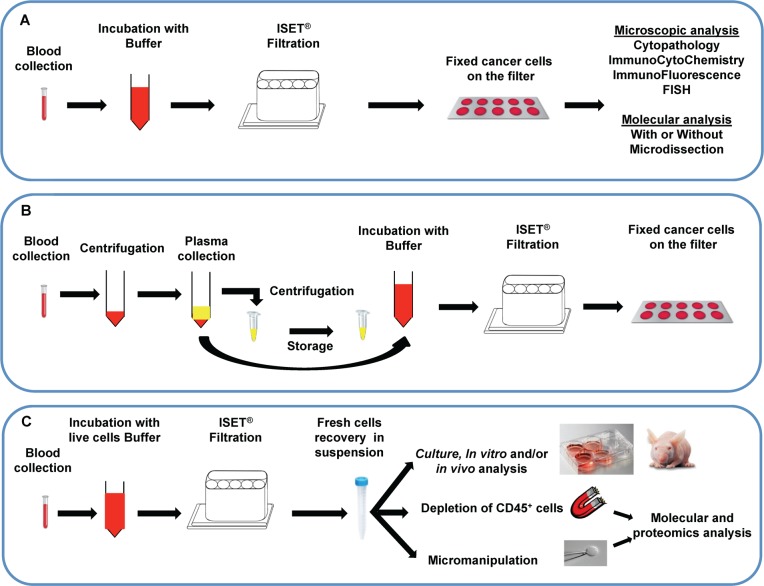
Overview of ISET^®^ filtration workflows. (A) ISET^®^ workflow for isolation and downstream analysis of fixed Circulating Rare Cells (CRC) from 10 mL of whole blood (See Methods section 3 for details). The filter can subsequently be sent by post or stored in biobank for years [21] for further CRC staining, cyto-morphological analysis and counting, immuno-labeling, *in situ* hybridization and molecular analyses (with or without laser capture microdissection). (B) ISET^®^ workflow for dual collection of plasma and enrichment of fixed CRC from whole blood (See Methods, section 4 for details). (C) ISET^®^ workflow for enrichment and downstream analysis of live CRC from whole blood (See Methods, section 5 for details). Optionally, single-cells can be isolated by micromanipulation for further analyses or CRC can be purified by immune-magnetic depletion of CD45^+^ cells before further molecular or cell growth assays.

The CE-IVD labeled Rarecells^®^ Device and its consumables (Rarecells Diagnostics, France) were used to assess the *in vitro* performance of the ISET^®^ standard protocol for marker-independent isolation of fixed tumor cells from blood (Fig 1A). The Rarecells^®^ Buffer solution (ref. 54 0203) was reconstituted according to the manufacturer instructions. The reconstituted buffer solution can be stored frozen at -20°C for up to 6 months. Before use, the pH is adjusted to 7.2. Formaldehyde is then added for cell fixing and to obtain a 0.74% final concentration. 90 mL of Rarecells^®^ buffer is then used to dilute ten mL of blood (10-fold dilution) to prepare it for filtration. Blood pretreatment has to take place for exactly 10 minutes under constant gentle stirring on a horizontal mixer (CAT Ingenieurbüro model# RM5-40).

A disposable cartridge (Rarecells^®^ Block) containing a filter having proprietary characteristics and 8 microns nominal pores size is set into the device. The cartridge contains 6 compartments: a large compartment for filtration of 5 mL of blood and 5 smaller compartments, each one for filtration of 1 mL of blood. The compartments are independent, allowing filtering variable volumes of blood from 10 μL to 10 mL of blood (100 μL to 100 mL of diluted blood). Empty compartments have to be closed during filtration with Rarecells^®^ Block lids.

The protocol starts with filtration of 50 mL of sterile PBS to hydrate the filter, then the diluted blood is loaded (100 mL of 1:10 diluted blood) into the Rarecells^®^ Block and filtered at a typical standard calibrated depression of -10 kPa. Blood filtration lasts no longer than a minute. Since the degree of blood cellularity may be variable due to physiological or pathological conditions and may increase blood resistance to filtration, the device allows increasing the depression for a few seconds if needed in order to complete filtration. This method allows maintaining a minimum shear force and sticks the cells to the filter avoiding their loss. The tubes that contained the diluted blood are then rinsed with 100 mL of sterile PBS that is also filtered in a few seconds. The cartridge is released from the device and disassembled in order to extract and gently rinse the ISET^®^ filter with sterile distilled water. Fixed cells are left to dry and attach firmly to the filter at room temperature for 15 to 30 minutes.

The filter contains 10 circular areas (spots) and each spot contains the CRC that were, before filtration, in one mL of blood along with some residual leukocytes.

The technical characteristics of the Block allow processing 1 to 10 mL of blood by ISET^®^, while the number of spots still corresponds to the number of milliliters of blood filtered. The counting of the number of CTC per mL then conveniently corresponds to the number of spots counted.

#### Tumor cells distribution (intra-assay precision and accuracy) analyses

In order to verify the random distribution of CTC on the five spots located in the large compartment of the Rarecells^®^ Block, cultured A549 cells were made fluorescent and precisely counted as described in section 6. An aliquot of 100 μL of the diluted suspension containing 50 cells was added to 5 mL of healthy donor's blood and processed by filtration (Fig 1A).

The number of tumor cells found on each spot (each corresponding to the filtration of 1 mL of blood) was recorded.

For each experiment, we calculated the average number of tumor cells found if considering randomly only 1 spot, only 2 spots, only 3 spot and only 4 spots. We analyzed all possible single spots or randomly grouped spots (any 1, any 2, any 3, any 4 spots) and calculated corresponding mean values and their standard deviations. Intra-assay precision (percent coefficient of variation, %CV) and accuracy (%Error) were calculated (formula given in section 6).

#### Counting of residual leukocytes

In order to evaluate the number of remaining White Blood Cells (WBC), one mL of blood from four different healthy donors was filtered and stained with Hematoxylin and Eosin. Leucocytes were then carefully counted under microscope.

### 4- Dual collection of plasma and fixed tumor cells

**Fig 2:**
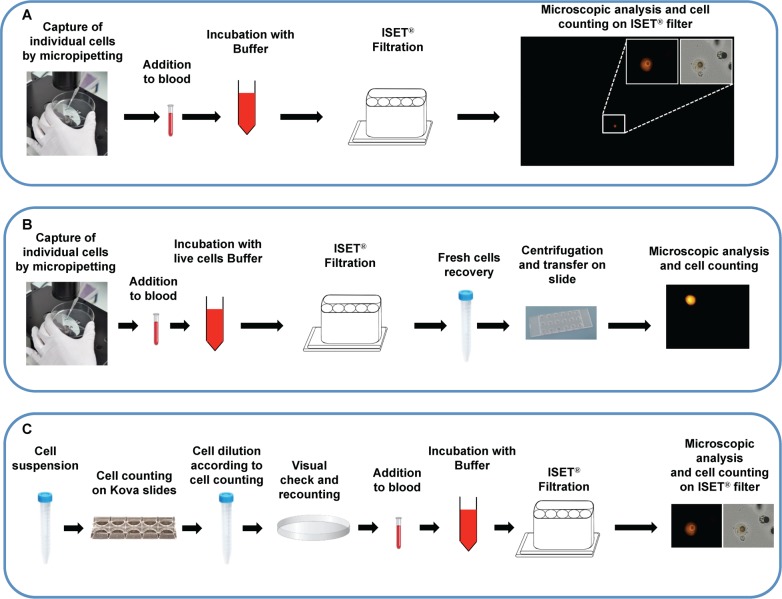
ISET^®^ sensitivity *in vitro* assays using individually micropipetted tumor cells. (A) *In vitro* sensitivity assay using the ISET^®^ workflow described in Fig 1A (See Methods sections 3 and 6 for details). Image: example of recovered fixed cell stained with Cell Tracker^TM^ Orange (larger panel: TRITC filter only, lower magnification, or smaller panels (higher magnification, TRITC filter only or merge of TRITC filter and brightfield)). (B) *In vitro* sensitivity assay using the ISET^®^ workflow described in Fig 1C (See Methods, sections 5 and 6 for details). Image: example of recovered live cell stained with Cell Tracker^TM^ Orange (TRITC filter). (C) *In vitro* sensitivity assay using the ISET^®^ workflow described in Fig 1A (See Methods sections 3 and 6 for details) with precise counting of cells by dilution. Image: example of recovered fixed cell stained with Cell Tracker^TM^ Orange (TRITC filter only or merge of TRITC filter and brightfield)

We have assessed the feasibility of recovering plasma from whole blood, collected on EDTA as previously described, without losing fixed CTC (Fig 1B). Fluorescent tumor cells were added to blood by individual cell micropipetting according to the protocol described in section 6 and Fig 2A. Two aliquots of 5 mL of blood were transferred into 2 x 50 mL Falcon tubes and centrifuged at 150 g for 10 minutes and 2 x 1 mL of the upper phase (plasma) were carefully pipetted without disturbing the interphase and transferred to a micro-centrifuge tube. The collected plasma was then centrifuged at 15000 g for 10 minutes (to eliminate potential debris), the supernatant carefully transferred to a new microcentrifuge tube and stored at -20°C for further analyses. The remaining 2 x 4 mL of blood were then carefully diluted to 50 mL volume each with the Rarecells^®^ Buffer (ref 54 0203) and mixed by a gentle and slow inversion of the tube at least 20 times. The diluted blood was then left for 10 minutes under horizontal mild agitation and processed by the ISET^®^ system to isolate fixed tumor cells as described above.

### 5- Isolation from blood of live tumor cells and their characterization: tumor cells’ size, antigen expression, viability, culture and study of cytoskeleton markers

The Rarecells^®^ Device and Block were used with a new protocol developed to obtain the marker-independent enrichment of live tumor cells from blood without sticking them to the filter (Fig 1C) and its performance was assessed. The Rarecells^®^ Live Cells Buffer solution (ref. 54 0208) was reconstituted according to the manufacturer instructions (Rarecells Diagnostics, France). This solution can be used for up to a week if stored at 4°C and for up to 6 months if frozen and stored at -20°C.

Blood was diluted 10 fold with the Rarecells^®^ Live Cells Buffer and kept at room temperature for 5 minutes under gentle constant stirring. The diluted blood (its color becomes bright ruby) was then transferred to the Rarecells^®^ Block and filtered slowly using a minimal depression force (typically—3 to—6 kPa). When about 200–500 μL of diluted blood remained in the wells, the filtration was stopped. The tubes that contained the diluted blood were rinsed with 13 mL of sterile PBS and the rinse solution was filtered with the device leaving again 500 μL of liquid into the wells. To obtain this, the depression was stopped by closing the tap and turning off the device so that the cells remained in suspension (not attached to the filter). The walls of each well were further rinsed 3 times by filtration with 1 mL of PBS (or culture media) supplemented with 2% FCS and 3 mM EDTA, again leaving 500 μL of liquid into the well. Finally, the liquid containing the enriched tumor cells (500 μL) was very carefully transferred with a micropipette from the cartridge to a tube or cell culture microplate.

Optionally, live tumor cells enriched from blood by ISET^®^ were concentrated by centrifugation for 4 minutes at 300 g. The supernatant was carefully eliminated leaving 20 to 30 μL of cell suspension, which can be transferred to an 18 well-flat microchamber (Ibidi, Germany) for microscopy evaluation and cell counting or other downstream analyses such as short-term culture.

Optionally, the enriched live cells can be collected with a suitable fixative for downstream analyses or with a storage fixative (Rarecells^®^ Fixcells, Rarecells Diagnostics, France) allowing their storage at + 4°C for up to 4 weeks.

Optionally, tumor cells can be isolated in suspension as fixed cells (without sticking them to the filter). To this aim, we diluted the blood 1:10 with the Rarecells^®^ Buffer (ref. 54 0203). After mild agitation for 10 min, we applied the same filtration protocol described for enrichment of live tumor cells and collected the fixed tumor cells.

#### Counting of residual leukocytes

We assessed the number of remaining leukocytes after live tumor cells enrichment using 2 x 5 mL of blood obtained from 2 distinct healthy donors in 2 separate tests, and using a TC20™ Automated Cell Counter (Bio Rad, USA) after cell staining with Trypan Blue.

#### Elimination of residual leukocytes after live tumor cells enrichment by ISET^®^

We tested the feasibility of eliminating the residual leucocytes after live tumor cells enrichment by the ISET^®^ system. To this aim, the live tumor cells samples obtained with the ISET^®^ system were treated with the manual column-free method EasySep^TM^ and the ‘Human CD45 Depletion kit’ (StemCell Technologies, Canada) that contains a combination of monoclonal antibodies against the leucocyte marker CD45 [36]. This kit is originally intended for use with whole blood after Ficoll-type density centrifugation, a step that is known to lead to highly significant tumor cells loss [37, 38]. In order to adapt this kit to the protocol for isolation of live tumor cells by ISET^®^, the amount of antibody and nanoparticles was reduced by 4-fold while the other manufacturer’s recommendations were followed. In brief, CD45 positive cells were specifically labeled with dextran-coated magnetic nanoparticles using bispecific Tetrameric Antibody Complexes (TAC). Tumor cells were then recovered after two successive CD45 depletions using the EasySep^TM^ Purple Magnet.

#### Live tumor cells' number, size and viability after their enrichment by ISET^®^

Cells were prepared and carefully counted as described in section 6. A fraction of the cell suspension (typically 100 μL) was mixed with 1 mL of blood from a healthy donor, or 1 mL of PBS, diluted tenfold with the Rarecells^®^ Live Cells Buffer and kept at room temperature for 5 minutes, or 3 minutes when cells were treated without blood, under gentle constant stirring. Filtration was then performed with the new protocol as described above. For comparison of cell counts, sizes and viability on the TC20™ Automated Cell Counter (Bio Rad, USA), 10 μL of either the unfiltered cells or the enriched fraction recovered after filtration were diluted 1:2 with Trypan Blue and loaded into a counting slide (Dual chamber for cell counter, BioRad, USA). Three or more counts were performed to obtain consistent counting values.

#### Live tumor cells antigen expression assessment before and after their enrichment by ISET^®^

We assessed the potential impact of this new ISET^®^ filtration protocol on cell surface antigens' expression by fluorescent labeling of the Epithelial Cell Adhesion Marker (EpCAM) on MCF-7 cultured cells before and after live cell enrichment by ISET^®^. MCF-7 cells in culture were first incubated with 5 μg/mL of Hoechst 33342 (Sigma-Aldrich, USA) for 2 hours before being rinsed twice with PBS, detached from the flask by incubation with trypsin (0.125%) and resuspended in 5 mL of culture media as described in section 6. A fraction of the cell suspension (typically 100 μL) was mixed with 1 mL of blood from a healthy donor, diluted 1:10 with the Rarecells^®^ Live Cells Buffer and kept at room temperature for 5 minutes under gentle constant stirring. The enriched fraction recovered after live cells filtration was centrifuged for 2 minutes at 150 g. The supernatant was carefully eliminated leaving 100 μL of suspension. Fluorescent labeling of EpCAM was performed on both the unfiltered cells and the enriched fraction recovered after ISET^®^ filtration. 20 μL of Human FcR binding inhibitor (eBioscience, USA) were added to each 100 μL suspension and kept in the dark at 4°C for 20 minutes. The Alexa Fluor 488 conjugated anti-EpCAM antibody clone VU1D9 (Cell Signaling Technology, USA) was diluted 1 to 30 in PBS and 20 μL of that dilution were then added to each sample followed by a 30 minutes incubation in the dark at 4°C. After washing with 1 mL of PBS, the cells were centrifuged at 300 g for 4 minutes and transferred to a microchamber (Ibidi, Germany). Microscopy evaluation was performed with an epi-fluorescence microscope (Zeiss, Germany). The DAPI filter (emission at 465 nm) and the FITC filter (emission at 525 nm) were used for the detection of the Hoechst and the EpCAM signals, respectively. Images were processed with Image J software. To perform quantitative analysis of EpCAM fluorescence, we analyzed two consecutive optical fields and measured the cell mean fluorescence, the cell area and the integrated density (fluorescence per area) for each cell (50 MCF-7 cells before and 50 MCF-7 cells after live cell enrichment). We then calculated the corrected total cell fluorescence (CTCF).

*CTCF = Integrated_density–(Area_of_selected_cell x mean_background_fluorescence)*. Furthermore we applied cutoffs at 91000 units and 200000 units of CTCF based on the calculations of the first and third quartiles of each data set.

#### Live tumor cells growth after their isolation by ISET^®^

We performed *in vitro* culture experiments of live tumor cells enriched from blood by ISET^®^. In two separate tests with 3 replicates, 10^4^ A549 cells were added to 1 mL of healthy donor’s blood before treatment with the Rarecells^®^ Live Cells Buffer as described above and in section 6. Samples were incubated for 5 min before filtration as described above.

The enriched cells were recovered and centrifuged for 2 minutes at 150 g. After removal of the supernatant, each pellet was carefully resuspended in 100 μL of complete media, deposited onto a round glass coverslip of 1.2 cm in diameter (MGF-slides, Microscopic Glass Factory) and placed in a 24-wells culture plate (Multiwell^TM^ 24, Becton Dickinson, USA). Cells were left to attach for 2 hours in a culture incubator. Coverslips were then washed with 300 μL of PBS and the cells were either collected for immediate microscopy observation (D0 time points) or supplemented with fresh media and kept in culture for 48 hours (D2 time points) or up to 5 days (D5 time points) before observation.

#### Confocal microscopy analysis of cytoskeleton markers before enrichment and after enrichment by ISET^®^ and culture for 72 hours

To assess the potential impact of our live tumor cells isolation protocol on cytoskeleton structures, we used cytoskeleton markers and immunofluorescence staining of actin microfilaments and microtubules together with confocal microscopy. Live A549 and H2228 tumor cells were seeded onto round glass coverslips and placed in a 24-wells culture plate before and after live cell enrichment from blood by ISET^®^, as described above. Cells were kept in culture for 72h before fixation by incubating 15 minutes with a 4% paraformaldehyde solution. Cells were then washed twice with PBS and permeabilized 15 minutes in PBS containing 0,2% Triton X-100 before washing again twice with PBS. Antigens were then blocked by incubating with PBS containing 10% FCS for 1h at room temperature. Primary mouse antibody targeting human actelyl-α-Tubulin (6-11-B1 clone, Thermofisher Scientific, USA) was diluted 1:200 in PBS containing 10% FCS and incubated 1h at room temperature. Cells were then washed twice with PBS and incubated 30 minutes in the dark with a 1:200 dilution of the secondary goat anti mouse antibody conjugated to a Dylight 488 fluorophore (Thermofisher Scientific, USA). Without washing, samples were then supplemented with Alexa Fluor 568 conjugated Phalloidin (Thermofisher Scientific, USA) for F-actin labeling at 20 units/mL and kept in the dark for another 30 minutes at room temperature. Cells were then washed three times in PBS and incubated 15 minutes at room temperature and in the dark with PBS containing 8 μM Hoechst for nuclear staining. After rinsing twice with PBS, coverslips were finally mounted on glass slides using Ultramount permanent mounting medium (Dako, USA). Microscopy evaluation was performed with an SP5 confocal microscope (Leica, Germany) and all images were acquired with identical parameter settings. Images were processed with Image J software and the quantitative analysis of both actin and tubulin fluorescence was performed on 30 cells for each sample evaluated by calculating the corrected total cell fluorescence (CTCF) of each signal as described above.

### 6- Sensitivity tests and spiking analyses with fluorescent cells

Spiking tests were performed to assess the performance of the ISET^®^ system for isolation and enrichment of fixed and live tumor cells. The sensitivity threshold, also named lower limit of detection (LLOD), is the lowest concentration of tumor cells detectable by a method. It is usually determined by extrapolating a plot of concentration (x) vs measurement unit (y) to the x-axis. The intercept is the lower limit of detection. Sensitivity is the smallest concentration change that a method is capable of detecting. It is determined from the slope of the previously described plot [39]. Since high sensitivity is critical in the rare cells field, we emphasized our work on determining sensitivity in a concentration range close to the LLOD.

Typically, 60–70% confluent cells were cultured and stained in flask with the addition of fluorescent dyes—either 5 μg/mL of Hoechst 33342 (Sigma-Aldrich, USA) or 5 μM of Cell Tracker^TM^ Orange (Life technologies, USA) overnight or for at least two hours. Cells in culture were rinsed twice with PBS to eliminate debris and detached from the flask by incubation with trypsin (0.125%) for 2 minutes at 37°C. Trypsin reaction was stopped by addition of 2 mL of culture media. Cells were further diluted with PBS, centrifuged at 300 g for 4 minutes, and carefully resuspended in 6 mL of culture media. 10 μL were then diluted 1:2 with Trypan Blue and cells were counted with a manual counting slide (KOVA Glasstic slide 10 with grids, Hycor Biomedical Inc, USA). Three or more counts were performed to obtain consistent counting values. If needed, the cell suspension was mixed again to obtain a homogenous cell dilution.

Fluorescent cells can also be resuspended with Rarecells^®^ Cytofix which allows to store them as fixed cells at +4°C for up to 5 days. The fluorescent staining can decrease or be lost in some cells after this time, implying that fluorescent cells must be counted again before use.

Counted fluorescent cultured cells were added, typically within 1 hour after trypsin treatment, to healthy donor blood. Very precise counting of cells used for spiking tests is critical, thus we used two alternative protocols (Fig 2).

1. Completely accurate counting by micropipetting individual fluorescent cells (1 to 10 cells) (Fig 2A and 2B). A 100 μL-drop of PBS containing approximately 10–40 cells was put in a Petri dish and observed under an inverted Olympus CK30-F2000 microscope at the x10 objective. Cells were carefully picked individually using a common micropipette (Gilson P2) and transferred one by one to the blood sample before its dilution with the Rarecells^®^ Buffer solution for fixed or live cells and filtration.
2. Precise counting (two serial counting) by fluorescent cells' dilution (30 to 300 cells) (Fig 2C). After a first accurate manual cell counting performed with Kova counting slides, a first dilution was prepared to reach the desired concentration. For instance, cells were carefully diluted in 10 mL of PBS in order to obtain the target number of cells (from 30 to 300) per 100 μL of solution. The cell count was again verified by depositing 100 μL of that dilution onto a petri dish and by visually identifying the cells using an inverted microscope. We then calculated the average number of cells per μL and this second count was used to determine the amount of diluted cells used in spiking tests. (We note that this type of further control is technically possible with Kova slides only for cell numbers from 100 to 300 per 100 μL). In all spiking experiments, a positive control with 5000-cultured fluorescent tumor cells added to 1 mL of blood was included to check for cell morphology integrity.

Blood samples with spiked-in tumor cells were then processed with one of the ISET^®^ filtration protocols as described above. For spiking tests with 10 mL of blood, we used a 150-mL Falcon-type tube (Dominique Dutscher, France) rather than two 50-mL Falcon-type tubes to dilute the blood (since 50-mL Falcon-type tubes are too small to accommodate 100 mL of diluted blood). After filtration, filters containing isolated fixed cells stained with Cell Tracker^TM^ Orange were optionally stained with 8 μg/mL of nuclear staining Hoechst for 10 min, rinsed with PBS and sterile distilled water and allowed to air dry protected from light. Optionally, filters were mounted between a slide and a coverslip using the mounting media ProLong^®^ Gold (Life Technologies, USA) before microscopic evaluation.

Fluorescent spiked tumor cells, isolated live and transferred to an 18 well-flat microchamber (Ibidi, Germany), or isolated fixed on ISET^®^ filters, were analyzed with an epi-fluorescence microscope (Zeiss, Germany). The DAPI filter (emission at 465 nm) and the TRITC filter (emission at 576 nm) were used for the detection of the Hoechst and the Cell Tracker^TM^ Orange signals, respectively. Criteria used to identify the spiked-in cells were: i- presence of a cell (defined as round shape with a visible cytoplasm and nucleus) by bright field examination of the filter and ii- detection of a genuine fluorescent signal with the TRITC filter. Presence of auto-fluorescent cell debris was excluded by analyzing the fluorescent image using the ‘FITC’ filter of the microscope. Images were processed with Image J software and reviewed independently by at least two operators.

For each series of tests, we measured the percentage of recovered cells, the percent coefficient of variation (%CV) and % error as follows:

$$\begin{array}{l} \%CV=\frac{s\tan dard\_deviation(measured\_data)}{average(measured\_data)},\\ \%Error=absolute\_value(100\%-average\_percentage\_of\_re\mathrm{cov}ered\_cells) \end{array}$$

### 7- Next generation sequencing (NGS) molecular characterization of single tumor cells and of cultured tumor cells after their isolation from blood by ISET^®^

#### NGS analyses of single tumor cells isolated by ISET^®^

Live or fixed tumor cells enriched from blood by ISET^®^, collected without sticking them to the filter as described in section 5, and transferred in suspension to a cell culture plate could be gently picked up manually and individually with a micropipette (Gilson P2) under an inverted Olympus CK30-F2000 microscope at the x10 objective and transferred to an individual 0.2 mL PCR tube.

Single cells’ proteins were lysed so as to release the DNA using our in house protocol (in 15 μL, 100 mM TrisHCl pH 8 and 400 μg/mL of proteinase K incubated for 2 hours at 60°C followed by proteinase K inactivation for 5 min at 94°C). The single-cell DNA was then pre-amplified by combining our lysis protocol and by adapting it to single-cell whole genome amplification (WGA) commercial kits: MALBAC (Yikon Genomics, China); Picoplex (Rubicon Genomics and New England BioLabs, USA); GenomePlex (Sigma-Aldrich, USA). Amplified DNA was then purified using the DNA Clean & Concentrator™-5 kit (Zymo Research, Germany) and quantified by Quant-iT™ PicoGreen^®^ dsDNA Assay Kit (Thermo Fisher Scientific, USA). The WGA products' quality was assessed by PCR as described elsewhere [40].

For NGS single-cell analysis using the Illumina approach, 250 ng of the amplified DNA was used to prepare libraries for high-throughput sequencing with the Nextera Exome Enrichment Kit (Illumina, USA) following the supplier’s protocol. A fraction of the library DNA from the pre-capture step was used for whole genome analysis. The libraries were sequenced on a HiSeq2000 (Illumina) in 2 x 100 bp paired-end runs. Data were mapped against the human genome and the number of reads per chromosome was calculated.

In order to perform single-cell targeted theranostic mutations' NGS analysis, we used the ThermoFisher approach, 10 ng of purified WGA DNA was used to generate 207 amplicons libraries including 50 oncogenes and tumor suppressor genes through the use of the optimized primer pool “Hotspot cancer panel v2” and Ion Torrent™ Library Kit V2 (ThermoFisher, USA), according to manufacturer’s recommendations. Briefly, Ion adapters and barcodes were added using the “bioanalyzer protocol” (for living single cells and bulk extracted DNA) or the “FFPE protocol” (for fixed single cells), respectively for 17 cycles and 20 cycles of pre-amplication. The quality of purified libraries was verified with the Bioanalyzer (Agilent, USA) and quantified by qPCR using the Ion Library TaqMan™ Quantitation Kit. Libraries were templated using the Ion One-Touch system and sequenced on Ion Torrent™ 318 chips using the Ion Personal Genome Machine™ (ThermoFisher, USA).

Data analysis was performed using the Ion Torrent™ suite (ThermoFisher, USA) and Excel (Microsoft, USA) softwares. Sequencing artifacts in homopolymer regions were filtered out. In theory, when analyzing diploid single cells, only three allele frequencies should be possible: 0% (wild-type), 50% (heterozygous mutant/wild type) and 100% (homozygous mutant). However, WGA and sequencing methods introduce variability and quantitative amplification biases. In consequence, COSMIC hotspot mutations were included in our analysis only if present in at least one single cell WGA sample with a quality score over 100 and 15% allele frequency while using 10X depth amplicon coverage. Venn diagrams were drawn using a public online tool (bioinformatics.psb.ugent.be/webtools/Venn/).

#### NGS analysis of tumor cells isolated by ISET^®^ and cultured for 72 hours

In order to investigate the feasibility of studying possible tumor cells' genetic changes induced by their isolation from blood with our method and culture for 72 hours, we performed NGS analysis on tumor cells before and after isolation by ISET^®^ and culture. Approximately 10^4^ live A549 and 10^4^ HCT116 live tumor cells were seeded in a 24-wells culture plate before and after their enrichment from blood by ISET^®^, as described above. Cells were kept in culture for 72h before their collection for DNA extraction by incubating them for 2 minutes with trypsin (0.125%) at 37°C. Trypsin reaction was stopped by addition of 2 mL of complete media. Cells were further diluted with PBS, centrifuged at 300 g for 4 minutes, and carefully resuspended in 200 μL of PBS for DNA extraction.

We extracted bulk DNA from our samples using the QIAamp DNA Blood Mini Kit (Qiagen, Germany) following the manufacturer's instructions. We then used 10 ng of extracted bulk DNA from unfiltered control tumor cells and from tumor cells cultured for 72 hours after ISET^®^ filtration using the same ThermoFisher protocols with the ‘Hotspot Cancer Panel V2’ and Ion Torrent™ Library Kit V2 (ThermoFisher, USA) used for single tumor cells.

Data from experiments on tumor cells' populations were further processed with Sophia DDM^®^ software (Sophia Genetics, Switzerland) to calculate variant fraction and coverage. Only variants with more than 5% of allele frequency were reported.

## Results

### A- Cell size analysis

**Fig 3:**
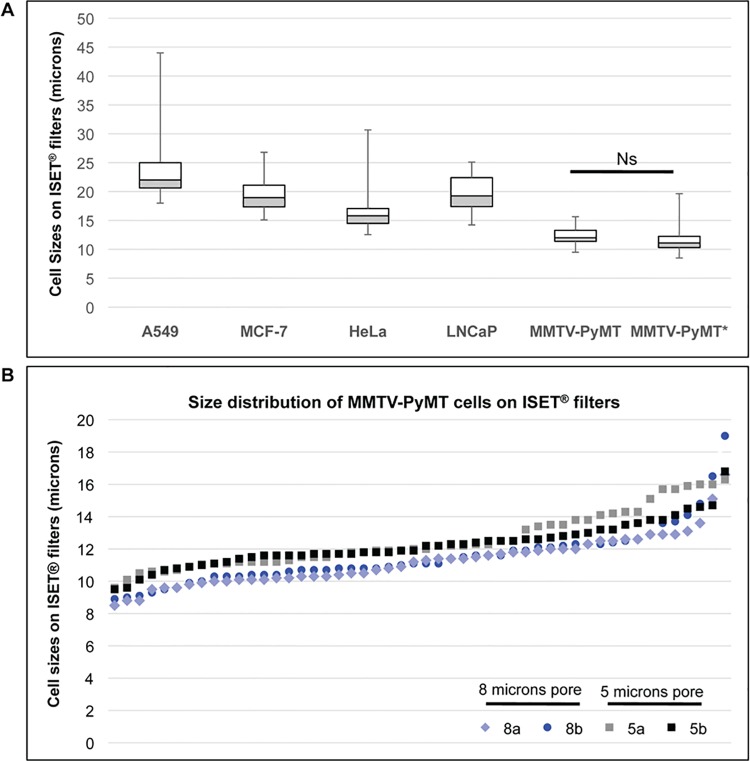
Cell size assessment of tumor cells from human and mouse tumor cell lines. (A) Box plot of cell sizes (diameters) according to each cancer cell line tested. Each box has its ends at the quartiles, and the median of distribution is marked by a line within the box. Error bars indicates maximum and minimum diameters. Cells from human and mouse tumor cell lines were incubated 3 min with the Rarecells^®^ Buffer without blood and recovered on standard (8 micron-pore) ISET^®^ filters. MMTV-PyMT*: values measured for MMTV-PyMT cells isolated using 5 micron-pores ISET^®^ filters. Cells were stained on filters using May-Grünwald Giemsa before analysis by microscopic observation. Ns: Not significant. (B) Size distribution of two series of 50 MMTV-PyMT cells on ISET^®^ filters isolated using 8 micron-pore filters (8a and 8b) and 5 micron-pores filters (5a and 5b).

A comparative cell size analysis of cells from human and mouse tumor cell lines was performed to characterize cells used to assess the sensitivity of tumor cells isolation by ISET^**®**^ (Fig 3A and Table A in S1 File). As detailed in the Methods section 2, at least 100 individual cells from each cell line were analyzed after filtration through standard 8 micron-pores ISET^®^ filters, and MGG staining. The median diameter of cancer cells ranged from 12 μm (mouse MMTV-PyMT) to 22 μm (human A549). The mean size ranged from 11.4 μm (MMTV-PyMT, standard deviation [stdev]: 1.6) to 23.2 μm (A549, stdev: 4.4) (Table A in S1 File). LNCaP median diameter measured 19.2 μm which is consistent with previously reported data [10].

We also studied the smallest MMTV-PyMT cells by comparing the range of cell sizes after filtration with the standard 8 micron-pore ISET^®^ filters with those obtained after filtration with 5 micron-pore ISET^®^ filters, in order to assess a possible loss of small cells when using the 8 micron-pore filters. The median and mean diameters of MMTV-PyMT on 5 micron-pore filters were respectively 11.1 μm and 11.4 μm (stdev: 1.7) (Fig 3 and Table A in S1 File). Using the chi^2^ test, when comparing the 100 individual data points obtained on the 8 micron-pore and the 5 micron-pore filters, we did not observe a statistical difference. Furthermore, when plotting cell size data for two series of 50 MMTV-PyMT cells for each 8 and 5 micron-pore filters, we observed a similar distribution throughout all the size ranges (i.e. 8.5 to 16.8 μm) for both types of filters (Fig 3B), indicating that there is no size-selection bias when isolating the small cells from this cell line with the standard pore size of ISET^®^ filters. Fig 3A in fact shows that the MMTV-PyMT median cell size after ISET^®^ filtration through 5-micron pores is slightly greater than that calculated after ISET^®^ filtration through 8-micron pores. However, the difference is not statistically significant and could be related to different batches of cells collected at different times.

These data are consistent with the results described in section C-1 (Table C in S1 File) showing that the size of live MMTV-PyMT cells remains essentially the same before and after ISET^®^ filtration.

### B- *In vitro* performance of the ISET^®^ system for isolation of fixed tumor cells

#### B1. Sensitivity and precision (repeatability and reproducibility) of the ISET^®^ system for isolation of fixed tumor cells from blood

We assessed the analytical sensitivity, sensitivity threshold (or Lower Limit of Detection (LLOD), i.e. the smallest amount of cancer cells which can be detected), repeatability and reproducibility of the ISET^®^ standard system for isolation of fixed tumor cells (Fig 1A). Because of the particular clinical relevance of detecting tumor cells in blood when they are very rare, we tested the LLOD and the sensitivity at the LLOD of the ISET^®^ system by performing analyses with very low numbers of fluorescent tumor cells, counted one by one by micropipetting and spiked in blood from healthy donors. Typical "spiking tests" experiments were performed by adding individually micropipetted A549, MCF-7, HeLa or MMTV-PyMT fluorescent tumor cells to one to ten mL of blood and processing the blood by ISET^®^ filtration (Fig 2A). We then analyzed the filter and detected the recovered spiked cells by looking for their fluorescent signal. We only counted fluorescent signals proven to be fluorescent cells (see Methods, section 6). This *in vitro* assay was 100% specific (n = 4 tests with 0 spiked cells in 1 mL of blood).

To assess reproducibility by two different operators using different reagent lots and instruments, we performed 12-fold replicate tests with two A549 cells spiked in one mL of blood. We found a total number of 21 and 20 out of 24 spiked cells per operator, respectively (Table 1), with an average recovery success rate of 88% and 83%

**Table 1:** *In vitro* assay of the repeatability and reproducibility of ISET^®^ sensitivity tests for isolation of fixed cells.

	Operator A	Operator B
Blood processed	1 mL	1 mL
**Spiked tumor cells/test**	2	2
**Fixed tumor cells detected by ISET^®^ per test (N = 12 tests)**	1	2
	2	2
	2	2
	1	1
	2	1
	2	2
	2	2
	2	2
	1	2
	2	0
	2	2
	2	2
**Total detected/spiked cells**	21/24	20/24
**Recovery success rate**	88%	83%

Given the structure of the ISET^®^ cartridge (which separates 10 mL of blood in 10 samples of one mL of whole blood i.e. 10 samples of 10 mL of diluted blood) and since the LLOD of one tumor cell per mL of blood had been found by our team and by independent teams [12, 25, 27–29], we thought that the LLOD and physical limit of the ISET^®^ system down to one tumor cell per 10 mL of blood could potentially be achieved and should be tested. Consequently we spiked by individual cell counting in 6-fold replicate tests two A549 cells in one mL, five mL and ten mL of blood. We found a total of 10 of 12, 10 of 12 and 12 of 12 spiked cells, respectively, with an average recovery success rate of 83%, 83%, and 100% respectively (Table 2). Similarly, in 10-fold replicate tests with 1 MCF-7, we recovered 9 out 10 spiked MCF-7 cells. In 3-fold replicate tests with 1 and 3 HeLa cells, we recovered 3 out 3 spiked HeLa cells, and 9 out of 9 spiked HeLa cells, respectively (Table 3).

**Table 2:** *In vitro* assay of ISET^®^ sensitivity threshold for isolation of fixed A549 cells.

Blood processed	1 mL	5 mL	10 mL
**Spiked tumor cells/test**	2	2	2
**Fixed tumor cells detected by ISET**^**®**^ **per test (N = 6 tests)**	1	1	2
	2	2	2
	2	2	2
	1	2	2
	2	2	2
	2	1	2
**Total detected/spiked cells**	10/12	10/12	12/12
**Recovery success rate**	83%	83%	100%

**Table 3:** *In vitro* assay of ISET^®^ sensitivity using various types of cancer cells.

Cell line	MCF-7		HeLa		MMTV-PyMT
Blood processed	1 mL		1 mL		1 mL
**Spiked tumor cells/test**	1		1	3	2
**Fixed tumor cells detected by ISET^®^ per test (N = 3–5 tests)**	1	1	1	3	2
	1	0	1	3	2
	1	1	1	3	1
	1	1			1
	1	1			
**Total detected/spiked cells**	9/10		3/3	9/9	6/8
**Recovery success rate**	90%		100%	100%	75%

Using the same protocol and workflow, we also spiked the mouse MMTV-PyMT cells which isolation had been studied on 8 micron-pore and 5 micron-pore filters (Fig 3B). In 4-fold replicate tests with two MMTV-PyMT cells, we found a total number of 6 out of 8 spiked cells (Table 3).

Our sensitivity tests with the ISET^®^ System were found to be reproducible and the LLOD was determined as one cancer cell per 10 mL of blood (Tables 1–3). Sensitivity at the closest concentration to the LLOD was 100%. Importantly, similar average recovery success rates were found with various volumes of blood processed (1 mL, 5 mL or 10 mL of blood) (Table 2) and tumor cells having different sizes (median sizes ranging from 12 to 22 μm) (Tables 1–3, A in S1 File and Fig 3).

#### B2. Linearity and accuracy of the ISET^®^ System for isolation of fixed tumor cells from blood

We next assessed the ISET^®^ System's linearity (i.e. predictability of sample recovery with known dilution factors within an assay range or overall sensitivity) and accuracy (i.e. trueness as compared to a reference) by using fluorescent A549 cells carefully counted by dilution (see Methods, section 6) and processed with the ISET^®^ fixed cells protocol (Fig 1A and Methods, section 3). Recovery is calculated when cells are spiked into the relevant matrix, blood in our case, at a minimum of five concentration levels covering the linear range of the assay (according to the FDA definition, Docket No. 2004D-0163).

**Fig 4:**
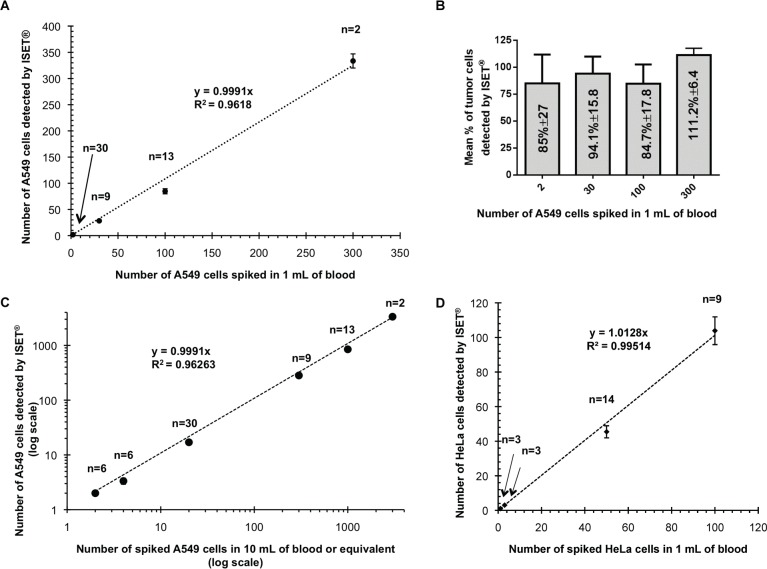
*In vitro* assay of ISET^®^ linearity. (A) Number of tumor cells (A549) detected on ISET^®^ filters plotted against expected number of tumor cells. Cells were spiked into whole blood after counting by dilution (30 to 300 cells) or counting by individual cells micromanipulation (2 cells). 0 cells (n = 4 replicates), 2 cells (n = 30), 30 cells (n = 9), 100 cells (n = 13) or 300 A549 cells (n = 2) were added to 1 mL of blood. Error bars (when visible) indicate standard error. (B) Mean % of tumor cells (A549) detected on ISET^®^ filters plotted against expected number of tumor cells spiked into whole blood. (C) Number of tumor cells (A549) observed on ISET^®^ filters plotted against expected number of tumor cells after extrapolation to 10 mL of blood (log scale). In addition to the test with 2 cells spiked in 10 mL (n = 6 replicates), to facilitate comparison with different volumes of blood, tests in 1 mL or 5 mL were plotted as their equivalent in 10 mL (See Methods). Error bars (when visible) indicate standard error. (D) Number of tumor cells (HeLa) detected on ISET^®^ filters plotted against expected number of tumor cells. Cells were added into the whole blood after their counting by dilution (50 to 100 cells) or after their counting by individual cells micromanipulation (1 or 3 cells). 1 cell (n = 3 replicates), 3 cells (n = 3), 50 cells (n = 14) or 100 cells (n = 9) were added to 1 mL of blood (See Methods). Error bars (when visible) indicate standard error.

We have assessed linearity by spiking 30, 100 and 300 A549 cells, all counted by dilution, in one mL of blood in 9, 13 and 2 replicates, respectively. We have also plotted the number of observed fluorescent A549 cells on ISET^®^ filters after their counting by micropipetting versus the expected number of A549 (Tables 1 and 2 and Fig 4A). Using a linear regression analysis, we found a slope of 0.9991 and correlation coefficient (R^2^) of 0.96 (Fig 4A and Table B in S1 File**).** After addition of a number of 2, 30, 100 and 300 cells in 1 mL of blood, we recovered an average number of 1.7 cells per mL (85%, n = 30 replicates), 28 cells per mL (94%, n = 9), 85 cells per mL (85%, n = 13) and 334 cells per mL (111%, n = 2), respectively (Fig 4B and Table B in S1 File). We also obtained similar cell recovery rates by spiking dilutions of 50 and 100 HeLa cells in 1 mL of blood (respectively, 91% n = 14 and 104% n = 9) and 50 MCF-7 cells in 5 mL of blood (105%, n = 5) (S1A Fig).

Since the ISET^®^ cartridge partitions 10 mL of blood in 10 times 1 mL volumes, cell recovery in 10 mL of blood has the same efficiency as cell recovery in 1 mL of blood (also demonstrated by our tests (Table 2)). We have thus plotted the equivalent tests in 10 mL of blood (i.e. 2 cells in 1 mL equivalent to 20 cells in 10 mL) and calculated linearity over 6 concentrations of A549 tumor cells ranging from 2 to 3000 CTC in 10 mL equivalent of blood. In this analysis, our average recovery rate (overall sensitivity) was 99.91% (Fig 4C). We observed similar linearity (overall sensitivity) with HeLa cells over 4 tumor cell concentrations (Fig 4D), with an average recovery rate of 100% ± 1%.

Finally, we calculated the percent coefficient of variation (% CV), a measure of precision, for each condition tested (cell line, volume of blood, number of spiked cells) (Table B in S1 File). Average %CV was 22% in experiments with spiked cells counted by cell dilution (n = 6 conditions, 52 tests). In experiments with micromanipulated spiked cells, average %CV was also 22% (n = 9 conditions, 62 tests) (Table B in S1 File). As expected, in these tests with an extremely low number of spiked cells, which assess the detection limit, the %CV values are usually higher because of the binary nature of the test results (0 or 1 cell) which has an impact on the readout. Accuracy (measured as recovery error) was below 25% for all conditions (Table B in S1 File). Under the rigorous conditions of our spiking tests and readings (see Methods), tumor cells' recovery obtained using spiked cells counted by dilution was consistent with that obtained using spiked cells counted by micropipetting.

It is worth mentioning that cell spiking with the individually micropipetted cells protocol requires technical skills in single cells' micromanipulation and is a particularly demanding test. One single micropipetted cell added to ten mL of blood (containing on average 50 to 100 million leukocytes and 50 billion erythrocytes) can be lost if it is damaged by the trypsin used to detach cells from the petri dish. In this case, the spiked rare cells can be lost independently from the capability of the ISET^®^ system to capture them from blood.

**Fig 5:**
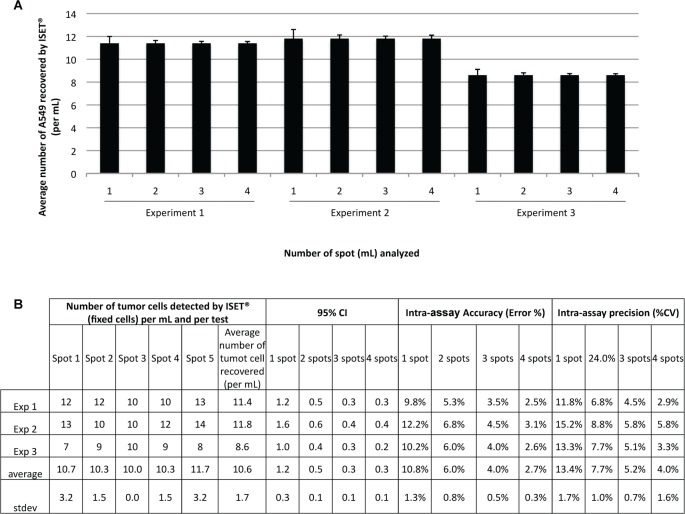
Assessment of ISET^®^ intra-assay accuracy and precision. 50 A549 cells were spiked into 5 mL of blood (n = 3 experiments, 43 to 49 cells per 5 mL). The number of tumor cells found on each spot after ISET^®^ filtration (each corresponding to the filtration of 1 mL of blood) was recorded. Experiments were done on 5 spots but for intra-assay precision and accuracy only the assessment of the number of cells found on single spots or randomly grouped spots (any 1, any 2, any 3, any 4 spots) is relevant. The cell counting on the combination of all the 5 spots was used as reference. Results show that cell counting on four spots exhibited a representative mean tumor cells value per mL of blood. (A) Bar chart with the mean tumor cell number per spot and corresponding standard error of the mean (error bars) depending on the number of spots analyzed. Error bars are calculated using the standard deviation in different combinations of any 4 spots, any 3 spots, any 2 spots or any 1 spot, respectively. If only one spot is considered, standard deviation is higher than when counting 4 spots, showing that counting on four spots gives a reliable mean tumor cells' value per mL of blood. (B) Table indicating the number of tumor cells found on each spot for each of the five experiments, the 95% confidence interval (CI), the precision (%CV) and the accuracy (%error) depending on the number of spots analyzed (1 to 4) as compared to the analysis on five spots.

To verify if the cells are evenly distributed over 5 spots, we used the methodology described by Krebs *et al*. [12] and assessed intra-assay precision and accuracy (Fig 5). We examined the number of tumor cells found on each spot after spiking of 50 A549 cells counted by dilution into 5 mL of blood and filtration on 5 spots (each corresponding to the filtration of 1 mL of blood). To study the impact of tumor cells' distribution on 5 spots, we diluted tumor cells and obtained 43 to 59 cells per 5 mL in three different experiments (on average 8.6 to 11.8 A549 per mL). For each experiment, tumor cells' counts were calculated from all possible combinations of spots (i.e. any 1, any 2, any 3, any 4). The average tumor cells' number (± Standard Error) with 95% confidence intervals (CI) was calculated for each experiment and each combination of spots as described by Krebs *et al*. [12]. In an experiment with on average 8.6 tumor cells per mL, analyzing four spots provided an accurate estimation of the true mean (mean ± SE = 8.6 ± 0.13 tumor cells per mL, 95% CI: 8.35–8.85). When analyzing 3 spots or 2 spots, the estimation was less accurate, respectively 8.6 ± 0.14 (CI: 8.33–8.87) and 8.6 ± 0.21 (CI: 8.19–9.01) tumor cells per mL. Similarly, in an experiment with on average 11.8 tumor cells per mL, analyzing four spots provided an accurate estimation of the true mean (11.8 ± 0.31, 95% CI: 11.41–12.19). When analyzing 3 spots or 2 spots, the estimation was less accurate, respectively 11.8 ± 0.22 (CI: 11.37–12.23) and 11.8 ± 0.33 (CI: 11.16–12.44) (Fig 5).

Altogether, ISET^®^ intra-assay average precision was 4% (range 2.9% to 5.8%) and average accuracy was 2.7% (range 2.5% to 3.1%) when tumor cells were enumerated on 4 spots. Enumerating tumor cells on 3, 2 and 1 spot(s) leads to a decreased average precision (respectively, 5.2%, 7.7% and 13.4%) and a decreased average accuracy (respectively, 4%, 6% and 10.8%) (Fig 5). We thus found that the ISET^®^ System's intra-assay precision and accuracy related to individual spot’s cell counting is consistent when 50 cells are filtered on a total of 5 spots and cells are enumerated on at least 4 spots. We have also performed further analyses showing that intra-assay precision and accuracy is maintained with an extended cell range of 29 to 70 cells in 5 mL of blood (6 to 14 cells per mL) (S2 Fig). These results show that, if the number of tumor cells in 5 mL of blood is comprised between 29 and 70 (between 58 and 140 in 10 mL of blood), counting the tumor cells on 4 spots gives the same number of tumor cells per mL of blood as counting the tumor cells in 5 or 10 mL of blood.

Overall, we found that the ISET^®^ filtration's accuracy, precision and linearity are very high. Furthermore, the average recovery rate from two up to 3000 tumor cells per 10 mL (or equivalent) of blood is close to 100%.

#### B3. Sensitivity of the ISET^®^ system for isolation of fixed tumor cells from blood after collection of plasma

Since detection of rare circulating tumor cells logically prefers methods using a non-negligible volume of blood (8 to 10 mL), we checked the feasibility of isolating plasma and tumor cells from the same blood sample. This protocol aims to perform multiple tests using the same sample, in order to make full use of the precious patient’s blood sample (Fig 1B). We assessed the *in vitro* sensitivity of this protocol by spiking 10 fluorescent A549 cells, counted one by one by micropipetting, in 5 mL of healthy donor's blood. In 4-fold replicate tests, we found 9 cells twice, 10 cells once and 7 cells once (Table 4) with a recovery success rate of 88%. The morphology of the spiked cells was intact as we found by performing MGG cytological staining (S1D Fig). The very high recovery success rate demonstrates that the LLOD and overall sensitivity for dual collection of plasma and tumor cells using the ISET^®^ system are similar to that of the standard ISET^®^ protocol.

**Table 4:** *In vitro* assay of ISET^®^ sensitivity with dual collection of tumor cells and plasma.

Blood processed	5 mL
**Spiked tumor cells/test**	10
**Fixed tumor cells detected by ISET^®^ per test (N = 4 tests)**	10
	9
	7
	9
**Total detected/spiked cells**	35/40
**Recovery success rate**	88%

#### B4. Assessment of the number of contaminant leucocytes on filters after isolation of fixed tumor cells

The ISET^®^ system successfully eliminates all red blood cells and the majority of white blood cells, however some of them remain. By using cytopathological staining, we counted the number of residual leucocytes on the filters obtained by filtrating the blood of 4 different healthy donors (see Methods, section 3). With the standard ISET^®^ workflow for isolation of fixed cells, the number of leucocytes on the filter is variable, ranging from 1208 to 3188 cells per mL (1779 on average). This corresponds to an approximate enrichment factor of 4 logs, in agreement with the data reported by other teams [12].

### C- *In vitro* performance of the ISET^®^ system for enrichment of live tumor cells from blood

We then assessed the ISET^®^ system's sensitivity parameters for enrichment of live tumor cells which are relevant for research studies and in particular for molecular characterization of tumor cells, and their culture.

#### C1 Sensitivity and reproducibility of the ISET^®^ system for enrichment of live tumor cells

Isolating by filtration live cells with no antibody-related bias and no or minimum cell loss is a very challenging goal as live tumor cells are flexible and can more easily slip through the pores. We have concentrated our efforts on developing a new filtration buffer and a variant ISET^®^ protocol allowing enrichment of live tumor cells and their recovery in suspension for molecular analysis, in particular transcripts' analyses, and cell culture (Fig 1C). We have then assessed the *in vitro* sensitivity of this new workflow by spiking various numbers of cultured live A549 and LNCaP fluorescent tumor cells, collected one by one by micropipetting, in 1 mL of healthy donor's blood and performing the new protocol. The enriched cells have been transferred to an Ibidi microchamber and identified under microscope due to their fluorescent signal (Fig 2B), as described in the Methods, section 6.

In 6-fold replicate tests using 1, 3, 5 and 10 spiked live fluorescent A549 cells in 1 mL of blood, we found 6/6, 16/18, 24/30 and 50/60 cells (Table 5), with an overall recovery success rate of 100%, 89%, 80% and 83%, respectively. Spiking tests were repeated with 5-fold replicate tests using 1 and 5 spiked live fluorescent LNCaP cells in 1 mL of blood. We found a total number of 4/5 and 21/25 cells with an overall recovery success rate of 80 and 84%, respectively (Table 5). Furthermore, using a Trypan blue stain, we found that the viability of the enriched live tumor cells was maintained. The recovery rate and the analytical sensitivity we obtained with live A549 and LNCaP cells are very high. Sensitivity at the closest concentration to the LLOD tested (i.e. 1 tumor cell in 1 mL of blood) was 100% and similar to that obtained with the standard ISET^®^ protocol for isolation of fixed cells (Tables 1–3).

**Table 5:** *In vitro* assay of ISET^®^ sensitivity for enrichment of live tumor cells.

Cell line	A549				LNCaP	
Blood processed	1 mL				1 mL	
**Spiked tumor cells/test**	1	3	5	10	1	5
**Live tumor cells detected by ISET^®^ per test (N = 5–6 tests)**	1	3	4	9	1	4
	1	3	5	6	1	4
	1	2	4	8	0	5
	1	3	4	9	1	3
	1	2	3	10	1	5
	1	3	4	8		
**Total detected/spiked cells**	6/6	16/18	24/30	50/60	4/5	21/25
**Recovery success rate**	100%	89%	80%	83%	80%	84%

#### C2 Assessment of cell size and viability before and after enrichment of live tumor cells with the ISET^®^ system

**Fig 6:**
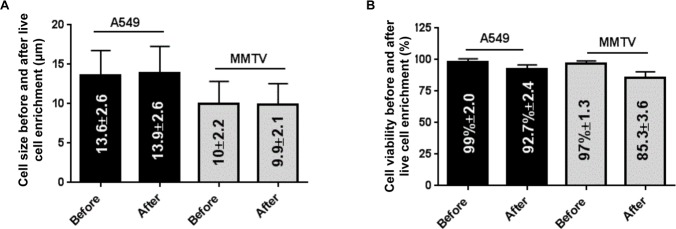
Cell sizes and viability before and after live cell enrichment. Cell sizes (median diameter) (A) or viability (B) according to each cancer cell line tested before or after live cell enrichment (n = 3 experiments). Error bars indicate standard error. Cells from human and mouse tumor cell lines were incubated 5 min with the Rarecells^®^ Live Cells Buffer with blood and recovered on standard (8 micron-pore) ISET^®^ filters. MMTV = MMTV-PyMT. Cell size and viability were analyzed using the TC20™ Automated Cell Counter (Bio Rad) and Trypan Blue stain.

The precise size of at least 200 live A549 and MMTV-PyMT cells has been carefully calculated before and after filtration through 8-micron pores (Table C in S1 File and Fig 6). The median diameter remained essentially the same before and after filtration of live cells from both cell lines (13.6 μm ± 2.6 and 13.9 μm ± 2.6 for A549 cells and 10.0 μm ± 2.2 and 9.9 μm ± 2.2 for MMTV-PyMT cells, n = 3 experiments, respectively). Results demonstrate that filtration does not induce a selection in the cell size, including for the smallest cells (MMTV-PyMT) when they are kept alive (Fig 6). By using Trypan Blue exclusion, only the size of living cells has been taken into account. We have also calculated the rate of viability of live cells before and after filtration and found 99% ± 2% and 92.7% ± 2.4% for A549 cells and 97% ± 1.3 and 85% ± 3.4% for MMTV-PyMT cells, respectively (Fig 6 and Table C in S1 File).

#### C3 Assessment of cell surface marker expression before and after enrichment of live tumor cells with the ISET^®^ system

**Fig 7:**
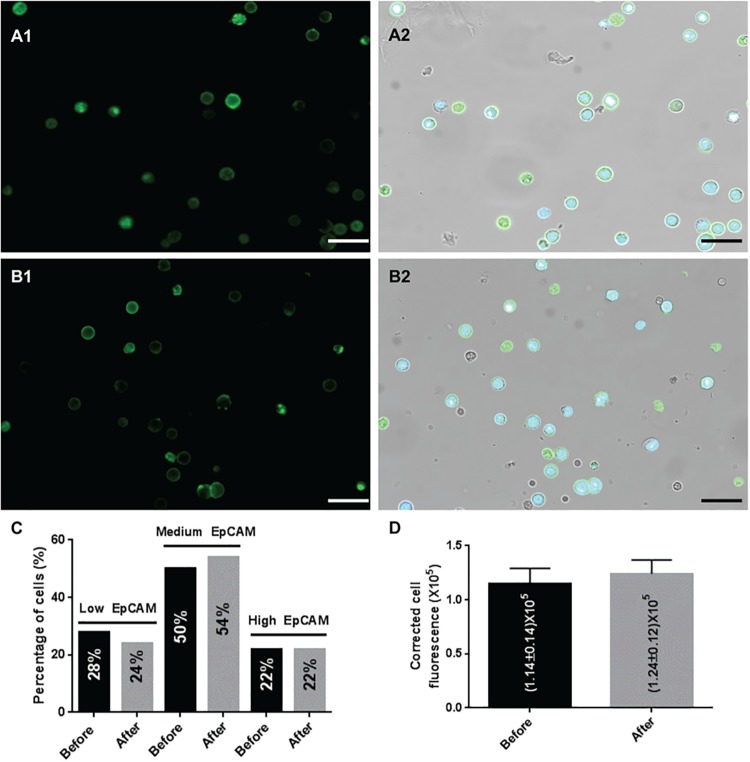
Comparison of EpCAM fluorescence on labeled cells before and after live cell enrichment by ISET^®^. Representative images of MCF-7 cells before live cell enrichment showing EpCAM fluorescence alone (A1) and merged Hoechst and EpCAM fluorescence with bright field image (A2) are each presented with a scale bar of 50 microns. Representative images of MCF-7 cells after live cell enrichment showing EpCAM fluorescence alone (B1) and merged Hoechst and EpCAM fluorescence with bright field image (B2) are each presented with a scale bar of 50 microns. (C) Comparison of cell distribution across three levels of EpCAM expression for 50 MCF-7 cells before and 50 MCF-7 cells after live cell enrichment. The low EpCAM group comprises cells with corrected total cell fluorescence (CTCF) below 91000 units, the medium EpCAM category regroups cells with CTFC between 91000 and 200000 units and the high EpCAM group contains cells with CTCF above 200000 units. (D) Comparison of median corrected total cell fluorescence (CTCF) calculated on 50 MCF-7 cells before and 50 MCF-7 cells after live cell enrichment. Error bars indicate standard error.

As changes in protein expression could potentially occur during filtration, we also tested whether filtration of live tumor cells with our protocol induces a change in antigen expression by using EpCAM antibody immunofluorescence and labeling live MCF-7 cells before and after filtration. The result of our analysis is shown in Fig 7. We did not find any difference in EpCAM expression induced by filtration under the tested filtration conditions. In fact, quantitative analysis of fluorescence using ImageJ (Fig 7D) revealed that the median corrected total cell fluorescence (CTCF) calculated on 50 MCF-7 cells was very similar before (median of 114665 ± 14363 with CTCF ranging from 43754 to 494919) and after (median of 124021 ± 12697 with CTCF ranging from 65799 to 594996 units) live cell enrichment. Furthermore, when applying cutoffs of CTCF to distinguish cells with low EpCAM expression from those with medium and high EpCAM expression we found very similar distributions of cells across all three categories: 28% (low), 50% (medium), 22% (high) before live cell enrichment and 24% (low), 54% (medium), 22% (high) after live cell enrichment, respectively (Fig 7C).

#### C4 *In vitro* culture of live tumor cells enriched from blood with the ISET^®^ system

**Fig 8:**
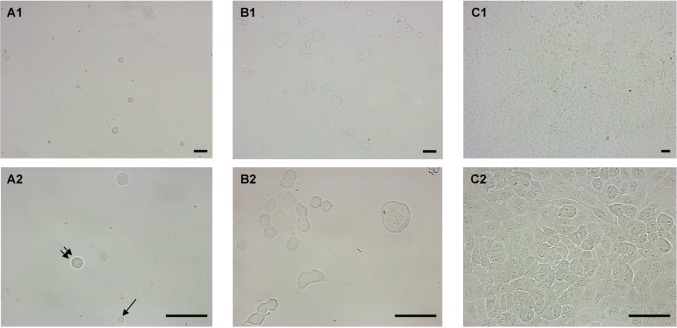
*In vitro* culture of A549 tumor cells after enrichment from blood using ISET^®^. Representative bright field images of A549 cells after live cell enrichment from blood and *in vitro* culture were obtained at 3 distinct time points. Scale bar of 50 microns. (A1, A2) Images at day zero (D0 = 2h of culture) showing A549 cells after live cell enrichment from blood obtained using the 10X (A1) and the 40X objective (A2). At 40X (A2) a single normal leucocyte is pointed by a single black arrow and a representative tumor cell is pointed by two arrows. (B1, B2) Images at day 2 (D2 = 2 days of culture) showing A549 tumor cells growing after live cell enrichment from blood obtained using the 10X (B1) and the 40X objective (B2). (C1, C2) Images at day 5 (D5 = 5 days of culture) showing A549 tumor cells growing after live cell enrichment from blood obtained using the 10X (C1) and the 40X objective (C2).

We tested if cells from cell lines could survive and proliferate *in vitro* after their enrichment from blood using the ISET^®^ System. We spiked A549 cells in blood in duplicate tests and processed the samples with the live cell enrichment protocol as described in the Methods section. We then compared imaging results at day zero (D0) with those obtained after 2 (D2) and 5 days (D5) of *in vitro* culture of enriched A549 cells isolated from blood (Fig 8). In both replicate tests, tumor cells observed at D0 were scattered as individual cells across the surface of the round coverslips (1.2 cm in diameter) used for their culture with an overall similar yet heterogeneous cell population density (Fig 8A). Tumor cells observed at D2 were mainly present as small clusters with a higher overall cell population density and some mitotic cells, clearly indicating cell proliferation (Fig 8B). Tumor cells observed at D5 were present exclusively as large clusters or colonies of proliferating cells with higher density, nearly covering the whole surface of the coverslips (Fig 8C). Interestingly, very few residual leucocytes were found at D0 after the first washing step and none of them were observed at D2 and D5, suggesting that stringent *in vitro* culture conditions can potentially select pure tumor cells populations. These results show that tumor cells' growth in culture is possible after live tumor cells enrichment by the ISET^®^ System.

#### C5 Assessment of cytoskeleton markers before and after enrichment of live tumor cells with the ISET^®^ system and short term *in vitro* culture

**Fig 9:**
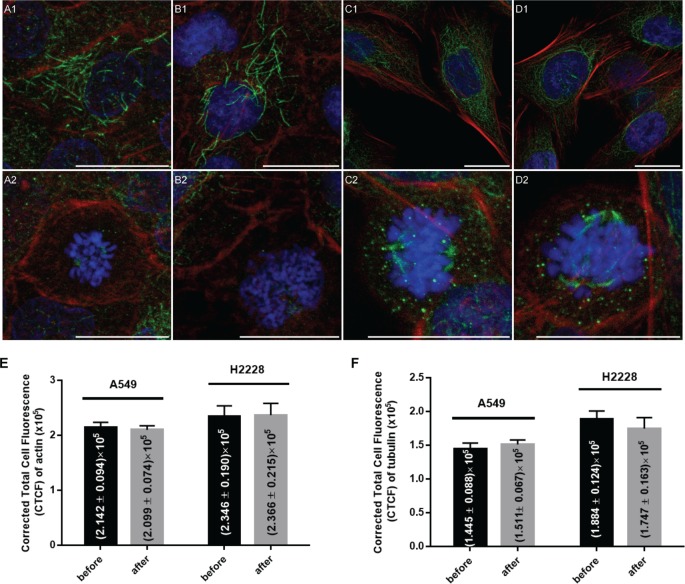
Cytoskeleton analysis of A549 and H2228 cells before and after live cell enrichment by ISET^®^ and *in vitro* culture. Representative images of A549 and H2228 cells showing merged Hoechst (blue), actin (red) and tubulin [41] fluorescence were taken after 72h of *in vitro* culture using the 63X objective and are each presented with a scale bar of 20 microns. Images of A549 cells before filtration show examples of cells in G1/S phase (A1) and during mitosis (A2) for comparison with images showing A549 cells after filtration in G1/S phase (B1) and during mitosis (B2). Images of H2228 cells before filtration show examples of cells in G1/S phase (C1) and during mitosis (C2) for comparison with images showing H2228 cells after filtration in G1/S phase (D1) and during mitosis (D2). Comparisons of median corrected total cell fluorescence (CTCF) of actin (E) and of tubulin (F) calculated on 30 A549 and 30 H2228 cells before and 30 A549 and 30 H2228 cells after live cell enrichment. Error bars indicate standard error.

In order to determine the potential impact of filtration on the cytoskeleton of live tumor cells, we performed immunofluorescence staining of actin microfilaments and microtubules of A549 and H2228 cells. We compare the profile of cells before enrichment to the one after their enrichment with the ISET^®^ System and short term *in vitro* culture (for 72 hours). For both cell types, key features of cytoskeleton dynamics involved in crucial cellular processes such as proliferation were observed before and after live tumor cells isolation using ISET^®^. Results are shown in Fig 9.

Microscopy analysis revealed very similar actin and tubulin structures when comparing untreated A549 cells (Fig 9A) to A549 cells enriched live using the ISET^®^ system (Fig 9B) and kept 72h in culture. Importantly, no significant difference was observed in spindle morphogenesis during mitosis when comparing A549 cells before (Fig 9A2) and after live cell enrichment with ISET^®^ (Fig 9B2). In addition, all A549 cells observed at G1/S phase (Fig 9A1 and 9B1) displayed polygonal global shape, uneven distribution and unaligned orientations of F-actin filaments as well as frequent weakening of the peripheral actin cortex colocalized with microtubule-enriched plasma membrane extensions. These observations are consistent with previous literature on A549 cells [42] and correlate with known invasive properties [43] and stiffness' plasticity [44] of A549 cells which are prone to EMT transformation. Similarly, enrichment followed by 72 hours of *in vitro* culture had no visible impact on the cytoskeleton of H2228 cells (Fig 9D) as compared to untreated H2228 cells (Fig 9C). At G1/S phase, scattered microtubules and long straight actin filaments were observed with thicker actin bundles present near attachment sites of the plasma membrane for H2228 cells before (Fig 9C1) and after live cell enrichment (Fig 9D1). Enriched H2228 cells entering mitosis (Fig 9D2) displayed mitotic rounding, chromatin condensation and spindle morphogenesis with no apparent alteration compared to untreated controls (Fig 9C2).

Furthermore, global quantitative analysis of actin fluorescence (Fig 9E) performed on 30 randomly picked cells revealed very similar results of median CTCF before (median of 214166 ± 9416 for A549 and 234552 ± 19036 for H2228) and after live cell enrichment and culture (median of 209915 ± 7405 for A549 and 236599 ± 21468 for H2228). Using a Student's t-test, the differences of actin CTCF before and after live cell enrichment were found to be not significant (p value of Student test = 0.997 for A549 and 0.912 for H228).

Similarly, global quantitative analysis of tubulin fluorescence (Fig 9F) performed on 30 randomly picked cells also revealed very similar results of median CTCF before (median of 144482 ± 8824 for A549 and 188390 ± 12406 for H2228) and after live cell enrichment and culture (median of 151118 ± 6732 for A549 and 174672 ± 16327 for H2228). Using a Student test, the differences of tubulin CTCF before and after live cell enrichment were found to be not significant (p value of Student test = 0.296 for A549 and 0.916 for H2228).

Of note, median CTCF was higher for H2228 as compared to A549, which could be due to the larger size of H2228 cells or a higher expression level of tubulin and actin.

Altogether these results indicate that tumor cells' morphology and cellular functions related to the cytoskeleton distribution of F-actin and acetyl-α-tubulin can be either conserved or restored after 72h of *in vitro* culture following live tumor cells isolation from blood using the ISET^®^ system and protocol.

#### C6 Estimation of the remaining leukocytes after live tumor cells filtration and their depletion using CD45 magnetic beads

The ISET^®^ System workflow for live cells successfully eliminates all red blood cells and the majority of white blood cells. The remaining white blood cells (WBC) were counted in 2 tests as described in the Methods section 5. Results showed from 640 to 2485 cells per mL of blood (1563 on average), thus similar to the amount of WBC remaining using the fixed cell protocol, and an approximate enrichment factor of 4 logs.

We next tested the feasibility of eliminating the remaining leucocytes after live cells enrichment with the ISET^®^ System using immuno-magnetic beads coated with CD45 antibodies (Fig 1C). We spiked 10 A549 fluorescent cells, counting them one by one by micropipetting, in 1 mL of blood and processed the blood as described in the Methods section 6. In 9 replicate experiments, we found 6 A549 once, 5 A549 once, 4 A549 three times and 3 A549 four times with an average recovery rate of 40%, as shown in Table 6. As compared to the much higher recovery rate obtained after enrichment of live cells (80 to 100%, Table 5), the additional CD45-depletion step causes a loss of 50 to 60% of the enriched cells. However, no remaining leucocytes were identified with this protocol and the morphology of the spiked cells remained intact.

**Table 6:** *In vitro* assay of ISET^®^ sensitivity for enrichment of live tumor cells followed by CD45-immunomagnetic mediated leukocytes depletion.

Blood processed	1 mL
**Spiked tumor cells/test**	10
**Live tumor cells detected by ISET**^**®**^ **after CD45-immuno-magnetic depletion per test (N = 9 tests)**	4
	3
	3
	6
	4
	5
	4
	3
	3
**Total detected/spiked cells**	36/90
**Recovery success rate**	40%

### D- Next Generation sequencing analysis of live tumor cells isolated from blood with the ISET^®^ System

#### D1 Comparative NGS analysis of A549 and HCT116 cells before and after live tumor cell enrichment and 72h *in vitro* culture

In order to further validate the potential clinical interest of our live tumor cells filtration workflow for theranostic applications, we used Next Generation Sequencing (NGS) to study DNA mutations in A549 and HCT116 cells before and after live tumor cells isolation followed by their short term (72h) *in vitro* culture. We used the hotspot cancer panel v2 on the Ion Torrent™ platform as described in the Methods section 7. This amplicon panel can detect 6893 possible COSMIC mutations over 50 oncogenes and tumor suppressor genes [45].

**Fig 10:**
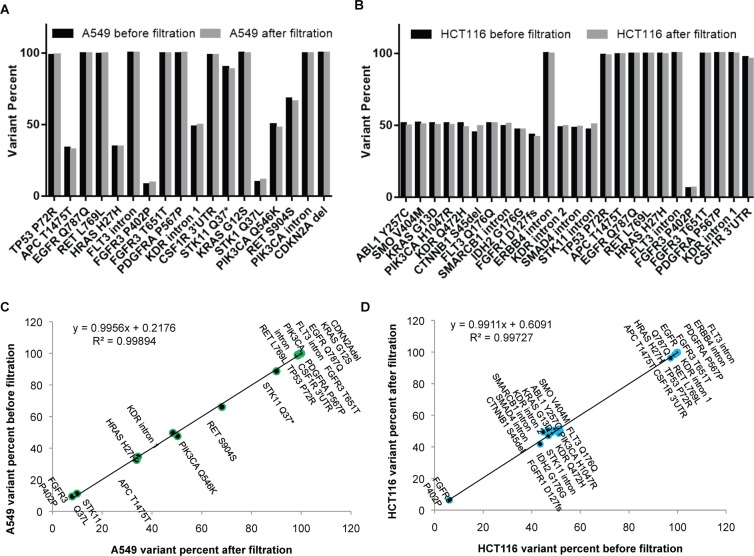
Ion Torrent^TM^ molecular characterization of A549 and HCT116 cells before and after live cell enrichment from blood using ISET^®^. Comparison of variants' mutant allele frequencies in bulk extracted DNA of approximately 10^4^ A549 (A) and 10^4^ HCT116 (B) cells at 72h of *in vitro* culture, before and after live cell enrichment. Correlation of variant allele frequencies in bulk extracted DNA of approximately 10^4^ A549 (C) and 10^4^ HCT116 (D) cells at 72h of *in vitro* culture, before and after live cell enrichment.

Median coverage ranged from 1423X to 2073X among the different samples (S3 Fig). Median sequencing depth across all variants detected was 2957X and 2357X for A549 DNA extracted before and after live enrichment, respectively and 3103X and 3306X for HCT116 DNA extracted before and after live enrichment, respectively (Table D in S1 File**)**. 17 SNP variants were found in A549 DNA including 11 COSMIC mutations classified as most likely pathogenic (Fig 10 and Table D in S1 File). Allele frequencies of all variants were concordant in the sample ‘before filtration’ as compared to the sample ‘after filtration’ (Fig 10A and 10C).

Similarly, 25 variants were found in HCT116 cells including 23 SNPs and 2 INDELs. 16 of these mutations are classified as most likely pathogenic (Table D in S1 File). Allele frequencies of all variants were concordant in the sample ‘before filtration’ as compared to the sample ‘after filtration’ (Fig 10B and 10D). Of note, 4 of these heterozygous mutations were found in the EGFR pathway (PIK3CA H1047R, SMO V404M, ABL1 Y257C and KRAS G13D) in agreement with previous literature [46]. Of note, 11 variants were common in A549 and HCT116.

Interestingly, variants were also identified with confidence at a similar low frequency (6 to 10%) in both samples ‘before filtration’ and ‘after filtration’. (STK1 Q37L and FGFR3 P402P in A549, and FGFR3 P402P in HCT116, Fig 10). This reflects a known phenomenon of genetic and phenotypic heterogeneity of cells lines [47–49] that could be identified in our cell populations.

In A549 DNAs, 3 regions had low coverage (under the 500X threshold) (S3 Fig). Two of these regions were in the CDKN2A gene (amplicon coverage 0) which is depleted in A549 [50]. The last low coverage region was the same in all samples (A549 and HCT116); it corresponds to a variant in CSF1R 3'UTR identified in all the samples, albeit with a coverage under the 500X threshold (Table D in S1 File).

These results provide a proof of principle that the tumor cells’ genetic profile can remain stable after their isolation from blood using our protocol and their short-term culture.

#### D2 Single-cell genetic analysis after live cell enrichment

Since studying the whole genome of individual CTC when they are very rare is important to explore the CTC heterogeneity at the beginning of tumor invasion, we then assessed if our protocol allows single circulating tumor cells' capture and their NGS analysis.

NGS exome libraries were first prepared using the Illumina technology with purified whole-genome amplified DNA from fresh single cells and fixed (not microdissected) single cells isolated by the ISET^®^ System, and from unamplified human gDNA as control. Respectively, we obtained 52, 41, and 44 million reads as well as a genome coverage of 76%, 70%, and 95% with average sequencing depth varying from 20 to 27X. To further assess the quality of our libraries, we next prepared whole genome and exome libraries with whole-genome amplified DNA from 4 fresh single cells and from 4 fixed (not microdissected) cells. The sequencing and mapping results show that we recovered about 70% of the exomes from the analyzed single cells.

Since performing NGS analysis of druggable mutations in very rare single CTC may be clinically relevant, we studied single tumor cells enriched from blood by the ISET^®^ System using the hotspot cancer panel v2 on the Ion Torrent platform as described in the methods section 7. Overall, we analyzed three live single leucocytes, three live single HCT116 cells, six live single A549 cells, and 3 fixed single A549 cells, isolated by the ISET^®^ System, WGA-amplified bulk DNA from HCT116 and A549 cells, as well as bulk genomic DNA from HCT116, A549 cells and the donor’s leukocytes.

**Fig 11:**
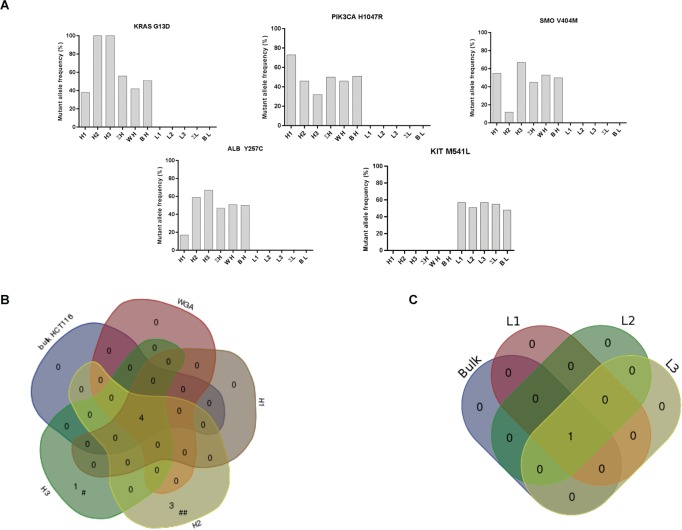
Ion Torrent^TM^ molecular characterization of single HCT116 and leukocytes enriched from blood using ISET^®^. (A) Non-sense variants mutant allele frequency from Catalogue Of Somatic Mutation In Cancer (COSMIC) database in whole genome amplified DNA from single HCT116 tumor cells (H1 to H3), whole genome amplified DNA single leukocytes (L1 to L3), whole genome amplified bulk HCT116 DNA (WH), unamplified bulk HCT116 DNA (BH) and unamplified bulk extracted DNA from the blood donor (BL). (B) Venn diagram showing the concordance of COSMIC non-sense variants determination in whole genome amplified DNA from single HCT116 tumor cells (H1 to H3) as compared to unamplified bulk HCT116 DNA (bulk HCT116) and whole genome amplified bulk HCT116 DNA (WGA). # indicates SMARCB1 R201* and ## indicate CTNNB1 S45P, NOTCH1 L1574P and RB1 E137*. (C) Venn diagram showing the concordance of COSMIC non-sense variants determination in whole genome amplified DNA from single leukocytes (L1 to L3) as compared to unamplified bulk extracted DNA from the blood donor (bulk).

We first evaluated standard sequencing quality control parameters of the DNA obtained from the WGA of our single cells (S1 File and S4 Fig). Then, we studied the presence of hotspot mutations registered in the COSMIC database in the WGA DNA from single cells isolated by the ISET^®^ System. We identified the presence of heterozygous mutations in these 4 hotspots in all HCT116 single cells, WGA-amplified HCT116 DNA and non-amplified HCT116 DNA but not in single leukocytes (Fig 11A). Additionally, when pooling data from three single HCT116 cells, the frequency of mutant allele was consistent with an heterozygous mutation, respectively 56% for KRAS G13D, 50% for PIK3CA H1047R, 45% for SMO V404M, and 51% for ABL1 Y257C (Fig 11A). Furthermore, the coverage for these amplicons ranged from 11 to 2000 (Table E in S1 File). Of note, in HCT116, additional mutations in the APC, FGFR3 and STK11 genes have been described in the literature [46] but are not included in the mutations that can be detected by the hotspot cancer panel v2. A549 cells are known to harbor KRAS G12S homozygous mutation [51] and this mutation was found in all analyzed live A549 single cells (n = 6) as well as fixed A549 single cell (n = 3) (S5 Fig). Furthermore, for all A549 samples, the two amplicons for the *CDKN2A* (p16) gene failed to be detected (Fig 10A and Table D in S1 File), which is consistent with the loss of this locus in this cell line [50]. Interestingly, when analyzing the presence of nonsense COSMIC mutation in leucocytes, we found the presence of heterozygous KIT M541L mutation in all single leucocytes and the matching bulk DNA (Fig 11A and Table E in S1 File). However, although this mutation has been suggested to be associated with leukemia, it is actually a frequent polymorphism among the Caucasian population [52] consistent with our finding.

Finally, we plotted all non-sense COSMIC mutations found in the HCT116 samples and leucocytes and studied personal mutations (i.e. mutations which can only be identified in a single sample). Personal mutations could reflect either heterogeneity (which is not expected among leukocytes) or technical artifacts (error introduced by polymerase during WGA or sequencing) (Fig 11B and 11C). While 0, 1 and 3 personal mutations were identified in the three live single HCT116, personal mutations were not found in single leucocytes (n = 3), nor in WGA-amplified bulk DNA from HCT116, nor in bulk DNA from HCT116 and the donor’s leukocytes. The personal mutations found in HCT116 could represent variants, which are present in less than 5% of the cells (the sensitivity of mutation detection in bulk DNA) or technical artifacts.

Overall, our data suggest that NGS-mediated whole genome analyses, exome analysis and multi-genes panel analyses of tumor cells isolated by the ISET^®^ System are feasible. Of note, we found consistent results with live and fixed tumor cells isolated by the ISET^®^ System, but fixed cells required 3 additional cycles of pre-amplification at the NGS library preparation step (S4A Fig). These data are susceptible to provide reliable and informative results for a deeper understanding of CTC heterogeneity including at the critical early steps of tumor invasion.

## Discussion

In this work, we describe new protocols for the study of circulating tumor cells with a particular focus on their isolation when they are extremely rare. We have assessed these protocols using tumor cells from cell lines up to the immune-molecular characterization of the isolated cells including through their culture. We show that the ISET^®^ system consistently isolates 2 cancer cells added to 1, 5 or 10 mL of blood with an average cell-recovery rate ranging from 83 to 100%. This high recovery is reproducible with different operators and with different devices. The ISET^®^ system’s sensitivity threshold (or LLOD, Lower Limit Of Detection) is one tumor cell per 10 mL of blood, which is the physical limit of the method. We also show that this level of sensitivity is maintained when plasma is collected before isolation of tumor cells from blood as well as when tumor cells are isolated from whole blood in a live form using a suitable modified buffer (without fixation). Finally, we show the feasibility of using the isolated live or fixed cells for single-cell NGS analysis.

Our results show that ISET^®^ allows reproducible, accurate and linear detection and enumeration of cancer cells in blood (Tables 1–5, Figs 4 and 5 and S1 Fig). Our data are consistent with sensitivity and linearity data previously reported by independent teams (S1B and S1C Fig)[12, 27–29]. However, these teams did not extend ISET^®’^s analytical performance and its sensitivity assessment to the extreme limits as we did. Following an independent clinical study which demonstrated the capacity of the ISET^®^ system to detect CTC in patients developing lung cancer years before the detection of tumor nodule by CT-scan [3], we decided to challenge its LLOD by testing it down to a few cells in 10 mL of blood, using an improved and extremely precise protocol. Taken together, our results validate the *in vitro* analytical performance of the ISET^®^ system.

We have assessed the sensitivity specifications of ISET^®^ with tumor cells having different sizes. The median diameters of cultured tumor cells determined on filters typically range from 12 to 20 μm (Fig 3 and Table A in S1 File), consistently with previously published studies [10, 53, 54]. We have successfully isolated intact fixed mouse tumor cells as small as 8.5 μm in diameter (median size on filter 12 μm) using the standard membrane with a nominal pore size of 8 μm (Table 3, Fig 3 and Table A in S1 File). Furthermore, we could enrich by the ISET^®^ System live cells having a median diameter of 10 μm without bias of selection as shown by size assessment before and after filtration (Fig 6).

Other parameters might affect the performance of blood filtration such as cell fragility and biomechanics of the cytoskeleton. It is unclear whether the spectrum of fragility of primary cells is similar to the one of tumor cells from cell lines. However, by fixating the cells structure, the ISET^®^ buffer makes the cells rigid and eliminates the problem of fragility as demonstrated by the very high recovery of individual spiked cells with our approach. Concerning the fragility of primary versus secondary (from cell lines) live tumor cells, our laboratory experience shows that secondary cells are more malignant, but also more fragile than primary tumor cells during *in vitro* manipulation. This is because secondary tumor cells from solid cancers have to be trypsinized to be manipulated and this step damages the cell membrane making it more fragile. Thus, our sensitivity tests performed with secondary live tumor cells are expected to be reliable.

Since sensitivity is a critical issue in the field of CTC detection, we developed an assay able to provide the maximum reliability for sensitivity assessment using a number of tumor cells under 10, i.e. spiking into blood of individually collected and counted fluorescent tumor cells (Tables 1–6, Fig 2A and 2B). For higher numbers of tumor cells (30 to 300), we counted them by dilution, performing repeated counting to adjust the tumor cells' number (Fig 2C). Then, to assess the number of tumor cells successfully isolated by the ISET^®^ System, we counted very carefully the fluorescent cells to avoid any mistake at the detection level (see Methods and Fig 2).

More than 40 distinct methods developed to isolate CTC have been published, and variable levels of *in vitro* sensitivity have been reported (reviewed in Table 7). However, a substantial difficulty in this domain is related not only to the various isolation methods and systems but also to the different protocols used to assess sensitivity, including how tumor cells are counted before spiking into blood and after their isolation from blood. Therefore, in general, comparing the sensitivity of different methods on the only basis of literature data is not completely informative.

**Table 7:** *In vitro* sensitivity and recovery of various CTC methods.

Method type	Method	Principle of the method	Company or Academic lab	*In vitro* LLOD (overall recovery, concentrations tested)	Method for sensitivity and/or recovery assessment	Blood Sample size	Ref
Physical—density	Ficoll^®^	Density separation followed by manual ICC or IF, FACS or RT-PCR	Biochrom, Germany	**10 CTC per 10 mL of blood (42%, 2)**	Spiking of 10 to 1000 cells counted by dilution in 10 mL of blood	variable, typically 10 mL	[38]
Physical—density	OncoQuick^®^	Density separation followed by manual ICC or IF, FACS or RT-PCR	Greiner BioOne, Germany	**10 CTC per 30 mL of blood (42%, 2)**	Spiking of 10 to 1000 cells counted by dilution in 10 mL of blood	up to 30 mL	[38]
Physical- size	ISET^®^ System	Cell size (filter) followed by manual cytopathology, ICC or ICC, FISH, molecular analysis, culture	Rarecells, France	**1 CTC per 10 mL of blood (99.9%, 6)**	Micromanipulation of 2 fluorescent cells added in 1 to 10 mL, dilution 30 to 300 in 1 mL of blood	10 mL	This study
Physical- size	CTC Membrane Microfilter	Cell size (filter) followed by IF	Cote's lab, USA	**5 CTC in 7.5 mL of blood (89%, 1)**	Micromanipulation (5 cells) in 7.5 mL of blood and detection by immunofluorescence or cell dilution (41 cells) and H stain, spiked in 1 mL of blood	7.5 mL	[54, 55]
Physical- size	Canopus	Cell size and deformability	Canopus Bioscience, Canada	**data not found**	data not found		[56]
Physical- size	3D microfilter	Cell size (filter) followed by IF	Cote's lab, USA	**NA (87%, 1)**	Spiking of 340 cells counted by dilution in 1 mL of blood	1 mL	[57]
Physical- size	Screencell^®^ Cyto,MB,CC	Cell size (filter) followed by cytopathology, IF or ICC, FISH, molecular analysis, culture	Screencell, France	**2 CTC in 1 mL of blood (74%, 2)**	Micromanipulation of 2 non fluorescent cells spiked in 1 mL blood, and detection by HE stain	3 mL (6 mL for MB and CC)	[58, 59]
Physical- size	Captor^TM^ / ClearCell^®^ CX	Cell size (microfluidics) followed by cytopathology, IF	Abnova, Singapore	**data not found**	data not found	1 mL	[60]
Physical- size	ClearCell^®^ FX System	Cell size (microfluidics) followed by IF	Clearbridge Biomedic, Singapore	**NA (84%, 1)**	Spiking of 500 fluorescent cells counted by dilution in 7.5 mL of blood	7.5 mL	[61]
Physical- size	Filtration device	Cell size (track-etched filter) followed by IF, molecular analysis	Terstappen's lab, Netherlands	**2 CTC in 1 mL of blood (67%, 6)**	Micromanipulation (2, 10 cells) and dilutions (up to 100000 cells) of fluorescent cells spiked in 1 mL of blood	1–10 mL	[53, 62]
Physical- size	Filtration device	Cell size (microsieve filter) followed by IF, molecular analysis	Terstappen's lab, Netherlands	**2 CTC in 1 mL of blood (58%, 6)**	Micromanipulation (2, 10 cells) and dilutions (up to 30000 cells) of fluorescent cells spiked in 1 mL of blood	1–10 mL	[53, 62]
Physical- size	CellSieve^TM^	Cell size (filter) followed by cytopathology, IF, FISH, molecular analysis	Creativ Microtech, USA	**NA (89% for unfixed cells and 98% for fixed cells, 1)**	Precouting of 50 fluorescent cultured tumor cell on microscope slide and transfer in 7.5 mL of blood	7.5 mL	[63]
Physical- size	MetaCell^®^	Cell size (filter) followed by culture	MetaCell, Czech Republic	**data not found**	data not found	8 mL	[64]
Physical- size	Parsortix^TM^	Cell size and deformability (microfluidics) followed by IF, FISH	Angle, UK	**10 CTC in 2 mL of blood (59%, 3)**	Spiking of fluorescent cells counted by dilution (10 to 100 in 2 mL of blood or 25 to 100 cells in 7.5 mL of blood)	4 mL	[65, 66]
Physical- size	VyCap	Cell size (Microsieve filter) followed by IF, molecular analysis	VyCap	**NA, (67%, 3)**	Spiking of 100 to 200 fluorescent cells (counted by dilution) in 1 mL of media	1 mL	[67]
Physical- size	CelSee	Cell size and deformability (microfluidics) followed by IF, FISH	CelSee Diagnostics	**10 CTC per mL of blood, (84%, 5)**	Spiking of 50 to 2000 cells (counted by dilution) in 2 mL of blood	2 mL	[68]
Physical density or size	Ikoniscope^TM^	Densitiy or Cell size (filter) followed by IF, digital miscroscopy system	Ikonysis, USA	**1 CTC per mL of blood (>90%, 4)**	Micromanipulation (1 to 3 cells) and dilutions (5–1000 cells) in 8 mL of blood	8 mL	[69]
Physical- density and charge	ApoStream^TM^	Density and Cell charge followed by IF	Apocell, USA	**2 CTC in PBMC from 7.5 mL of blood (72%, 4)**	Spiking of 4 to 2600 cells counted by dilution into 1 mL buffer and addition to PBMCs (2 cell lines)	7.5 mL	[70]
Physical- density and size	SmartBiopsy^TM^	Density and cell size followed by IF	Cytogen, Korea	**1 CTC per mL of blood (50%, 3)**	Spiking 10 to 100 fluorescent cell counted by dilution in 3 mL of blood	3 mL	[71]
Marker-based enrichment/ detection	Epithelial Enrich Dynabeads^®^	Magnetics beads coated with anti-EpCAM antibody with optional density gradient separation, followed by ICC or IF	Life technologies, USA	**10 CTC in PBMC from 15 mL of blood (74%, 3)**	Spiking in PBMC of 10 to 1000 cells counted by dilution	variable up to 15 mL	[72]
Marker-based enrichment/ detection	CellSearch^TM^	Immunomagnetic capture with anti-EpCAM antibody followed by IF (EpCAM, CK, CD45)	Veridex (J&J), USA	**1 CTC per 7.5 mL of blood (85%, 5)**	Spiking of 4 to 1142 cells counted by dilution in 7.5 mL of blood, regression analysis and extrapolation based on Poisson distribution of rare events	7.5 mL	[73]
Marker-based enrichment/ detection	Maintrac^TM^	Density and Milteny anti-EpCAM beads followed by IF (CK, CD45)	Pachmann lab., Germany	**NA (83%, 1)**	Spiking of cells counted by dilution (down to 60 cells) in 1 to 20 mL of blood	1 mL	[74]
Marker-based enrichment/ detection	AdnaTest	Immunocapture (Anti-Epcam Dynabeads) followed by RT-PCR or IF	AdnaGen, Germany	**2 CTC per 5 mL of blood (100%, 1)**	Individual spiking of 2 to 10 cells and 100 cells counted by dilution in 5 mL of blood	5 mL	[75]
Marker-based enrichment/ detection	CTC-Chip	Anti-EpCAM Capture in microfluidic format followed by Cytopathology, IF, FISH, molecular analysis	On-Q-ity, USA	**1 CTC in 1 billion cells (>60%, 6)**	Spiking of 50 to 50,000 cells counted by dilution in 1 mL of blood	1 to 5 mL	[76]
Marker-based enrichment/ detection	Magsweeper	Immunocapture using anti-EpCAM antibody followed by IF, molecular analysis	Illumina, USA	**NA (62%, 1)**	Spiking of 50 fluorescent cells counted by dilution in 3 mL of blood	9 mL	[77]
Marker-based enrichment/ detection	MACS / Carcinoma Cell Enrichment and Detection kit	Ficoll and MACS (EpCAM, HER2, MCSP, NG2, CD45, CKs)	Mitlenyi Biotech, Germany	**2 CTC per 8 mL of blood (61%, 4)**	Spiking of 1 to 10000 cells counted by cell sorter in 8 mL of blood	8 mL	[78]
Marker-based enrichment/ detection	HB Chip	Anti-EpCAM Capture in microfluidic format followed by IF, cytopathology	Toner/harber lab, USA	**NA (92%, 1)**	Spiking of fluorescent 500 fluorescent cells counted by dilution in 1 mL of blood	4 mL	[79]
Marker-based enrichment/ detection	CEE^TM^ platform	Pre-processing with leucosep tubes, Flow-dependant Immuno-capture (antibody cocktail) followed by IF, FISH	Biocept, USA	**NA (70%, 1)**	Spiking of 150 cells counted by cell dilution in 1 mL	10 mL	[80]
Marker-based enrichment/ detection	Nanovelcro	Anti-Epcam Capture in microfluidic format followed by IF	Tseng's lab, USA	**NA (95%, 5)**	Spiking of 50 to 1000 cells counted by dilution in 1 mL of blood	1 mL	[81]
Marker-based enrichment/ detection	Microtube device	Ficoll+Immunocapture (Anti-EpCAM) + E-selectin	Biocystics, USA	**NA (50%, 5)**	Spiking of 20 to 700 fluorescent cells counted by dilution in 4 mL diluted blood (1:1)	7.5 mL	[41]
Marker-based enrichment/ detection	CTCScope^TM^	Density followed by IF, RNA FISH	ACD, USA	**1 CTC per 5 mL of blood (72%, 17 one replicate)**	Counting under microscope (1–92 cells) and transfer of the drop in to 5 mL of blood	7.5 mL	[82]
Marker-based enrichment/ detection	HDCTC	No enrichment, IF panCK and image analysis	EpicScience, USA	**10 CTC in 2 mL of blood (99%, 4)**	Spiking of 10 to 300 cells counted by dilution in 2 mL of blood and regression analysis	4 mL	[83]
Marker-based enrichment/ detection	CellCollector^®^	In vivo immunocapture (Anti-EpCAM), IF	Gilupi, Germany	**not evaluated, in vivo assay**	not evaluated, in vivo assay	30 min, *in vivo*	[84]
Marker-based enrichment/ detection	Isoflux	Density gradient (ficoll) and Immunocapture using magnetics beads conjugated with anti-EpCAM followed by IF, molecular analysis	Fluxion Biosciences, USA	**4 CTC in 7 mL of blood (75%, 8)**	Spiking of 4 to 200 fluorescent cells counted by dilution in 7 mL of blood	7 mL	[85]
Marker-based enrichment/ detection	Biofluidica CTC Detection System	Immunocapture using anti-EpCAM antibody followed by IF	Biofluidica, USA	**NA (90%, 1)**	Spiking of 500 fluorescent cells counted by dilution in 7.5 mL of blood	7.5 mL	[86]
Marker-based enrichment/ detection	Cytotrack^TM^	No enrichment, IF, image analysis	Cytotrack, Denmark	**5 CTC in 7.5 mL of blood (68%, 3)**	Spiking of 10 to 100 cells counted by cell sorter in 7.5 mL of blood	7.5 mL	[87]
Marker-based enrichment/ detection	posCTC iChip	Cell size (microfluidics) with positive selection with anti-EpCAM, IF, RT-PCR, cytopathology, culture	Toner/harber lab, USA	**10 cells in 10 mL (91%, 2)**	Spiking of 10, 200 or 1000 fluorescent cells (various cell lines) in 1 mL of blood	variable, up to 10 mL	[88, 89]
Marker-based enrichment/ detection	Liquid Biopsy^®^	Immunocapture using surface markers (EpCaM, CK…) followed by IF, molecular analysis	Cynvenio Biosystems, USA	**3 CTC per mL of blood (74%, 6)**	Spiking of 3 to 900 cells by dilution in 1 mL of blood	8 mL	[90]
Leucocyte Depletion	anti-CD45 Dynabeads^®^	Magnetics beads coated with anti-CD45 antibody with optional density gradient separation, followed by ICC-IF	Life technologies, USA	**10 CTC in PBMC from 15 mL of blood (65%, 3)**	Spiking of 10 to 1000 cells counted by dilution in PBMC	variable, up to 15 mL	[72]
Leucocyte Depletion	RosetteSep^TM^	Negative selection (gradient and antibody cocktail)	Stem Cell Technologies, Canada	**NA (43%, 2)**	Spiking of 25 to 250 cells counted by dilution in 4 mL of blood	typically 20 mL	[91]
Leucocyte depletion and physical (size)	negCTC iChip	Cell size (microfluidics) with negative selection with anti-CD45, followed by IF, RT-PCR, cytopathology, culture	Toner/harber lab, USA	**NA, (97%, 1)**	Spiking of 1000 fluorescent cells (various cell lines) in 1 mL of blood	variable, up to 10 mL	[88, 89]
Leucocyte depletion and physical (size)	Canpatrol^TM^	Anti-CD45 dynabead, filtration, followed by IF, FISH, DNA mutation analysis	SurExam Bio-Tech, China	**6 CTC in 5 mL of blood (88%, 4)**	Spiking of 10 to 200 fluorescent cells counted by dilution in 5 mL of blood, immunofluorescence and FISH	5 mL	[92]
Functional	EPISPOT	RosetteSep negative selection followed by secretion assay	Alix-Panabieres, France	**1 CTC per 10 mL of blood (67%, 4)**	Spiking of 100000 to 1 cells counted by dilution in 10 mL of blood	10 mL	[93, 94]
Functional	Telomescan^®^	No enrichment, telemerase expression (GFP fluorescence)	Oncolys Biopharma, Japan	**10 CTC in 5 mL of blood (60%, 3)**	Spiking of 10 to 1000 Cells by dilution in 5 mL of blood	5 mL	[95]
Functional	CAM	Density (optional), adhesion property and IF	Vitatex, USA	**1 CTC per mL of blood (80%, 3)**	Spiking of 3 to 3000 cells counted by cell sorter in 3 mL of blood	2 to 20 mL	[96]

List of CTC methods and their definitions of sensitivity and/or recovery as shown in peer-reviewed publications. CTC methods are presented according to the principle of the method and per chronological order. Categories include methods based on physical properties (i.e. cell density, cell size or cell charge), enrichment and/or detection with markers (EpCAM, Cytokeratins, etc.), depletion of leucocytes and functional assays (which require the cells to be alive). Recovery is calculated when cells are spiked into the relevant matrix, at a minimum of five concentration levels covering the linear range of the assay (according to the FDA definition). Here, overall recovery is the average recovery for the range of concentration tested and number of concentration tested as indicated in the table (each concentration has n>2 replicates unless specified).

Concerning the different cell-size based methods developed to isolate CTC, they are characterized by different filtration parameters (type of filters, buffer or media used to dilute blood, type of device and filtration pressure …) which determine their different performance (summarized in Table F in S1 File). Therefore, despite the fact that the word “filtration” intuitively sounds as an easy and generic approach to separate large and small elements, blood filtration to isolate tumor cells is not at all depending only on the pore size. It is not like filtrating sand (size: 0,2–2 mm) to extract shells (size: few cm). The size difference between white blood cells (8–15 μm) and tumor cells (15–30 μm) is very small, creating a competition of blood cells for the filter’s pores and making tumor cells' isolation without cell loss or damage an ambitious technical challenge.

Our *in vitro* data showing the very high sensitivity of ISET^®^ are consistent with *in vivo* data previously published by independent teams, showing the higher *in vivo* sensitivity of ISET^®^ as compared to CellSearch^TM^ [12, 17, 20, 22, 23, 30–32, 97] (Reviewed in [2]).

*In vivo* sensitivity for circulating cancer cells detection is affected by several factors: 1) how long and how the blood is stored before CTC isolation, 2) the number of steps involved in the isolation process (every step leads to cell loss) and its rapidity, 3) how cells are captured and 4) how cells are enumerated. For instance, the use of markers for CTC capture/enumeration has an impact on how many CTC are detected/counted, depending on the markers' level of expression in cancer cells.

Stabilization of tumor cells in blood for a few days without cell loss and without damage to cell morphology remains an unmet goal. If available, proper stabilization would allow centralization of CTC analysis without loss of sensitivity. CellSave (Veridex, USA) and Streck (Streck, USA) tubes have been used to stabilize tumor cells in blood but analytical reports have shown that they lead to the loss of 40 to 60% of tumor cells spiked in blood [98]. In fact, blood cell membranes remain intact for up to 5 hours [99] after blood collection and degrade rapidly and dramatically after that (Dr Carla Ferreri, personal communication). For this reason, the ISET^®^ system manufacturer’s guidelines indicate that blood has to be treated by the ISET^®^ System within 5 hours after blood collection.

The exceptional sensitivity of the ISET^®^ System relies on the following aspects: 1) blood is treated soon after collection, 2) blood treatment to prepare blood for ISET^®^ filtration is rapid and efficient 3) the number of steps before the “isolation” step is minimum: blood is just collected from the patient, diluted and filtered, 4) fixed cells are collected with no additional steps by the filter and only one additional step is required to collect live cells by pipetting, 5) the filter characteristics avoid losing cells larger than mature lymphocytes, 6) the blood aspiration system can be modulated and allows to stick large cells to the filter without loss, or concentrate them in a small volume over the filter, 6) the system does not relies on the use of markers for isolation nor for collection not for identification of tumor cells. Combined together, these characteristics play a key role in the ISET^®^ sensitivity for isolation of circulating tumor cells.

Analytical sensitivity cannot be tested by using primary tumor cells as tumor cells from a patient’s blood cannot be counted upstream of any further analysis to isolate them. Therefore, we performed precision studies with cells from cell lines made fluorescent, mixed with blood and carefully counted before and after their isolation. However, the clinical sensitivity of ISET^®^ (percentage of patients with CTC in a specific clinical setting) has been studied by independent authors in comparative and non-comparative studies and shown to be extremely high (Fig 3 in Ref [2] and Table I in S1 File).

We also note that, in the field of CTC, a high sensitivity is clinically relevant only if associated with high specificity. Since circulating rare non-tumor cells may be present in the blood of patients with cancer, high sensitivity with low specificity can lead to mistakes in clinical decisions.

**Fig 12:**
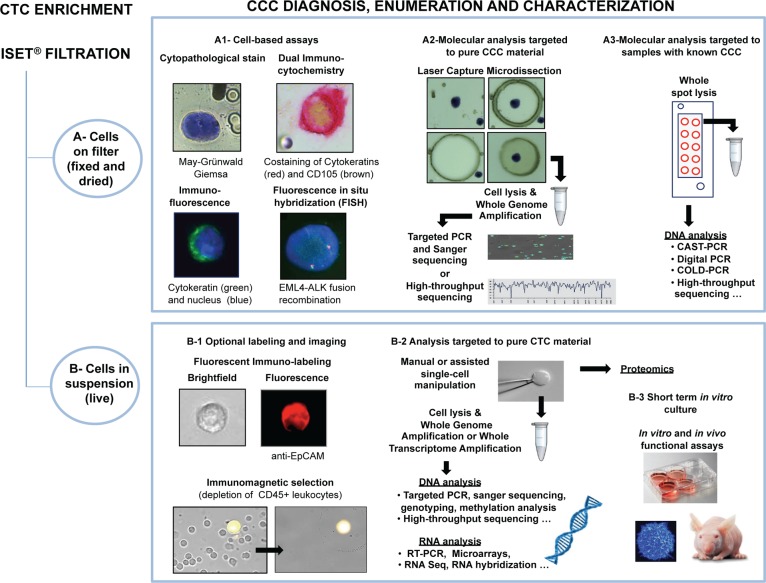
CTC characterization possibilities after CTC isolation or enrichment by ISET^®^. (A) CTC characterization possibilities after fixed CTC isolation by ISET®. (A1)-Enriched cells are stained on the filter and CCC can be identified by cytopathology [16] and precisely counted. CTC can also be characterized by simple or multiple immuno-fluorescence-labeling [11, 17, 18], simple or multiple immuno-cytochemistry labeling [10, 12, 19, 20], or FISH [10, 21–23]. (A2) CTC can be characterized by molecular analysis (PCR, next generation sequencing …) after laser microdissection of the filter ([10, 17, 24–26, 100]). (A3) CTC can be characterized by molecular DNA and RNA analyses without microdissection using sensitive methods for detection of mutation such as Competitive Allele-Specific TaqMan® (CAST)-PCR, co-amplification at lower denaturation temperature (COLD)-PCR, Digital PCR, next generation sequencing, or RT-PCR [27]. (B) CTC characterization possibilities after live CTC enrichment by ISET®. (B1) Enriched CTC are collected in suspension and can be optionally immuno-stained or further enriched by CD45 depletion. CTC can be precisely counted after immune-labeling. (B2) Molecular analysis such as PCR and sanger sequencing, next generation sequencing (this study), RNA analysis, DNA methylation analysis [101] and proteomic [102] can be targeted to CTC after single cell isolation by micromanipulation (manual or by robot such as CellCelector^TM^) or dielectrophoresis (DEPArray^TM^). Additionally, mutation detection can be performed without single cell isolation on samples in which CTC have been identified using sensitive mutation-detection methods such as CAST-PCR, COLD-PCR, Digital PCR or next generation sequencing. (B3) Samples can be used for short-term culture, *in vivo* or *in vitro* expansion and functional assays.

Regarding specificity, ISET^®^ filtration does not perform any detection of CTC *per se*. It is meant to isolate CTC without bias and with a minimum CTC loss. Specificity is brought by the subsequent step of cytopathology. Since tumor cells are isolated intact without damage, CTC characterization is possible by a variety of downstream approaches (Fig 12). As reported and recognized, cytopathology is the only clinically validated method to diagnose tumor cells [2].

Obtaining intact tumor cell morphology is a key factor for a reliable and diagnostic identification and characterization of CTC. Our team, as well as other authors, have previously emphasized the need of specific markers able to reliably identify the presence of tumor cells in blood. Such markers are not known at present [2, 4, 13–15], and this leaves cytopathology as the only diagnostic approach with regards of the presence of tumor cells in blood. In this setting, it is noteworthy that several authors have found similar morphological and immunomorphological characteristics in CTC isolated by ISET^®^ and in tumor cells from the correspondent tumor tissues [10, 12, 32, 103, 104]. Blood cytopathology is diagnostic “per se” and can also be used to study circulating tumor cells after their isolation from blood and culture [105].

The hypothetical loss of small CTC by the indiscriminated filtration approach, mentioned in various literature sources [7, 8, 12, 106, 107] has never been assessed in comparative studies using different filtration approaches, nor clearly related to intact cells or to naked nuclei or cell fragments. In fact, even tumor cells from Small Cell Lung Cancer (SCLC) have been detected by ISET^®^ [11, 104, 108, 109]. These cells have a diameter ranging from 11.2 to 17.7 μm, i.e. 1.4 to 2.2 times larger than the 8 μm lymphocytes [110].

According to cytopathological criteria [16, 30, 111], cancer cells from patients are usually larger than 16 microns. The presence in blood of intact cancer cells smaller than 8 microns, i.e. smaller than mature lymphocytes, has never been diagnosed in patients [13], which comes in support of using ISET^®^ to isolate all types of CTC from blood.

Hofman *et al*. have provided data supporting the key role of cytopathology to identify CTC. They have published a blind multicenter study involving 10 cytopathologists and 808 cases [16] demonstrating that the classical criteria used in standard cytopathology are valid to reliably identify CTC when cells are isolated from blood using the ISET^®^ System. Hofman *et al*. showed that, under these conditions, blood cytopathology has the same advantages and the same limitations as classical cytopathological analyses. In fact, cytopathology is not recommended to diagnose cells from parathyroid and thyroid adenoma [16]. Overall, to this date, 475 healthy donor and 211 patients with benign disease have been tested by ISET^®^ setting the *in vivo* specificity of ISET^®^ blood cytopathology at 98.3% (Summarized in Table G in S1 File).

The need for specificity in detection of CTC has prompted us to introduce the term “Circulating Cancer Cells” (CCC) [2] referring to tumor cells isolated from blood without loss and without antibody related bias and diagnostically identified by cytopathology. Such cancer cells are expected to be of clinical interest [2, 4] as shown by independent clinical studies using ISET^®^ [3, 17, 18, 22, 24, 26, 35, 103, 111–115] (Reviewed in Table H in S1 File).

Cancer cells isolated by ISET^®^ have been studied across various cancer types and stages, as well as in patients at risk of developing cancer [10–12, 16–27, 29–32, 35, 97, 103, 104, 108, 111–119] (Reviewed in Table I in S1 File). The global number of CCC and CCM has been reported to be higher in patients with advanced disease. For instance, for patients with NSCLC, Krebs *et al*. reported the following median number of CCC per 10 mL: 5.3 in Stage IIIA patients (n = 5), 11.3 in Stage IIIB patients (n = 12) and 50.7 in Stage IV patients (n = 23) [12] (Table I in S1 File).

The detection of CCC by ISET^®^ has been proven by independent teams and studies to have a prognostic value in follow-up prospective studies, in particular in patients with localized lung cancer, liver cancer and uveal melanoma [24, 35, 112] (Table H in S1 File).

Although larger trials are needed, the above mentioned results are consistent with animal studies which have shown that the risk of developing metastases correlates directly with the number of cancer cells in blood and that a higher risk is associated with the presence of CTM [120–123].

Hofman’s team also brought a conclusive demonstration that the ISET^®^ blood cytopathology is diagnostic for reliable identification of cancer cells in blood. They showed, in an independent and prospective study, the ability of this assay to detect “sentinel tumor cells” in patients at risk of developing lung cancer years before the detection of the tumor nodule by CT-scan, thus demonstrating a new way for invasive cancers' early diagnosis and treatment [3].

Early diffusion of tumor cells in blood was first observed in animal models [1, 124, 125]. On average, about one out of 1000 cancer cells from the tumor mass is thought to be able to invade the blood compartment [126]. This data is consistent with earlier work using animal models of fibrosarcoma and breast cancer [127, 128]. If we take this value as a reference, a tumor containing 500 000 cells with a size of about 0.5 mm in diameter (~0.5 mg of tumor [129]) would spread 500 cancer cells into the 5 liters of human blood. Such a small tumor could therefore be detected by the ISET^®^ system which LLOD is 1 tumor cell per 10 mL of blood. Even if these calculations are an estimate and individual cancers have different invasion capabilities, they provide a reference value consistent with the possible detection of tumor cells before an invasive tumor reaches the size of a few mm in diameter which makes it detectable by imaging. At this stage, it could spread around 4000 tumor cells in blood, equivalent to 8 tumor cells per 10 mL of blood.

However, we do not expect that tumor cells' spreading is continuous and constant throughout the day. As it frequently happens with biological phenomenon, spreading could be variable according to other biological factors such as, for instance, waves of angiogenesis and blood flow. Consistent with this view are preliminary *in vivo* data related to myeloma cells [130] and fetal cells showing circadian rhythms of circulation as well as increased cells' circulation after physical exercise [131]. Although such aspects have not been thoroughly explored yet, they raise the important issue of the best-standardized way to collect blood for the maximum CTC detection and enumeration.

We have reported a new protocol allowing collection of plasma without any loss of intact tumor cells (Fig 1B, Table 4 and S1D Fig). By using this protocol we avoid wasting blood obtained from ill patients. The collection of plasma is useful to detect cell-free DNA, cell-free RNA and exosomes as complementary investigations in cancer patients. While only CTC detection can provide information about the invasive cancer potential, the study of plasma markers may add interesting complementary information [132]. A recent study has compared the presence of tumor cells detected by ISET^®^ and methylated RASSF1A cfDNA in 68 matching blood samples from controls and patients with melanoma, allowing to discriminate melanoma patients from controls. However, 7 out of the 68 healthy controls (10%) were scored positive for the presence of methylated cfDNA in plasma, highlighting the challenge to find specific genetic markers for cfDNA analyses [119].

Cancer cells isolated from blood without antibody-related bias can be further characterized, in a second line of investigation, using immune-molecular approaches (Figs 7, 9 and 12). In this perspective, ISET^®^ allows performing multiplexing tests. In fact, Krebs *et al*. [12] have reported that enumeration of tumor cells on 4 ISET^®^ spots (corresponding to the filtration of 4 mL of blood) is as reliable as enumerating them on 10 spots (corresponding to the filtration of 10 mL of blood). Our data further validate this finding with intra-assay precision and accuracy below the 15% cutoff when enumerating tumor cells on four spots (Fig 5), showing that it is possible to count tumor cells by ISET^®^ on 4 spots leaving the other 6 spots for CTC immune-molecular characterization. This approach has been demonstrated to be valid for numbers of CTC ranging from 6 to 14 per mL of blood, by our results (Fig 5 and S2 Fig) and for number of CTC ranging from 11 to 26 per mL of blood by Krebs *et al*. [12].

The clinical value of ISET^®^ is predictably related to its capacity to isolate all types of tumor cells including tumor cells in Epithelial to Mesenchymal Transition (EMT), expressing mesenchymal markers such as Vimentin, but not (or barely) expressing epithelial markers (EpCAM or cytokeratin). These cells are lost by methods relying on epithelial markers for CTC isolation and/or detection [12, 20]. The presence of mesenchymal cancer cells in blood has been shown to be relevant in terms of prognostic value in patients with pancreatic cancer [18, 32], and lung cancer [12, 22, 32, 35]. Detection of tumor cells in EMT is also relevant for the development of reliable companion diagnostics tests. For instance, using ISET^®^, Pailler *et al*. have reported the presence of a recurrent ALK-rearrangement in CTC which also expressed mesenchymal markers, consistently with a clonal tumor cells' selection [22]. Independent studies have reported the interest of ISET^®^ for *in situ* detection of several theranostic biomarkers including ALK recombination, ROS1 recombination, KRAS mutations, BRAF V600E mutation and HER2 amplification [19, 21–23, 25, 26].

To facilitate further fundamental studies, we have determined that ISET^®^ can isolate cancer cells from mice blood (Fig 3, Table 3 and S6 Fig). The study of animal and human CTC with the same tool (independently from potential antibody related bias and issues of species' cross reactivity) should foster studies in animals in particular for early detection of invasive cancers. ISET^®^ has also been used to isolate tumor cells from rats [133], monkeys and pigs (unpublished data).

Fundamental studies need live circulating tumor cells. They are required in particular for culture-based assays allowing drug sensitivity tests and further molecular investigations.

We developed a new protocol to enrich live tumor cells from blood without immune-related bias (Fig 1C) using a dedicated Rarecells^®^ live buffer and using the Rarecells^®^ device and cartridge in a different way. We show that it allows a recovery rate of 80 to 100% of tumor cells and a sensitivity threshold (LLOD) of 1 tumor cells per mL of blood in spiking tests using individually captured single cells (Fig 2B and Table 5). Our results demonstrate that this new protocol for isolation from blood of live tumor cells does not induce a cell-size related bias of selection, including for the smallest cells we tested (MMTV-PyMT, Fig 6).

Biomechanics of the cytoskeleton might affect the performance of the method when using unfixed samples. Articles in the literature indicate that non-malignant tumor cell lines are stiffer in comparison to malignant tumor cell lines (and also less prone to migration and invasion) [43, 134]. Studies with cells obtained from patients’ ascites (ovarian cancer) or derived from oral squamous carcinoma have shown that cancer cells from a given tumor cells' population exhibit a varying degree of stiffness, which is similar to the stiffness of cancer cell lines [135, 136]. In addition, stiffness is also correlated to the EMT status of cells: the stiffest/least invasive cell lines expressed more E-Cadherin and less Vimentin, while the compliant/most invasive cell lines expressed less E-Cadherin and more Vimentin [136]. In our tests with live cells, we used both cell lines with epithelial phenotype (LNCaP) and mesenchymal phenotype (A549, H2228, MMTV-PyMT) (details of markers expressed by LNCaP, H2228 and A549 according to the database of DSMZ (Deutsche Sammlung von Mikroorganismen und Zellkulturen) and by MMTV-PyMT according to [137]). Furthermore, our results obtained using quantitative fluorescence analyses show that the level of EpCAM expression of MCF-7 tumor cells is similar before and after their live cell enrichment by ISET^®^ (Fig 7).

In this report, we have also shown that live A549 tumor cells isolated from blood using ISET^®^ can be cultured and expanded *in vitro* for at least 5 days (Fig 8), demonstrating that the buffer and protocol used to isolate live tumor cells from blood allows their growth. Interestingly, we noticed that no leucocytes remained alive after 3 days of culture.

In addition, we have assessed cytoskeleton markers by confocal analysis of F-actin and acetylated α-tubulin (Fig 9) after live tumor cells isolation and culture for 72h. These markers are known to be therapeutic targets [138, 139]. The microscopy assay we used has been shown to directly correlate with Atomic Force Microscopy, a cell stiffness assay [140]. We have compared the profiles of two cell lines, A549 and H2228. Distribution of F-actin and acetyl-α-tubulin were found to be conserved (or restored) after 72h of *in vitro* culture following live tumor cells isolation using the ISET^®^ system.

Furthermore, in the aim of studying theranostic mutations to guide the therapeutic choices, we have demonstrated that, in our model, the basic profile of mutations, assessed with the Hotspot Cancer Panel V2 (Thermofisher, USA) does not change after isolation by ISET^®^ of tumor cells from blood and their growth in culture (Fig 10, S3 Fig and Table D in S1 File) for 72 hours. This new data may stimulate similar important analyses performed on CTC from cancer patients.

Taken together, these data suggest that the new ISET^®^ protocol to isolate live tumor cells does not modify the cell phenotype and genotype. Consistently, tumor cells viability after their isolation from blood remained very high (>85%) (Fig 6 and Table C in S1 File).

Since the first report of successful CTC-derived xenografts (CDX) in 2000 [141], several research publications have focused on the *ex vivo* propagation of CTC via short-term *in vitro* culture [64, 142, 143], direct injection into immuno-compromised mice [109, 144, 145] or long-term 3D culture by establishment of prostate and colorectal cancer cell lines derived from CTC [142, 146]. However, long-term *in vitro* culture of CTC remains a technical challenge in the field, with a generally very low percentage of successful growth. Further knowledge about culture conditions is required to obtain *in vitro* growth of heterogeneous and very rare CTC populations. However the first requirement in this aim is the possibility to extract tumor cells from blood without selection bias, keeping their phenotype, genotype viability and growth capabilities potentially unaffected.

The new protocol we have developed for isolation of live tumor cells from blood is highly sensitive, rapid, direct and does not alter their biological characteristics, thus it should help further studies focused on CTC’ *ex vivo* expansion and analysis.

Until now *ex vivo* culture of CTC has been achieved using samples from patients having several hundreds of CTC per 7.5 mL of blood. The success rates of CTC expansion in culture remains low [143, 146, 147] across various isolation methods and culture conditions. CTC from patients with SCLC and NSCLC were successfully implanted into nude mice, creating CTC-derived Xerograph (CDX) for subsequent *in vivo* drug testing [17, 109]. Some data indicate that CTC which are competent for metastasis and able to proliferate in culture are undifferentiated and EpCAM-negative [17, 147]. The excellent sensitivity of the antibody-independent ISET^®^ live CTC isolation protocol (Table 5) and the proof that it allows tumor cell growth in culture (Figs 8 and 9) should help culture assays for fundamental CTC studies and *ex vivo* drug testing (Fig 12).

Very low numbers of live tumor cells can be isolated from blood by ISET^®^ practically without loss and with a limited contamination of leukocytes. The number of residual leukocytes, when ISET^®^ is performed with classical filters with pores of 8-micron nominal size, is variable from few hundreds to more than 1000. We did not report results in term of “purity” (defined as the number of target cells divided by the total number of remaining cells) since such calculation is misleading given the variable number of CTC detected *in vivo* (Table I in S1 File) and of tumor cells spiked in blood for *in vitro* sensitivity tests.

Of note, the elimination of leukocytes can be increased by using filters having different parameters, including the pore size, and suitable for maximum elimination of leukocytes while retaining large tumor cells and tumor microemboli.

In order to reduce the number of contaminating leukocytes, we have further developed a protocol using CD45-coated magnetic beads, which was proven to achieve a complete elimination of leukocytes. As a drawback, adding the CD45 selection leads to loss of 50 to 60% of the spiked tumor cells (Table 6). However, given the very high sensitivity of CTC enrichment obtained by ISET^®^ before CD45-depletion, the new protocol remains an interesting option to collect live CTC not contaminated by leukocytes and without antibody-related bias for further RNA, DNA and protein studies and for culture assays (Fig 12).

Single cancer cells capture by their microdissection from the filter has been proven to allow targeted mutation analyses [10, 24, 25]. The new ISET^®^ protocols to enrich live or fixed CTC from blood without sticking them to the filter provide new possibilities for extensive single tumor cells RNA and DNA molecular studies while avoiding microdissection (Figs 10 and 12). Individual CTC can be simply captured manually after enrichment from blood by micropipetting or by using current commercial approaches (such as CellCelector^TM^ or DEPArray^TM^ or others).

Cancer cells' genetic heterogeneity is increasingly investigated at single cell resolution in tissues [148, 149]. Some studies have reported copy number variation by NGS analysis on single CTC identified by CellSearch^TM^ [109, 150–153], MagSweeper [154] or Epic Sciences [155] but, with these approaches, CTC heterogeneity is predictably underestimated by the EpCAM-related selection bias. An optimized workflow for molecular characterization of individual CTC, which are sensitively isolated from blood without antibody related bias, is expected to be an attractive tool.

We reported here an optimized workflow including: 1) highly sensitive isolation of live CTC from blood without bias, 2) capture of individual CTC, 3) single cell whole genome amplification, 4) efficient high throughput sequencing using a multi-gene panel analysis with the Ion Torrent^TM^ approach.

Our results show the feasibility of applying the AmpliSeq hotspot cancer panel V2 on Ion Torrent^TM^ to both live cells and fixed cells enriched from blood by ISET^®^ and recovered in suspension, assessing mutations on a variety of oncogenes including KRAS (Fig 11 and S5 Fig). The study of 3 individual tumor cells allowed the detection of mutations with the same allele frequency detected in the parent cell line (Fig 11B and 11C). To our knowledge these are the first results obtained by NGS analysis on CTC sensitively isolated from blood without bias related to the use of antibodies. The protocol should help discovering theranostic mutations in CTC spread at the early steps of tumor invasion.

Overall, our results demonstrate that ISET^®^ is an open platform which allows a highly sensitive and unbiased isolation from blood of fixed tumor cells for reliable identification and immune-molecular study, and of live tumor cells for culture and immune-molecular analyses. We have used cells from cell lines as a model of technical approach with potential to be applied to clinical blood samples. These technical improvements should foster studies targeting circulating cancer cells' detection and characterization, in particular at the early steps of tumor invasion.

## Supporting Information

S1 Fig Additional analytical performance data. (A) Recovery experiments with dilutions of HeLa and MCF-7 cells. Mean in % of recovered HeLa and MCF-7 cells observed on ISET^®^ filter. 50 or 100 HeLa cells were added into 1 mL whole blood after counting by dilution. 50 MCF-7 were added to 5 mL of whole blood after counting by dilution. (B) Linearity experiment reported by Chinen *et al*. 2014. 25, 50, 100 and 150 HT1080 cells (counted by dilution) were added to 1 blood before processing by ISET^®^ (in triplicates). (C) In vitro sensitivity experiments reported by Krebs *et al*. 2012 and De Giorgi *et al*. 2010. 1 SK-MEL-28 cell isolated by micropipetting was added to 1 mL of blood before processing by ISET^®^ (n = 9 tests). 1, 10 and 50 NCI-H1299 cells isolated by micropipetting were added to 1 mL of blood before processing by ISET^®^ (in triplicates). (D) Fixed tumor cell isolated in sensitivity test after collection of plasma. Recovered cell (A and B) stained with Cell Tracker^TM^ Orange and observed with the TRITC microscopic filter (A: 20X objective, Scale bar: 4 μm, B: Scale bar: 8 μm) (C) observed with bright field filter, (D) observed with bright field filter after a MGG staining.
(TIFF)

S2 Fig Assessment of ISET^®^ intra-assay accuracy and precision with dilutions of MCF-7. About 50 MCF-7 were spiked into 5 mL of blood (n = 5 experiments, 29 to 70 cells per 5 mL). Cell counting were performed without careful recounting. The number of tumor cells found on each spot after ISET® filtration (each corresponding to the filtration of 1 mL of blood) was recorded. Experiments were done on 5 spots but for intra-assay precision and accuracy only assessment of the comparison of combinations of 1, 2, 3 and 4 spots are relevant. The only combination with the 5 spots was the reference. Four spots exhibited a representative mean tumor cells value. (A) Bar chart with the mean tumor cell number per spot and corresponding standard error of the mean (error bars) depending on the number of spots analyzed. Error bars (which correspond to the Standard Error, i.e. standard deviation divided by the squared root of the number of combinations) are calculated using the standard deviation of different combinations of 4 spots, 3 spots, 2 spots or 1 spot. If only one spot is considered, standard deviation is higher than when counting 4 spots. Thus error bars indicate the increased precision and accuracy when tumor cells are counted on 4 spots as compared to 3, 2 and one spot. (B) Table indicating the number of tumor cells found on each spot for each of the five experiments, the 95% confidence interval (CI), the precision and the accuracy depending on the number of spots analyzed (1 to 4) as compared to the analysis on five spots.
(TIFF)

S3 Fig Ion Torrent^TM^ sequencing quality control parameters of A549 and HCT116 populations before and after live cell ISET^®^ enrichment. (A) Total number of reads, reads on target, low coverage regions, coverage and coverage heterogeneity for each of the 4 bulk DNA samples. (B) Sequencing depth determination (percentage of target region coverage) for each of the 4 bulk DNA samples.
(TIFF)

S4 Fig Ion Torrent^TM^ sequencing quality control parameters of bulk extracted DNA and WGA DNA from single cell enriched from blood using ISET^®^. (A) Amplicon mapping and sequencing depth uniformity across whole genome amplified single cells and bulk amplified and unamplified DNA controls. (B) Overview coverage plots of sequencing reads obtained from: (B1) whole genome amplified DNA from a single live A549 cell, (B2) whole genome amplified DNA from a single fixed A549 cell, (B3) whole genome amplified from bulk A549 extracted DNA, (B4) control unamplified bulk A549 DNA and (B5) control unamplified bulk DNA extracted from healthy donor blood. (C) Average amplicon coverage on pooled data from whole genome amplified single cells and bulk whole genome amplified and unamplified DNA controls.
(TIFF)

S5 Fig Mutant KRAS G12S allele frequency by Ion Torrent^TM^ in single cells enriched from blood using ISET^®^. Mutant KRAS G12S allele frequency was determined by high throughput sequencing of whole genome amplified single A549 cells, both live and fixed, as well as bulk whole genome amplified (WGA) and unamplified (bulk) DNA controls extracted from A549 tumor cells.
(TIFF)

S6 Fig Isolation of cancer cells from mouse blood. Clusters of cells with malignant features observed in the blood of two MMTV-PyMT mice. Scale bar 8 microns. Mouse blood (200 μL, kind gift of Dr. S. Humbert-Institut Curie, France) was collected, using a 2 mL syringe prefilled with 8 mg of sterile K_3_EDTA, from two 14-week old MMTV-PyMT mice under anesthesia by retro-orbital puncture, according to the local ethics rules, transferred to a microcentrifuge tube and kept under gentle agitation before its treatment by ISET^®^ within 3 hours after collection. ISET^®^ was performed by diluting mouse blood 1 to 10 with the buffer and filtering it using the standard protocol in one small compartment of the Rarecells^®^ Block. Up to 10 different mice samples can be processed with the same cartridge.
(TIFF)

S1 File Supplemental text and all supplemental tables. Table A in S1 File: Cell size (in microns) on ISET^®^ filters of various cell lines. Cells from human and mouse tumor cell lines were incubated 3 min with the buffer without blood and collected on standard (8 micron-pore) filters. MMTV-PyMT*: values measured for MMTV-PyMT cells isolated using 5 micron-pore filters. Table B in S1 File: Percentage of cell Recovery of the ISET^®^ platform and Precision and Accuracy of spiking tests. A549, MCF-7, MMTV-PyMT and HeLa cells were counted by micromanipulation (for tests with 1 to 3 cells) or dilution (for tests with 30 to 300 cells) and spiked into 1 to 10 mL of blood as indicated. Precision and Accuracy among all these independent tests were calculated as described in the methods. Precision is assessed via calculation of percent coefficient of variation (%CV) that is equal to 0% when data are perfectly precise. Accuracy estimated via %Error that is equal to 0% when data are perfectly accurate. Table C in S1 File: Cell size and viability measurement before and after ISET^®^ filtration of live cells. Cells from human and mouse tumor cell lines were incubated 3 min with the Live Buffer without blood and collected on standard (8 micron-pore) filters. Table D in S1 File: Variant list and allele frequency measured by Ion Torrent in cell populations. Cat = Sophia DDM category of pathogenicity, Chrs = chromosome, var_% = percentage of variant. Table E in S1 File: Non-sense COSMIC mutant allele coverage, amplicon coverage and allele frequency measured by Ion Torrent^TM^ in WGA-amplified single cells enriched from blood by ISET^®^. Table F in S1 File: Main parameters and sensitivity of CTC filtration methods. *1 Overall recovery = average recovery for the range of concentration tested, number of concentration tested (n>2 replicates for each concentration unless specified). *2 elimination of red blood cells by centrifugation. Notes: methods that use Ficoll or equivalent prior to filtration or without any peer reviewed publication are not included in this table. form. = formaldehyde. NA = not available. Table G in S1 File: CCC detection by ISET^®^ in the blood of healthy donors and patients with benign diseases. Table H in S1 File: Studies reporting prognostic value of CCC/CCM detected by ISET® and ISET® longitudinal follow-up studies. CNHC: circulating non-hematological cells include cells with benign, uncertain and malignant features. Overall Survival (OS) is defined by the NIH-NCO as the percentage of patient in a study or treatment group who are still alive for a certain period of time after they were diagnosed with or started treatment for a disease, such as cancer. Progression-free survival (PFS) is defined by the NIH-NCO as length of time during and after the treatment of a disease, such as cancer, that a patient lives with the disease but it does not get worse. Disease-free survival (DFS) is defined by the NIH-NCO as the length of time after primary treatment for a cancer that the patient survives without any signs or symptoms of that cancer. Hazard ratio (HR) is defined by the NIH-NCO as, a measure of how often a particular event happens in one group compared to how often it happens in another group, over time. A hazard ratio of one means that there is no difference in survival between the two groups. A hazard ratio of greater than one or less than one means that survival was better in one of the groups. Prognostic biomarker: biomarker that can be used to estimate the chance of recovery from a disease or the chance of the disease recurring. Predictive biomarker: biomarker that can be used to help predict whether a person’s cancer will respond to a specific treatment. Predictive factor may also describe something that increases a person’s risk of developing a condition or disease. Table I in S1 File: CCC/CCM detected by ISET^®^ in the blood of cancer patients. HCC: hepatocellular carcinoma, BC: breast cancer, PrC: prostate cancer, PaC: pancreatic cancer, NSCLC: Non-Small Cell Lung Cancer, SCLC: Small Cell Lung cancer, CC: Colorectal cancer, KC: Kidney cancer, HNC: Head and Neck carcinoma, Esophageal carcinoma: EC, Pleural Mesothelioma: PM, Sarcoma: Sarc, UM: uveal melanoma, CM: cutaneous melanoma, M0: localized, M1: metastatic, na: not available, ‡ if several blood sampling time points are reported, number of patients with CCCs/CCMs and CCC/CTMs number are only indicated for the baseline time point, * if average CCC and CCM numbers are not available, the median or range are provided as indicated in the table. Usually the mean is calculated over the whole population of patients (including patients without CCCs), unless specified otherwise in the table.
(DOCX)
