# Supplementary figures and images for: Technical Insights into Highly Sensitive Isolation and Molecular Characterization of Fixed and Live Circulating Tumor Cells for Early Detection of Tumor Invasion
Source: PLoS One. 2017 Jan 6;12(1):e0169427. doi: 10.1371/journal.pone.0169427 (PMC5218415; doi:10.1371/journal.pone.0169427)

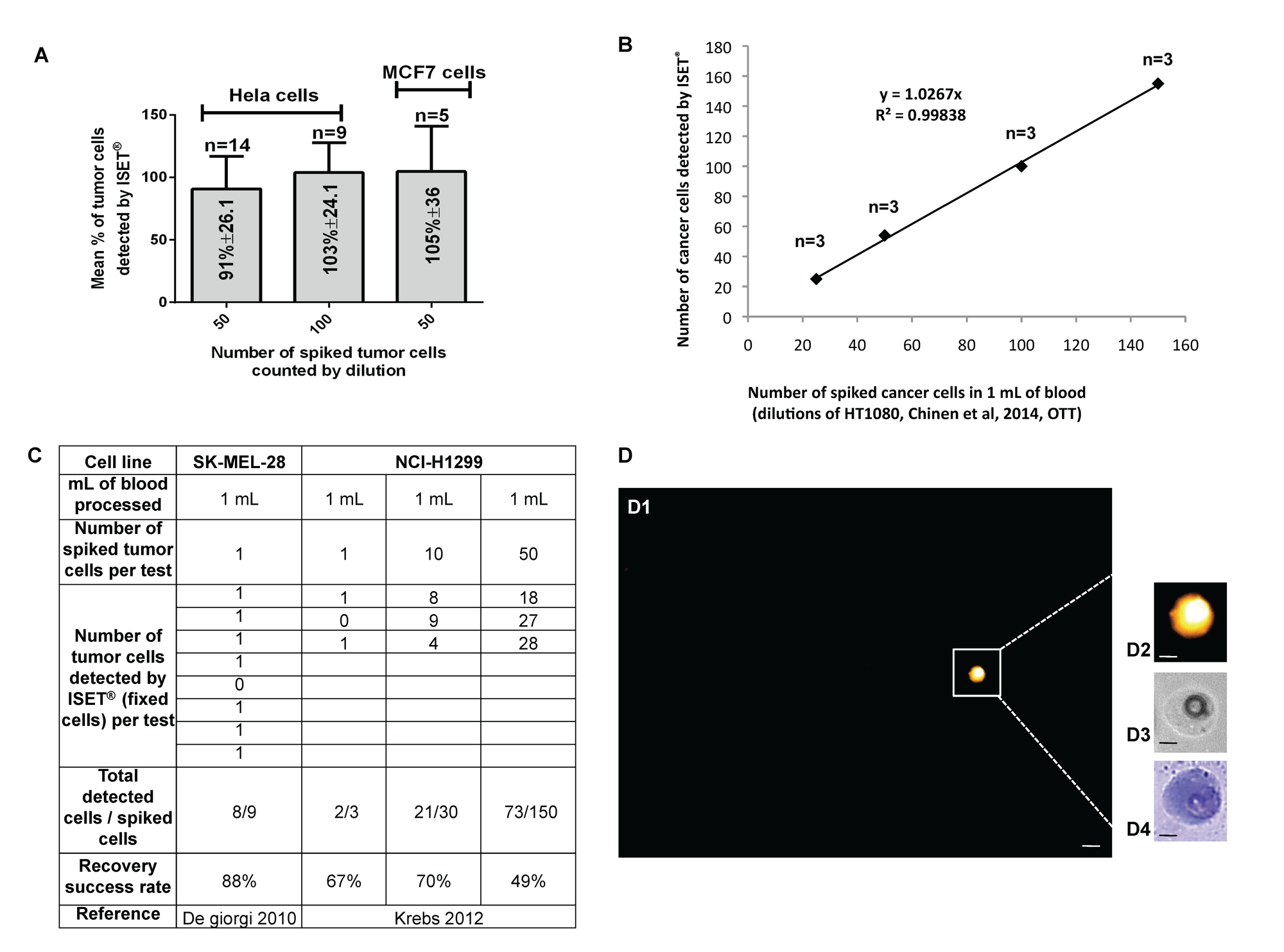

Supplement: S1 Fig — (A) Recovery experiments with dilutions of HeLa and MCF-7 cells. Mean in % of recovered HeLa and MCF-7 cells observed on ISET® filter. 50 or 100 HeLa cells were added into 1 mL whole blood after counting by dilution. 50 MCF-7 were added to 5 mL of whole blood after counting by dilution. (B) Linearity experiment reported by Chinen et al. 2014. 25, 50, 100 and 150 HT1080 cells (counted by dilution) were added to 1 blood before processing by ISET® (in triplicates). (C) In vitro sensitivity experiments reported by Krebs et al. 2012 and De Giorgi et al. 2010. 1 SK-MEL-28 cell isolated by micropipetting was added to 1 mL of blood before processing by ISET® (n = 9 tests). 1, 10 and 50 NCI-H1299 cells isolated by micropipetting were added to 1 mL of blood before processing by ISET® (in triplicates). (D) Fixed tumor cell isolated in sensitivity test after collection of plasma. Recovered cell (A and B) stained with Cell TrackerTM Orange and observed with the TRITC microscopic filter (A: 20X objective, Scale bar: 4 μm, B: Scale bar: 8 μm) (C) observed with bright field filter, (D) observed with bright field filter after a MGG staining. (TIFF) [file pone.0169427.s001.tiff]

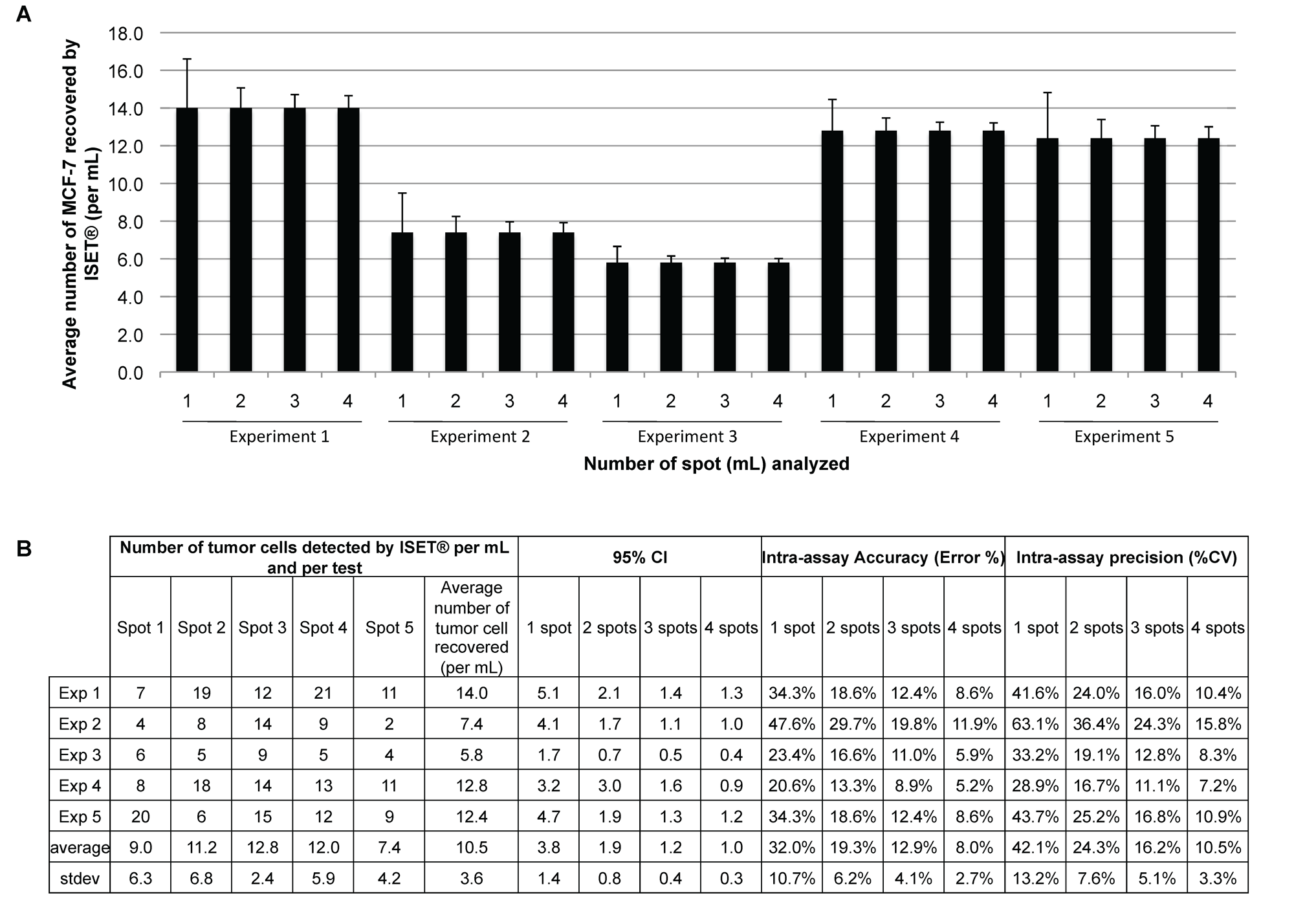

Supplement: S2 Fig — About 50 MCF-7 were spiked into 5 mL of blood (n = 5 experiments, 29 to 70 cells per 5 mL). Cell counting were performed without careful recounting. The number of tumor cells found on each spot after ISET® filtration (each corresponding to the filtration of 1 mL of blood) was recorded. Experiments were done on 5 spots but for intra-assay precision and accuracy only assessment of the comparison of combinations of 1, 2, 3 and 4 spots are relevant. The only combination with the 5 spots was the reference. Four spots exhibited a representative mean tumor cells value. (A) Bar chart with the mean tumor cell number per spot and corresponding standard error of the mean (error bars) depending on the number of spots analyzed. Error bars (which correspond to the Standard Error, i.e. standard deviation divided by the squared root of the number of combinations) are calculated using the standard deviation of different combinations of 4 spots, 3 spots, 2 spots or 1 spot. If only one spot is considered, standard deviation is higher than when counting 4 spots. Thus error bars indicate the increased precision and accuracy when tumor cells are counted on 4 spots as compared to 3, 2 and one spot. (B) Table indicating the number of tumor cells found on each spot for each of the five experiments, the 95% confidence interval (CI), the precision and the accuracy depending on the number of spots analyzed (1 to 4) as compared to the analysis on five spots. (TIFF) [file pone.0169427.s002.tiff]

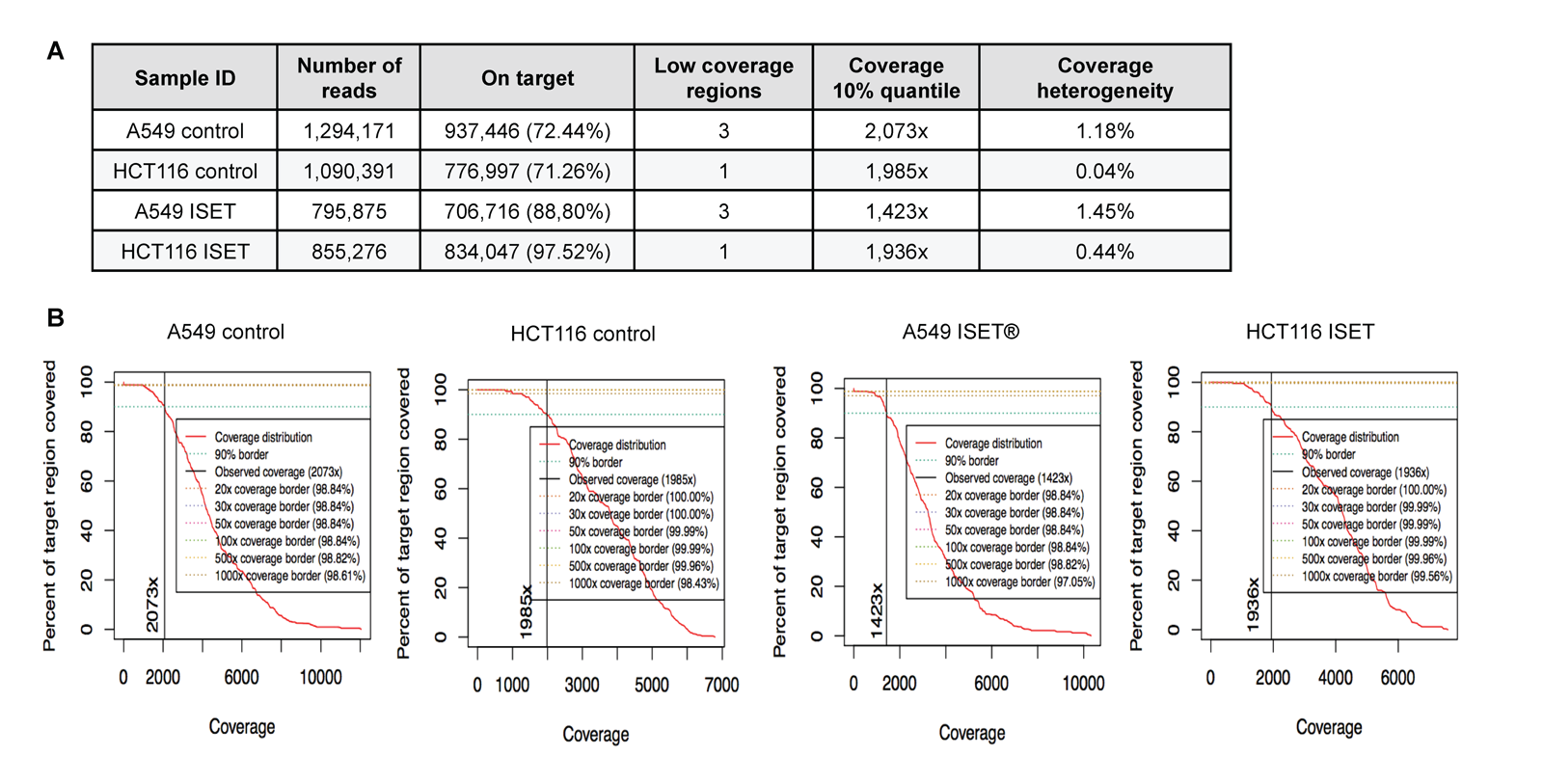

Supplement: S3 Fig — (A) Total number of reads, reads on target, low coverage regions, coverage and coverage heterogeneity for each of the 4 bulk DNA samples. (B) Sequencing depth determination (percentage of target region coverage) for each of the 4 bulk DNA samples. (TIFF) [file pone.0169427.s003.tiff]

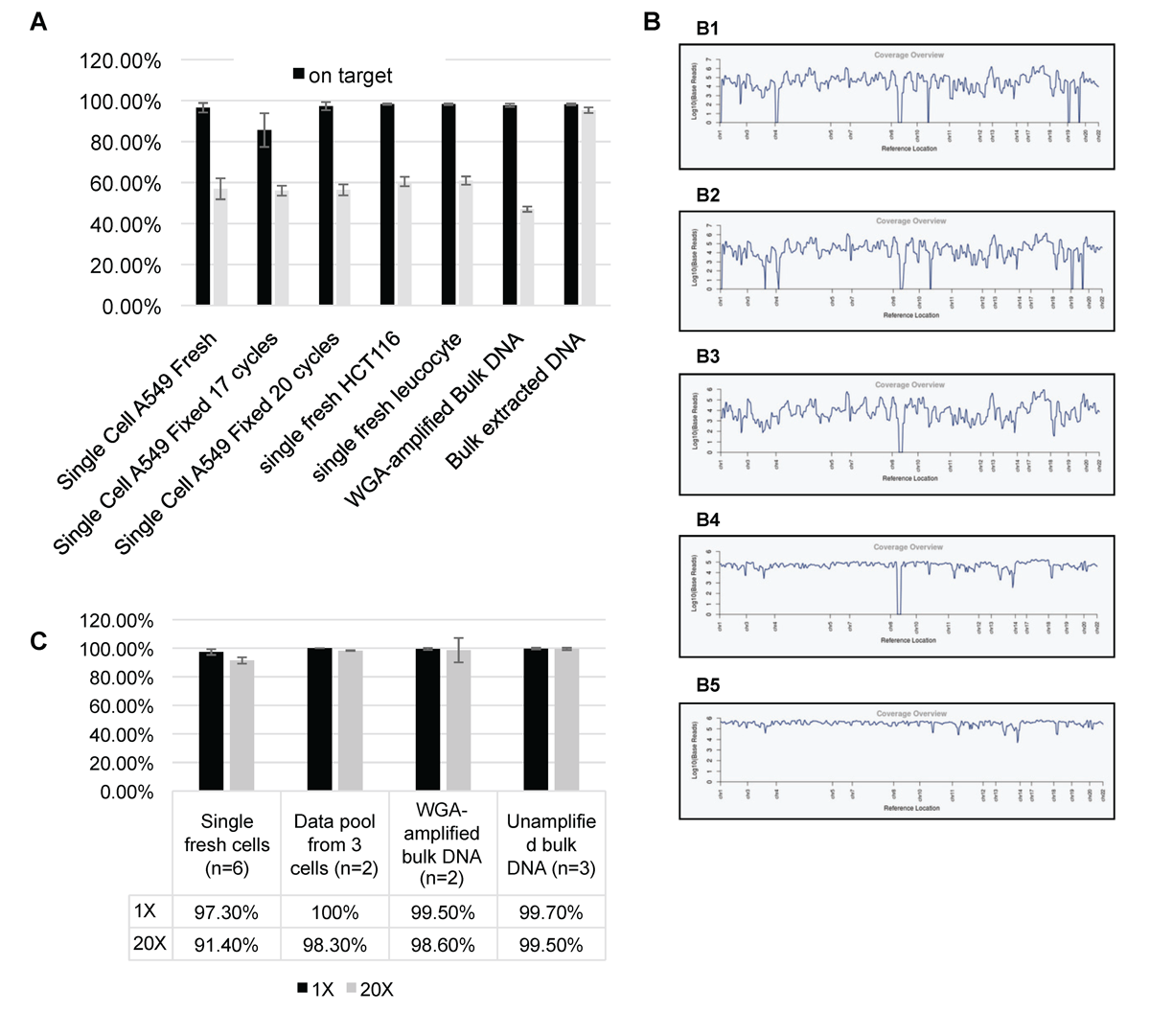

Supplement: S4 Fig — (A) Amplicon mapping and sequencing depth uniformity across whole genome amplified single cells and bulk amplified and unamplified DNA controls. (B) Overview coverage plots of sequencing reads obtained from: (B1) whole genome amplified DNA from a single live A549 cell, (B2) whole genome amplified DNA from a single fixed A549 cell, (B3) whole genome amplified from bulk A549 extracted DNA, (B4) control unamplified bulk A549 DNA and (B5) control unamplified bulk DNA extracted from healthy donor blood. (C) Average amplicon coverage on pooled data from whole genome amplified single cells and bulk whole genome amplified and unamplified DNA controls. (TIFF) [file pone.0169427.s004.tiff]

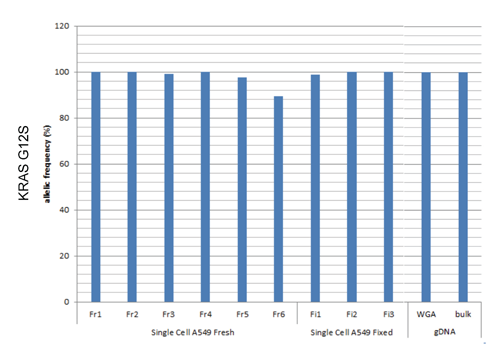

Supplement: S5 Fig — Mutant KRAS G12S allele frequency was determined by high throughput sequencing of whole genome amplified single A549 cells, both live and fixed, as well as bulk whole genome amplified (WGA) and unamplified (bulk) DNA controls extracted from A549 tumor cells. (TIFF) [file pone.0169427.s005.tiff]

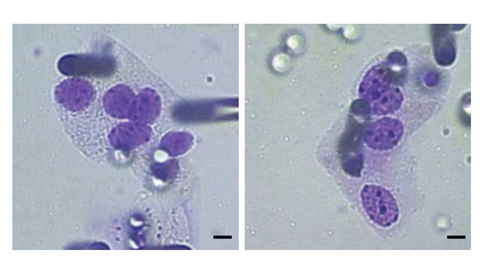

Supplement: S6 Fig — Clusters of cells with malignant features observed in the blood of two MMTV-PyMT mice. Scale bar 8 microns. Mouse blood (200 μL, kind gift of Dr. S. Humbert-Institut Curie, France) was collected, using a 2 mL syringe prefilled with 8 mg of sterile K3EDTA, from two 14-week old MMTV-PyMT mice under anesthesia by retro-orbital puncture, according to the local ethics rules, transferred to a microcentrifuge tube and kept under gentle agitation before its treatment by ISET® within 3 hours after collection. ISET® was performed by diluting mouse blood 1 to 10 with the buffer and filtering it using the standard protocol in one small compartment of the Rarecells® Block. Up to 10 different mice samples can be processed with the same cartridge. (TIFF) [file pone.0169427.s006.tiff]
