# Supplementary material for: Technical Insights into Highly Sensitive Isolation and Molecular Characterization of Fixed and Live Circulating Tumor Cells for Early Detection of Tumor Invasion
Source: PLoS One. 2017 Jan 6;12(1):e0169427. doi: 10.1371/journal.pone.0169427 (PMC5218415; doi:10.1371/journal.pone.0169427)
Supplement: S1 File — Table A in S1 File: Cell size (in microns) on ISET® filters of various cell lines. Cells from human and mouse tumor cell lines were incubated 3 min with the buffer without blood and collected on standard (8 micron-pore) filters. MMTV-PyMT*: values measured for MMTV-PyMT cells isolated using 5 micron-pore filters. Table B in S1 File: Percentage of cell Recovery of the ISET® platform and Precision and Accuracy of spiking tests. A549, MCF-7, MMTV-PyMT and HeLa cells were counted by micromanipulation (for tests with 1 to 3 cells) or dilution (for tests with 30 to 300 cells) and spiked into 1 to 10 mL of blood as indicated. Precision and Accuracy among all these independent tests were calculated as described in the methods. Precision is assessed via calculation of percent coefficient of variation (%CV) that is equal to 0% when data are perfectly precise. Accuracy estimated via %Error that is equal to 0% when data are perfectly accurate. Table C in S1 File: Cell size and viability measurement before and after ISET® filtration of live cells. Cells from human and mouse tumor cell lines were incubated 3 min with the Live Buffer without blood and collected on standard (8 micron-pore) filters. Table D in S1 File: Variant list and allele frequency measured by Ion Torrent in cell populations. Cat = Sophia DDM category of pathogenicity, Chrs = chromosome, var_% = percentage of variant. Table E in S1 File: Non-sense COSMIC mutant allele coverage, amplicon coverage and allele frequency measured by Ion TorrentTM in WGA-amplified single cells enriched from blood by ISET®. Table F in S1 File: Main parameters and sensitivity of CTC filtration methods. *1 Overall recovery = average recovery for the range of concentration tested, number of concentration tested (n>2 replicates for each concentration unless specified). *2 elimination of red blood cells by centrifugation. Notes: methods that use Ficoll or equivalent prior to filtration or without any peer reviewed publication are not includ [file pone.0169427.s007.docx]

**Supplemental Information**

**Supplemental text**

We evaluated standard sequencing quality control parameters of the DNA obtained from the WGA of our single cells (**S4 Fig.**). For all the samples, we obtained sufficient number of mapped reads, on average about 625400 mapped reads (range 238403 to 2149603). Using 17 cycles of pre-amplification for library preparation, most of the reads obtained from the WGA from fresh single cells, WGA-amplified bulk DNA and bulk DNA aligned very well with their targets (>98%). In contrast, the reads obtained from fixed single cells were imperfectly aligned using the standard library protocol preparation with 17 cycles of preamplification (average 86%, n=3). With FFPE protocol using 20 cycles of preamplification, most of the reads from the WGA from fixed single cells were properly aligned (average 97%, n=3) (**S4 Fig.**). Uniformity of sequencing depth was close to 100% in bulk DNA reflecting the quasi-perfect evenness of coverage. In contrast, for all single cell WGA samples as well as WGA-amplified bulk DNA samples, uniformity was reduced to about 50% (**S4A** **Fig.**). This reflects a less uniform sequencing depth after WGA that is expected because of the variability introduced by WGA. Analysis of coverage plots (**S4B** **Fig.**) further confirmed that the sequencing of WGA DNA from single cells was noisier than WGA-amplified bulk genomic DNA but with very few amplicon failure. Amplicon coverage in ISET^®^ enriched single-cell WGA DNA was about 97.3 % at 1X depth. At 20X depth, amplicon coverage was still over 90% but lower with the single cell WGA DNAs than with the bulk DNA and WGA-amplified bulk DNA (91.4 %, 99.5% and 98.6% respectively). Amplicon coverage at 20X depth increased to 98.3% when pooling data from 3 single cells (**S4C Fig.**) suggesting that the analysis of three single cells amplified by WGA could be sufficient to achieve a similar quality as for bulk DNA.

**Supplementary Tables**

**Table A. Cell size (in microns) of cells from various cell lines isolated using ISET^®^ filters**

| **Cell line** | **A549** | **MCF-7** | **HeLa** | **LNCaP** | **MMTV-PyMT** | **MMTV-PyMT*** |
| --- | --- | --- | --- | --- | --- | --- |
| minimum | 18.0 | 15.1 | 12.5 | 14.2 | 9.5 | 8.5 |
| 1st quartile | 20.6 | 17.4 | 14.5 | 17.4 | 11.4 | 10.3 |
| median | 22.0 | 19.0 | 15.8 | 19.2 | 12.0 | 11.1 |
| 3rd quartile | 25.0 | 21.1 | 17.1 | 22.4 | 13.3 | 12.3 |
| maximum | 44.0 | 34.7 | 19.4 | 29.8 | 16.8 | 19.0 |
| Mean | 23.2 | 19.6 | 15.8 | 20 | 12.4 | 11.4 |
| Standard deviation | 4.4 | 3.3 | 1.8 | 3.3 | 1.6 | 1.7 |

Cells from human and mouse tumor cell lines were incubated 3 min with the buffer without blood and collected on standard (8 micron-pore) filters. MMTV-PyMT*: values measured for MMTV-PyMT cells isolated using 5 micron-pore filters.

**Table B. Percentage of cell recovery of the ISET^®^ platform and Precision and Accuracy of spiking tests**

| **Cell line** | **mL of blood analyzed** | **Number of spiked cells** | **Total number of tests** | **Average number of recovered cells** | **Average percentage of recovered cells** | **Precision**  **(%CV)** | **Accuracy (%Error)** | **Reference** |
| --- | --- | --- | --- | --- | --- | --- | --- | --- |
| A549 | 10 mL | 2 | 6 | 2 | 100% | 0% | 0% | Table 2 |
|  | 5 mL | 2 | 6 | 1.7 | 83% | 31% | 17% | Table 2 |
|  | 1 mL | 0 | 4 | 0 | 0% |  |  | Fig 4 |
|  | 1 mL | 2 | 12 | 1.75 | 88% | 26% | 12% | Table 1 |
|  | 1 mL | 2 | 12 | 1.67 | 83% | 39% | 17% | Table 1 |
|  | 1 mL | 2 | 6 | 1.67 | 83% | 31% | 17% | Table 2 |
|  | 1 mL | 2 | 30 | 1.7 | 85% | 31% | 15% | Overall (Fig 4) |
|  | 1 mL | 30 | 9 | 28.2 | 94% | 17% | 6% | Fig 4 |
|  | 1 mL | 100 | 13 | 84.7 | 85% | 21% | 15% | Fig 4 |
|  | 1 mL | 300 | 2 | 333.5 | 111% | 6% | 11% | Fig 4 |
| MCF-7 | 1 mL | 1 | 10 | 0.9 | 90% | 35% | 10% | Table 3 |
|  | 5 mL | 50 | 5 | 52.4 | 105% | 35% | 5% | Fig S1A |
| HeLa | 1 mL | 1 | 3 | 1 | 100% | 0% | 0% | Table 3 |
|  | 1 mL | 3 | 3 | 3 | 100% | 0% | 0% | Table 3 |
|  | 1 mL | 50 | 14 | 45.4 | 91% | 29% | 9% | Fig S1A |
|  | 1 mL | 100 | 9 | 104 | 104% | 23% | 4% | Fig S1A |
| MMTV-PyMT | 1 mL | 2 | 4 | 1.5 | 75% | 38% | 25% | Table 3 |

A549, MCF-7, MMTV-PyMT and HeLa cells were counted by micromanipulation (for tests with 1 to 3 cells) or dilution (for tests with 30 to 300 cells) and spiked into 1 to 10 mL of blood as indicated. Precision and Accuracy among all these independent tests were calculated as described in the methods. Precision is assessed via calculation of percent coefficient of variation (%CV) that is equal to 0% when data are perfectly precise. Accuracy estimated via %Error that is equal to 0% when data are perfectly accurate.

**Table C. Cell size and viability measurement before and after filtration of live cells**

| **Cell line** | **Replicate** | **Viability** | **Average Viability** | **Smallest size (µm)** | **Largest size (µm)** | **Median size (µm)** | **Average of median size (µm)** |
| --- | --- | --- | --- | --- | --- | --- | --- |
| A549 before filtration | 1 | 95% | **98%** | 10 | 17 | 14 | **14** |
|  | 2 | 100% |  | 10 | 17 | 14 |  |
|  | 3 | 99% |  | 9 | 17 | 14 |  |
| A549 after filtration | 1 | 95% | **93%** | 10 | 17 | 14 | **14** |
|  | 2 | 89% |  | 11 | 19 | 14 |  |
|  | 3 | 94% |  | 9 | 17 | 14 |  |
| MMTV-PyMT before filtration | 1 | 99% | **97%** | 7 | 14 | 10 | **9.6** |
|  | 2 | 97% |  | 7 | 14 | 10 |  |
|  | 3 | 95% |  | 7 | 12 | 9 |  |
| MMTV-PyMT after filtration | 1 | 90% | **85%** | 7 | 12 | 9 | **9.6** |
|  | 2 | 86% |  | 7 | 13 | 10 |  |
|  | 3 | 80% |  | 7 | 14 | 10 |  |

Cells from human and mouse tumor cell lines were incubated 3 min with the Live Buffer without blood and collected on standard (8 micron-pore) filters.

**Table D. Variant list and allele frequency measured by Ion Torrent™ in cell populations**

**1. Bulk extracted DNA from A549 control or after ISET^®^ filtration and culture**

|  |  |  |  |  |  |  |  |  | **A549 control** | | **A549 ISET** | |
| --- | --- | --- | --- | --- | --- | --- | --- | --- | --- | --- | --- | --- |
| gene | **Overlap**  **Known** | **type** | **Cat** | **Coding**  **Consequence** | **Chrs.** | **Genome**  **position** | **c.DNA** | **protein** | **Depth** | **var_%** | **Depth** | **var_%** |
| STK11 | COSM12925 | SNP | A | nonsense | 19 | 1207021 | c.109C>T | p.Gln37* | **2956** | **90.05** | **4974** | **88.68** |
| KRAS | COSM1152506 | SNP | A | missense | 12 | 25398285 | c.34G>A | p.Gly12Ser | **4777** | **99.9** | **3932** | **99.87** |
| STK11 | COSM48783 | SNP | A | missense | 19 | 1207022 | c.110A>T | p.Gln37Leu | **2957** | **9.87** | **4974** | **11.24** |
| PIK3CA | COSM766 | SNP | A | missense | 3 | 178936094 | c.1636C>A | p.Gln546Lys | **2803** | **50.27** | **1698** | **47.59** |
| TP53 | COSM3766190 | SNP | A | missense | 17 | 7579472 | c.215C>G | p.Pro72Arg | **7372** | **98.55** | **6875** | **99.07** |
| APC | COSM3760869 | SNP | A | synonymous | 5 | 112175770 | c.4425G>A | p.= (p.Thr1475Thr) | **4663** | **33.86** | **5313** | **32.51** |
| EGFR | COSM1451600 | SNP | A | synonymous | 7 | 55249063 | c.2361G>A | p.= (p.Gln787Gln) | **3213** | **99.88** | **2357** | **99.79** |
| RET | COSM4418406 | SNP | A | synonymous | 10 | 43613843 | c.2307G>T | p.= (p.Leu769Leu) | **6491** | **99.49** | **4304** | **99.77** |
| RET | COSM3751779 | SNP | A | synonymous | 10 | 43615633 | c.2712C>G | p.= (p.Ser904Ser) | **6058** | **68.11** | **5237** | **66.05** |
| HRAS | COSM3752426 | SNP | A | synonymous | 11 | 534242 | c.81T>C | p.= (p.His27His) | **4320** | **34.42** | **2246** | **34.59** |
| FLT3 | COSM3999060 | SNP | A | intronic | 13 | 28610183 | c.1310-3T>C |  | **3872** | **99.92** | **2865** | **100** |
| FGFR3 |  | SNP | C | synonymous | 4 | 1806187 | c.1206C>A | p.= (p.Pro402Pro) | **2369** | **7.94** | **1154** | **9.45** |
| FGFR3 |  | SNP | C | synonymous | 4 | 1807894 | c.1953G>A | p.= (p.Thr651Thr) | **2526** | **99.8** | **2008** | **99.75** |
| PDGFRA |  | SNP | C | synonymous | 4 | 55141055 | c.1701A>G | p.= (p.Pro567Pro) | **2915** | **99.76** | **1953** | **99.9** |
| PIK3CA |  | SNP | C | intronic | 3 | 178917005 | c.352+40A>G |  | **1750** | **99.54** | **918** | **99.89** |
| KDR |  | SNP | C | intronic | 4 | 55980239 | c.798+54G>A |  | **2400** | **48.42** | **2153** | **49.65** |
| CSF1R |  | SNP | C | 3'UTR | 5 | 149433596 | c.*35_*36delCAinsTC |  | **497** | **98.59** | **218** | **98.62** |

**2. Bulk extracted DNA from HCT-116 control or after ISET^®^ filtration and culture**

|  |  |  |  |  |  |  |  |  | **HCT-116 control** | | **HCT-116 ISET** | |
| --- | --- | --- | --- | --- | --- | --- | --- | --- | --- | --- | --- | --- |
| gene | **Overlap**  **Known** | **type** | **Cat.** | **Coding**  **Consequence** | **chrs** | **Genome**  **position** | **c.DNA** | **protein** | **depth** | **var_%** | **depth** | **var_%** |
| ABL1 | COSM1674906 | SNP | A | missense | 9 | 133738370 | c.827A>G | p.Tyr276Cys | 5406 | 51.17 | 7492 | 49.67 |
| SMO | COSM13148 | SNP | A | missense | 7 | 128846374 | c.1210G>A | p.Val404Met | 5094 | 51.69 | 5618 | 50.5 |
| TP53 | COSM3766190 | SNP | A | missense | 17 | 7579472 | c.215C>G | p.Pro72Arg | 5273 | 98.82 | 4707 | 98.32 |
| CTNNB1 | COSM33668 | INDEL | A | inframe_3 | 3 | 41266133 | c.133_135delTCT | p.Ser45del | 3007 | 44.8 | 2837 | 49.35 |
| APC | COSM3760869 | SNP | A | synonymous | 5 | 112175770 | c.4425G>A | p.= (p.Thr1475Thr) | 2751 | 99.49 | 3306 | 99.4 |
| EGFR | COSM1451600 | SNP | A | synonymous | 7 | 55249063 | c.2361G>A | p.= (p.Gln787Gln) | 2644 | 99.77 | 2028 | 99.7 |
| RET | COSM4418406 | SNP | A | synonymous | 10 | 43613843 | c.2307G>T | p.= (p.Leu769Leu) | 4616 | 99.7 | 5563 | 99.53 |
| HRAS | COSM3752426 | SNP | A | synonymous | 11 | 534242 | c.81T>C | p.= (p.His27His) | 3118 | 99.81 | 3285 | 99.45 |
| FLT3 | COSM2070142 | SNP | A | synonymous | 13 | 28602367 | c.2001G>A | p.= (p.Gln667Gln) | 3815 | 51.27 | 4312 | 51.39 |
| IDH2 | COSM2139738 | SNP | A | synonymous | 15 | 90631825 | c.528C>T | p.= (p.Gly176Gly) | 3839 | 46.89 | 3691 | 47.01 |
| FLT3 | COSM3999060 | SNP | A | intronic | 13 | 28610183 | c.1310-3T>C |  | 4034 | 100 | 4984 | 99.94 |
| SMARCB1 | COSM1090 | SNP | A | intronic | 22 | 24176287 | c.1092-41G>A |  | 4920 | 49.41 | 4354 | 50.71 |
| FGFR1 |  | INDEL | A | frameshift | 8 | 38285932 | c.379delG | p.Asp127Metfs*25 | 2682 | 43.48 | 2789 | 41.77 |
| FGFR3 |  | SNP | C | synonymous | 4 | 1806187 | c.1206C>A | p.= (p.Pro402Pro) | 2933 | 6.1 | 2710 | 6.31 |
| FGFR3 |  | SNP | C | synonymous | 4 | 1807894 | c.1953G>A | p.= (p.Thr651Thr) | 2800 | 99.75 | 3495 | 99.89 |
| PDGFRA |  | SNP | C | synonymous | 4 | 55141055 | c.1701A>G | p.= (p.Pro567Pro) | 3129 | 99.9 | 3033 | 99.9 |
| ERBB4 |  | SNP | C | intronic | 2 | 212812097 | c.421+58A>G |  | 1867 | 100 | 1983 | 99.8 |
| KDR |  | SNP | C | intronic | 4 | 55946354 | c.3849-24C>A |  | 4366 | 48.31 | 4843 | 49.12 |
| KDR |  | SNP | C | intronic | 4 | 55980239 | c.798+54G>A |  | 2159 | 100 | 2139 | 99.58 |
| SMAD4 |  | SNP | C | intronic | 18 | 48586344 | c.955+58C>T |  | 4055 | 48.14 | 4093 | 48.99 |
| STK11 |  | SNP | C | intronic | 19 | 1220321 | c.465-51T>C |  | 2251 | 46.82 | 3325 | 50.47 |
| CSF1R |  | SNP | C | 3'UTR | 5 | 149433596 | c.*35_*36delCAinsTC |  | 384 | 97.14 | 238 | 96.22 |

Cat = Sophia DDM category of pathogenicity, Chrs = chromosome, var_% = percentage of variant

**Table E. Non-sense COSMIC mutant allele coverage, amplicon coverage and allele frequency measured by Ion Torrent^TM^ in WGA-amplified single cells enriched from blood by ISET^®^**

|  | **Sample** | **Single HCT116 (H1)** | **Single HCT116 (H2)** | **Single HCT116 (H3)** | **Pooled data 3 HCT116** | **WGA-Amplified HCT116 DNA** | **Unamplified bulk HCT116 DNA** | **Single leukocyte (L1)** | **Single leukocyte (L2)** | **Single leukocyte (L3)** | **Pooled data 3 leukocytes** | **Bulk DNA blood donor** |
| --- | --- | --- | --- | --- | --- | --- | --- | --- | --- | --- | --- | --- |
| **KRAS G13D** | **mutant allele frequency** | **38%** | **100%** | **100%** | **56%** | **42%** | **51%** | **0%** | **0%** | **ND** | **0%** | **0%** |
|  | mutant allele coverage | 37 | 11 | 29 | 77 | 59 | 995 | 0 | 0 | 0 | 0 | 0 |
|  | amplicon coverage | 97 | 11 | 29 | 137 | 140 | 1958 | 13 | 52 | 0 | 65 | 1942 |
| **PIK3CA H1047R** | **mutant allele frequency** | **73%** | **46%** | **32%** | **50%** | **46%** | **51%** | **0%** | **0%** | **0%** | **0%** | **0%** |
|  | mutant allele coverage | 710 | 453 | 312 | 1475 | 99 | 1010 | 0 | 0 | 0 | 0 | 0 |
|  | amplicon coverage | 979 | 981 | 986 | 2946 | 213 | 1996 | 280 | 524 | 498 | 1302 | 1996 |
| **SMO V404M** | **mutant allele frequency** | **55%** | **12%** | **67%** | **45%** | **53%** | **50%** | **0%** | **0%** | **0%** | **0%** | **0%** |
|  | mutant allele coverage | 1099 | 245 | 1346 | 2690 | 1064 | 1009 | 0 | 0 | 0 | 0 | 0 |
|  | amplicon coverage | 2000 | 1996 | 2000 | 5996 | 1998 | 2000 | 1992 | 1983 | 1986 | 5961 | 1998 |
| **KIT M541L** | **mutant allele frequency** | **0%** | **0%** | **0%** | **0%** | **0%** | **0%** | **57%** | **51%** | **57%** | **55%** | **48%** |
|  | mutant allele coverage | 0 | 0 | 0 | 0 | 0 | 0 | 1141 | 1009 | 1132 | 3282 | 967 |
|  | amplicon coverage | 1988 | 1996 | 1994 | 5978 | 1990 | 1999 | 1996 | 1989 | 1992 | 5977 | 1995 |
| **ALB Y257C** | **mutant allele frequency** | **17%** | **59%** | **67%** | **47%** | **51%** | **50%** | **0%** | **0%** | **0%** | **0%** | **0%** |
|  | mutant allele coverage | 303 | 1170 | 1054 | 2527 | 1016 | 999 | 0 | 0 | 0 | 0 | 0 |
|  | amplicon coverage | 1759 | 1998 | 1583 | 5340 | 2000 | 2000 | 1318 | 990 | 287 | 990 | 2000 |

**Table F. Main parameters and sensitivity of CTC filtration-based methods**

| **Method (Company or Academic laboratory)** | **Pressure type** | **Pression or Depression** | **Filter type** | **Pore size (µm)** | **Fixation** | **Sample dilution** | **Red blood cell lysis** | **Blood volume (mL)** | ***In vitro* Sensitivity (overall recovery, number of concentrations tested)^*1^** | **Reference** |
| --- | --- | --- | --- | --- | --- | --- | --- | --- | --- | --- |
| **ISET^®^ System** (Rarecells Diagnostic France) | Vaccum pump | - 10 kPa | track-etched | 8 | form. | 1:10 | yes | 10 | 1 CTC per 10 mL of blood (99.9%, 6) | this study |
|  | Vaccum pump | -3 to - 6 kPa | track-etched | 8 | none | 1:10 | yes | 10 | 1 CTC per mL of blood (90%, 4) | this study |
| **CTC Membrane Microfilter** (Cote’s lab, USA) | Manual Syringe | + 3.45 kPa | 2D parylene | 10 | form. | 1:2 | no | 7.5 | 5 CTC in 7.5 mL of blood  (89%, 1) | [1, 2] |
| **Screencell^®^ MB or CC** (Screencell, France) | Vacutainer tube | - 2.5 kPa | track-etched | 6.5 | none | 1:8 | yes | 6 | NA | [3, 4] |
| **Screencell^®^ Cyto** (Screencell, France) | Vacutainer tube | - 2.5 kPa | track-etched | 7.5 | form. | 1:7 | yes | 3 | 2 CTC in 1 mL of blood (74%, 2) | [3, 4] |
| **3D microfilter** (Cote’s lab, USA) | Manual Syringe | + 3.45 kPa | 3D parylene | 8 (top) 9 (bottom ) | none | 1:10 | no | 1 | NA (87%, 1) | [5] |
| NA (Terstappen’s lab, netherlands) | Syringe pump | + 1 kPa | track-etched | 8 | none | 1:4 | no, ^*2^ | 1 to 10 | 2 CTC in 1 mL of blood (67%, 6) | [6, 7] |
|  |  |  | microsieve | 5 |  |  |  |  | 2 CTC in 1 mL of blood (58%, 6) |  |
| **CellSieve^TM^** (Creativ Microtech, USA) | Vaccum pump | - 1.5 kPa | lithographic | 7 | optional | 1:2 | NA | 7.5 | NA (89 % for unfixed cells and 98% for fixed cells, 1) | [8] |
| **MetaCell^®^** (MetaCell, Czech Republic) | Capillarity | NA | track-etched | 8 | none | NA | NA | 8 | NA | [9] |

*1 Overall recovery= average recovery for the range of concentration tested, number of concentration tested (n>2 replicates for each concentration unless specified)

*2 elimination of red blood cells by centrifugation

Notes: methods that use Ficoll or equivalent prior to filtration or without any peer reviewed publication are not included in this table.

form. = formaldehyde, NA = not available

**Table G. CCC detection by ISET**^®^ **in the blood of healthy donors and patients with benign diseases**

| Reference | Number of cases | CCC detection | Types |
| --- | --- | --- | --- |
| [10] | 8 | 0 | healthy |
| [11] | 38 | 0 | healthy |
| [12] | 40 | 0 | healthy |
| [13] | 38 | 0 | healthy |
| [13] | 10 | 0 | nevi |
| [14] | 1 | 1 | benign nevus |
| [15] | 16 | 0 | healthy |
| [16] | 39 | 0 | healthy |
| [17] | 59 | 0 | healthy |
| [18] | 49 | 0 | healthy |
| [18] | 190 | 10 | various benign diseases including parathyroid and thyroid adenoma |
| [19] | 40 | 0 | healthy |
| [20] | 6 | 0 | healthy |
| [21] | 10 | 0 | choroidal nevi |
| [22] | 30 | 0 | healthy |
| [23] | 77 | 0 | healthy |
| [24] | 21 | 0 | Benign nevus |
| [24] | 16 | 0 | healthy |
| TOTAL | **688** | **11** | **98.4 % Overall specificity** |

**Table H. Studies reporting prognostic value of CCC/CCM detected by ISET**^®^ **and ISET**^®^ **longitudinal follow-up studies**

| **Reference** | **Number of patients** | **Type** | **Stage** | **Percentage of patients with CTC/ CTM** | **Follow-up** | **Cut-off** | **Conclusion** | **Type (endpoint)** |
| --- | --- | --- | --- | --- | --- | --- | --- | --- |
| [11] | 44 | Hepato-Cellular Carcinoma | Localized | 52.3% | 50 +/- 48 weeks | 1 CCC/CCM per 3 mL | Patients without CCCs/CCM: increased survival compared to patients with CCCs/CCM (P =.01, chi2 test), worse prognosis for patients with more than 3 CCCs as compared to those with 1 to 3 CCCs | Prognostic (OS) |
| [16] | 208 | Non-Small Cell Lung Cancer | All (I to IV) | 36.5% | 24 months (12-41) | 50 CNHC per 6 mL | Number of CNHCs significantly associated with shorter OS and worse DFS (P= 0.002, and P= 0.001), for both early-stage I+II and later-stage III+IV-resectable NSCLCs (P = 0.05, and P < 0.0001) | Prognostic (OS, DFS) |
| [19] | 210 | Non-small cell Lung Cancer | All (I to IV) | 49.5% | 15 months (1–28) | 1 CNHC per 10 mL | Patients without CNHC had a significantly longer DFS compared to patients with CNHC (p < 0.0001; log rank test= 33.07), presence of CNHC was a significant independent prognostic factor for shorter DFS (HR, 1.372; 95% CI, 1.123–3.286; p= 0.006): | Prognostic (OS, DFS) |
| [25] | 1 | Non-Small Cell Lung Cancer | IV | - |  | NA | Longitudinal CCC enumeration by ISET consistent with progression of the disease (while cytokeratin-based CTC enumeration is unrelated) | Longitudinal follow-up |
| [21] | 31 | Uveal Melanoma | All (I to IV) | 54.8% | 55 months (24-180) | >10 CCC and CCM per 10 mL | Significantly different DFS (Log Rank test p = 0.012) and OS (Log Rank test p = 0.017) between subjects with less than 10 CCC/10 mL of blood and subjects with more than 10 CCC/10 mL of blood and CCM. | Prognostic (OS, DFS) |
| [23] | 168 | Chronic Obstructive Pulmonary Disease | IA at diagnosis (NSCLC) | 3% | 4 years | 1 CNHC per 10 mL | The five COPD patients with CTCs detected by cytopathology analysis after blood-enrichment at baseline developed a lung cancer that was diagnosed at follow-up, 1 to 4 years after CTCs were first detected. | Prognostic |
| [26] | 52 | Colorectal cancer | IV | 82.7% | 7.9 months (1.2-19.4) | 2 CCC/mL | Patients who had CCCs count above cutoff showed more CCC TYMS expression (p=0.02); CCC TYMS positivity was persistent, but not significant in patients who had disease progression (p=0.07), | Prognostic / Predictive (PFS) |
| [27] | 26 | Colorectal cancer | IV | 88.5% | 5-14 months | <3 CTCs/7.5mL | Patients with less CCC (below cutoff) and KRAS wt in tumor: higher PFS and OS than patients with more CCCs and KRAS mutation in tumor (P= 0.001 and P= 0.004) | Prognostic / Predictive (OS, PFS) |
| [28] | 8 (4 ROS1-rearranged) | Non-Small Cell Lung Cancer | Metastatic | 100.0% | 30-90 days | - | Longitudinal follow-up for 5 patients, heterogeneity of responses to crizotinib in CCC subsets and of FISH patterns | Longitudinal follow-up (resistance) |
| [29] | 50 | Pancreatic cancer | I, II and IV | 90.0% | 14 months (8.4-16.4) | > 1 mesenchymal CCC in 1 mL | Presence of mesenchymal CCCs was significantly associated with cancer recurrence [HR 2.78, 95% confidence interval (CI) 1.31–5.88, P = 0.01], Shorter time to recurrence (9. vs 13.5 months) in patients with mesenchymal-like CCCs (P = 0.02). | Prognostic (PFS, OS) |
|  |  |  |  |  |  | > 1 epithelial CCC in 1 mL | Epithelial CCCs : significantly associated with worse survival compared with patients without CCCs (median survival 13.7 mo vs not reached, P = 0.008) |  |
| [24] | 128 | Cutaneous Melanoma | IIIB, IIIC, IV | 85.2% | 12 months (3-18) | > 1 CCM in 10 mL | OS significantly decreased in patients with CCMs alone or CCMs and iCCCs at baseline in comparison to patients with no CCMs or with iCCCs alone independently of the therapeutic strategy Presence of CCMs at baseline (P = 0.022): independent predictor of poor OS | Prognostic (OS) |
| [30] | 34 | Colorectal cancer | IV | 88.2% | 9.1 months (7.2-11) | >1 MRP1 positive CCCs in 1 mL | Among patients treated with irinotecan-based chemotherapy, 4 out of 19 cases with MRP1 positive CCCs showed a worse PFS in comparison to those with MRP1 negative CCCs (2.1 months vs. 9.1 months; P= 0.003). | Prognostic/ Predictive (PFS) |
| [31] | 1 | Non-Small Cell Lung Cancer | Metastatic (T1aN2M1b) | 100% | 2 months | - | Baseline 4 CTC by CellSearch, 0 post-therapy; ISET post-therapy >150 CCCs | Longitudinal follow-up |

CNHC: circulating non-hematological cells include cells with benign, uncertain and malignant features.

Overall Survival (OS) is defined by the NIH-NCO as the percentage of patient in a study or treatment group who are still alive for a certain period of time after they were diagnosed with or started treatment for a disease, such as cancer.

Progression-free survival (PFS) is defined by the NIH-NCO as length of time during and after the treatment of a disease, such as cancer, that a patient lives with the disease but it does not get worse. Disease-free survival (DFS) is defined by the NIH-NCO as the length of time after primary treatment for a cancer that the patient survives without any signs or symptoms of that cancer.

Hazard ratio (HR) is defined by the NIH-NCO as, a measure of how often a particular event happens in one group compared to how often it happens in another group, over time. A hazard ratio of one means that there is no difference in survival between the two groups. A hazard ratio of greater than one or less than one means that survival was better in one of the groups.

Prognostic biomarker: biomarker that can be used to estimate the chance of recovery from a disease or the chance of the disease recurring.

Predictive biomarker: biomarker that can be used to help predict whether a person’s cancer will respond to a specific treatment. Predictive factor may also describe something that increases a person’s risk of developing a condition or disease.

**Table I. CCC/CCM detected by ISET**^®^ **in the blood of cancer patients**

| Reference | Number of patients | Disease type | Stage | Timing of blood sampling^‡^ | Number of patients with CCC or CCM | % of patients with CCC or CCM | Mean* CCC number (range) per 10 mL | Mean* CCM number (range) per 10 mL |
| --- | --- | --- | --- | --- | --- | --- | --- | --- |
| [10] | 7 | HCC | na | Before and after surgery | 3 | 43 % | 0 | 8.6 (0 to 40) |
| [11] | 44 | HCC | M0 (localized or diffuse) | Prior treatment | 23 | 52 % | na | na (3.3 to 33) |
| [12] | 44 | BC | I to III | Before surgery | 12 | 27 % | 85 (positive patients only) (1 to 300) | |
| [13] | 87 | CM | All (I to IV) | Before surgery or during treatment for M1 patients | 23 | 26 % | 8 (median) (2.5 to 35) | |
|  | 5 | non-melanoma skin tumors | na |  | 0 | 0% | 0 | 0 |
| [15] | 16 | UM | M0 (small, medium and large) | Before therapy | 5 | 31 % | 24 (positive patients only) (7.5 to 58) | |
| [16] | 208 | NSCLC | All (I to IV) | Before surgery | 76 | 37 % | na | na |
| [17] | 250 | NSCLC | All (I to IV) | Before surgery | 102 | 49 % | na | na |
| [18] | 569 | NSCLC, BC, CC, KC, HNC, PM, Sarc, CM, EC | All (I to IV) | Mostly before surgery | 245 | 43 % | na | na |
| [19] | 210 | NSCLC | All (I to IV) | Before surgery | 104 | 50 % | 34 (positive patients only) (1 to 150) | |
| [32] | 6 | 3 NSCLC, 3SCLC | IIIB or IV | Unknown | 6 | 100 % | na | na |
| [33] | 20 | BC | M1 | Unknown | 17 | 85% | 5 (0 to 27) | |
| [33] | 20 | PrC, | M1 | Unknown | 20 | 100% | 38 (median) (1 to 331) | |
| [33] | 20 | NSCLC | M1 | Unknown | 20 | 100% | 12.5 (median) (1 to >133) | |
| [20] | 6 | NSCLC | M1 | Variable after diagnosis | 6 | 100 % | 87 (16-190) | 18 (0-40) |
| [34] | 40 | NSCLC | IIIA to IV | Before therapy | 32 | 80 % (38 % with CCM) | 71 (0-1393) | 6 (0-13) |
| [35] | 27 | PaC | M1 or inoperable | Variable, 6 weeks off therapy | 24 | 89 % | 35 (0-320) | na, 3 patients with CCM |
| [36] | 20 | SCLC | Limited and extensive | Before therapy | 20 | NA | na | 34 CTM over 20 patients |
| [37] | 87 | NSCLC | All (I to IV) | Before therapy | 87 | 100 % | na | na |
| [38] | 98 | CM | IIIB, IIIC, IV | Before surgery | 87 | 89% | na | na |
| [28] | 32 | NSCLC | M1 | Baseline or under crizotinib | 32 | 100 % | 190 (40 to 450) | |
| [25] | 1 | NSCLC | IV | Baseline or before new cycle of chemo | 1 | 100 % | 735 to 1285 over the course of the disease | |
| [39] | 90 | CM | IV | Before therapy | 51 | 57 % | 14 (0 to 110) | na, 12 patients with CCM |
| [21] | 31 | UM | All (I to IV) | Before therapy | 17 | 55 % | 8 (median) (2 to 50) | na, 8 patients with CCM |
| [40] | 8 | PrC | All (Gleason 5 to 10) | Before therapy | 8 | 100 % | 0 to 300 | na |
| [41] | 19 | HCC | All (I to IV) | Baseline (4-weeks off therapy) | 19 | 100 % | 101 (25 to 271) | na |
| [22] | 11 | Sarc | All (I to IV) | Unknown | 11 | 100 % | 27 (2.5 to 35) | na |
| [42] | 4 | BC | M1, invasive ductal carcinoma | Unknown | 4 | 100 % | na | na |
| [26] | 52 | CRC | IV | Before new line of therapy | 43 | 83 % | 20 (median) (0 to 310) | na |
| [27] | 26 | CRC | IV | Before new line of therapy | 23 | 88 % | 20 (median) (0 to 140) | na |
| [43] | 8 | NSCLC | M1 | Baseline or under crizotinib | 4/4 ROS-1 rearranged, 8/8 total | na | 123 ROS1 rearranged (median) (80 to 183) | |
| [29] | 50 | PaC | I, II and IV | Before surgery | 45 | 90% | 850 (median) (0 to 3000) | na |
| [44] | 68 | CM | All (I to IV) | At diagnosis, before surgery and therapy | 18 | 27 % | na | na |
| [24] | 128 | CM | IIIB, IIIC, IV | Before first line of therapy | 109 | 85 % | 6 (4 to 16) | 4 (3 to 9) |
| [30] | 34 | CRC | IV | Before new line of therapy | 30 | 88% | 20 (median) (0 to 310) |  |
| [31] | 1 | NSCLC | T1aN2M1b | At diagnosis and after therapy | 1 | 100% | >1500 | na |
| Total | **2347** |  |  |  | **1328** |  |  | |

Abbreviations: HCC: hepatocellular carcinoma, BC: breast cancer, PrC: prostate cancer, PaC: pancreatic cancer, NSCLC: Non-Small Cell Lung Cancer, SCLC: Small Cell Lung cancer, CC: Colorectal cancer, KC: Kidney cancer, HNC: Head and Neck carcinoma, Esophageal carcinoma: EC, Pleural Mesothelioma: PM, Sarcoma: Sarc, UM: uveal melanoma, CM: cutaneous melanoma,

M0: localized, M1: metastatic, na: not available

‡ if several blood sampling time points are reported, number of patients with CCCs/CCMs and CCC/CTMs number are only indicated for the baseline time point

* if average CCC and CCM numbers are not available, the median or range are provided as indicated in the table. Usually the mean is calculated over the whole population of patients (including patients without CCCs), unless specified otherwise in the table.

# Supplemental references

1.	Zheng S, Lin H, Liu JQ, Balic M, Datar R, Cote RJ, et al. Membrane microfilter device for selective capture, electrolysis and genomic analysis of human circulating tumor cells. J Chromatogr A. 2007;1162(2):154-61.

2.	Lin HK, Zheng S, Williams AJ, Balic M, Groshen S, Scher HI, et al. Portable filter-based microdevice for detection and characterization of circulating tumor cells. Clin Cancer Res. 2010;16(20):5011-8.

3.	Desitter I, Guerrouahen BS, Benali-Furet N, Wechsler J, Janne PA, Kuang Y, et al. A new device for rapid isolation by size and characterization of rare circulating tumor cells. Anticancer Res. 2011;31(2):427-41.

4.	Cayre Y, inventorDevice and method for isolating and cultivating live cells on a filter or extracting the genetic material therof2009.

5.	Zheng S, Lin HK, Lu B, Williams A, Datar R, Cote RJ, et al. 3D microfilter device for viable circulating tumor cell (CTC) enrichment from blood. Biomed Microdevices. 2011;13(1):203-13.

6.	Coumans FA, van Dalum G, Beck M, Terstappen LW. Filter characteristics influencing circulating tumor cell enrichment from whole blood. PLoS One. 2013;8(4):e61770.

7.	Coumans FA, van Dalum G, Beck M, Terstappen LW. Filtration parameters influencing circulating tumor cell enrichment from whole blood. PLoS One. 2013;8(4):e61774.

8.	Adams DL, Zhu P, Makarova OV, Martin SS, Charpentier M, Chumsri S, et al. The systematic study of circulating tumor cell isolation using lithographic microfilters. RSC Adv. 2014;9:4334-42.

9.	Bobek V, Kacprzak G, Rzechonek A, Kolostova K. Detection and cultivation of circulating tumor cells in malignant pleural mesothelioma. Anticancer Res. 2014;34(5):2565-9.

10.	Vona G, Sabile A, Louha M, Sitruk V, Romana S, Schutze K, et al. Isolation by size of epithelial tumor cells : a new method for the immunomorphological and molecular characterization of circulatingtumor cells. Am J Pathol. 2000;156(1):57-63.

11.	Vona G, Estepa L, Beroud C, Damotte D, Capron F, Nalpas B, et al. Impact of cytomorphological detection of circulating tumor cells in patients with liver cancer. Hepatology. 2004;39(3):792-7.

12.	Pinzani P, Salvadori B, Simi L, Bianchi S, Distante V, Cataliotti L, et al. Isolation by size of epithelial tumor cells in peripheral blood of patients with breast cancer: correlation with real-time reverse transcriptase-polymerase chain reaction results and feasibility of molecular analysis by laser microdissection. Hum Pathol. 2006;37(6):711-8.

13.	De Giorgi V, Pinzani P, Salvianti F, Panelos J, Paglierani M, Janowska A, et al. Application of a filtration- and isolation-by-size technique for the detection of circulating tumor cells in cutaneous melanoma. J Invest Dermatol. 2010;130(10):2440-7.

14.	De Giorgi V, Pinzani P, Salvianti F, Grazzini M, Orlando C, Lotti T, et al. Circulating benign nevus cells detected by ISET technique: warning for melanoma molecular diagnosis. Arch Dermatol. 2010;146(10):1120-4.

15.	Pinzani P, Mazzini C, Salvianti F, Massi D, Grifoni R, Paoletti C, et al. Tyrosinase mRNA levels in the blood of uveal melanoma patients: correlation with the number of circulating tumor cells and tumor progression. Melanoma Res. 2010;20(4):303-10.

16.	Hofman V, Bonnetaud C, Ilie MI, Vielh P, Vignaud JM, Flejou JF, et al. Preoperative circulating tumor cell detection using the isolation by size of epithelial tumor cell method for patients with lung cancer is a new prognostic biomarker. Clin Cancer Res. 2011;17(4):827-35.

17.	Hofman V, Long E, Ilie M, Bonnetaud C, Vignaud JM, Flejou JF, et al. Morphological analysis of circulating tumour cells in patients undergoing surgery for non-small cell lung carcinoma using the isolation by size of epithelial tumour cell (ISET) method. Cytopathology. 2011.

18.	Hofman VJ, Ilie MI, Bonnetaud C, Selva E, Long E, Molina T, et al. Cytopathologic detection of circulating tumor cells using the isolation by size of epithelial tumor cell method: promises and pitfalls. Am J Clin Pathol. 2011;135(1):146-56.

19.	Hofman V, Ilie MI, Long E, Selva E, Bonnetaud C, Molina T, et al. Detection of circulating tumor cells as a prognostic factor in patients undergoing radical surgery for non-small cell lung carcinoma: Comparison of the efficacy of the CellSearch Assay and the isolation by size of epithelial tumor cell method. Int J Cancer. 2011;2011.

20.	Lecharpentier A, Vielh P, Perez-Moreno P, Planchard D, Soria JC, Farace F. Detection of circulating tumour cells with a hybrid (epithelial/mesenchymal) phenotype in patients with metastatic non-small cell lung cancer. Br J Cancer. 2011;105(9):1338-41.

21.	Mazzini C, Pinzani P, Salvianti F, Scatena C, Paglierani M, Ucci F, et al. Circulating tumor cells detection and counting in uveal melanomas by a filtration-based method. Cancers (Basel). 2014;6(1):323-32.

22.	Chinen LTD MC, Abdallah EA, Ocea LMM, Buim ME, Breve NM, Gasparini Jr JL, Fanelli MF, Paterlini-Bréchot P. Isolation, detection, and immunomorphological characterization of circulating tumor cells (CTCs) from patients with diffrent types of sarcoma using isolation by size of tumor cells: a window on sarcoma-cell invasion. OncoTargets and Therapy. 2014;7:1609-17.

23.	Ilie M, Hofman V, Long-Mira E, Selva E, Vignaud JM, Padovani B, et al. "Sentinel" circulating tumor cells allow early diagnosis of lung cancer in patients with chronic obstructive pulmonary disease. PLoS One. 2014;9(10):e111597.

24.	Long E, Ilie M, Bence C, Butori C, Selva E, Lalvee S, et al. High expression of TRF2, SOX10, and CD10 in circulating tumor microemboli detected in metastatic melanoma patients. A potential impact for the assessment of disease aggressiveness. Cancer Med. 2016.

25.	Chinen LT, de Carvalho FM, Rocha BM, Aguiar CM, Abdallah EA, Campanha D, et al. Cytokeratin-based CTC counting unrelated to clinical follow up. J Thorac Dis. 2013;5(5):593-9.

26.	Abdallah EA, Fanelli MF, Buim ME, Machado Netto MC, Gasparini Junior JL, Souza ESV, et al. Thymidylate synthase expression in circulating tumor cells: a new tool to predict 5-fluorouracil resistance in metastatic colorectal cancer patients. Int J Cancer. 2015;137(6):1397-405.

27.	Buim ME, Fanelli MF, Souza VS, Romero J, Abdallah EA, Mello CA, et al. Detection of KRAS mutations in circulating tumor cells from patients with metastatic colorectal cancer. Cancer Biol Ther. 2015;16(9):1289-95.

28.	Pailler E, Adam J, Barthelemy A, Oulhen M, Auger N, Valent A, et al. Detection of circulating tumor cells harboring a unique ALK rearrangement in ALK-positive non-small-cell lung cancer. J Clin Oncol. 2013;31(18):2273-81.

29.	Poruk KE, Valero V, 3rd, Saunders T, Blackford AL, Griffin JF, Poling J, et al. Circulating Tumor Cell Phenotype Predicts Recurrence and Survival in Pancreatic Adenocarcinoma. Ann Surg. 2016.

30.	Abdallah EA, Fanelli MF, Souza ESV, Machado Netto MC, Gasparini Junior JL, Vilarim Araujo D, et al. MRP1 expression in CTCs confers resistance to irinotecan-based treatment in metastatic colorectal cancer. Int J Cancer. 2016.

31.	Morrow CJ, Trapani F, Metcalf RL, Bertolini G, Hodgkinson CL, Khandelwal G, et al. Tumourigenic Non-Small Cell Lung Cancer Mesenchymal Circulating Tumour Cells - A Clinical Case Study. Ann Oncol. 2016.

32.	Hou JM, Krebs M, Ward T, Sloane R, Priest L, Hughes A, et al. Circulating tumor cells as a window on metastasis biology in lung cancer. Am J Pathol. 2011;178(3):989-96.

33.	Farace F, Massard C, Vimond N, Drusch F, Jacques N, Billiot F, et al. A direct comparison of CellSearch and ISET for circulating tumour-cell detection in patients with metastatic carcinomas. Br J Cancer. 2011;105(6):847-53.

34.	Krebs MG, Hou JM, Sloane R, Lancashire L, Priest L, Nonaka D, et al. Analysis of circulating tumor cells in patients with non-small cell lung cancer using epithelial marker-dependent and -independent approaches. J Thorac Oncol. 2012;7(2):306-15.

35.	Khoja L, Backen A, Sloane R, Menasce L, Ryder D, Krebs M, et al. A pilot study to explore circulating tumour cells in pancreatic cancer as a novel biomarker. Br J Cancer. 2012;106(3):508-16.

36.	Hou JM, Krebs MG, Lancashire L, Sloane R, Backen A, Swain RK, et al. Clinical significance and molecular characteristics of circulating tumor cells and circulating tumor microemboli in patients with small-cell lung cancer. J Clin Oncol. 2012;30(5):525-32.

37.	Ilie M, Long E, Butori C, Hofman V, Coelle C, Mauro V, et al. ALK-gene rearrangement: a comparative analysis on circulating tumour cells and tumour tissue from patients with lung adenocarcinoma. Ann Oncol. 2012;23(11):2907-13.

38.	Hofman V, Ilie M, Long-Mira E, Giacchero D, Butori C, Dadone B, et al. Usefulness of immunocytochemistry for the detection of the BRAF(V600E) mutation in circulating tumor cells from metastatic melanoma patients. J Invest Dermatol. 2013;133(5):1378-81.

39.	Khoja L, Shenjere P, Hodgson C, Hodgetts J, Clack G, Hughes A, et al. Prevalence and heterogeneity of circulating tumour cells in metastatic cutaneous melanoma. Melanoma Res. 2014;24(1):40-6.

40.	Cummings J, Sloane R, Morris K, Zhou C, Lancashire M, Moore D, et al. Optimisation of an immunohistochemistry method for the determination of androgen receptor expression levels in circulating tumour cells. BMC Cancer. 2014;14:226.

41.	Morris KL, Tugwood JD, Khoja L, Lancashire M, Sloane R, Burt D, et al. Circulating biomarkers in hepatocellular carcinoma. Cancer Chemother Pharmacol. 2014;74(2):323-32.

42.	LeBleu VS, O'Connell JT, Gonzalez Herrera KN, Wikman H, Pantel K, Haigis MC, et al. PGC-1alpha mediates mitochondrial biogenesis and oxidative phosphorylation in cancer cells to promote metastasis. Nat Cell Biol. 2014;16(10):992-1003, 1-15.

43.	Pailler E, Auger N, Lindsay CR, Vielh P, Islas-Morris-Hernandez A, Borget I, et al. High level of chromosomal instability in circulating tumor cells of ROS1-rearranged non-small-cell lung cancer. Ann Oncol. 2015;26(7):1408-15.

44.	Salvianti F, Orlando C, Massi D, De Giorgi V, Grazzini M, Pazzagli M, et al. Tumor-Related Methylated Cell-Free DNA and Circulating Tumor Cells in Melanoma. Front Mol Biosci. 2016;2:76.
